# Reaction hijacking of tyrosine tRNA synthetase as a whole-of-life-cycle antimalarial strategy

**DOI:** 10.1126/science.abn0611

**Published:** 2022-06-02

**Authors:** Stanley C. Xie, Riley D. Metcalfe, Elyse Dunn, Craig J. Morton, Shih-Chung Huang, Tanya Puhalovich, Yawei Du, Sergio Wittlin, Shuai Nie, Madeline R. Luth, Liting Ma, Mi-Sook Kim, Charisse Flerida A. Pasaje, Krittikorn Kumpornsin, Carlo Giannangelo, Fiona J. Houghton, Alisje Churchyard, Mufuliat T. Famodimu, Daniel C. Barry, David L. Gillett, Sumanta Dey, Clara C. Kosasih, William Newman, Jacquin C. Niles, Marcus C.S. Lee, Jake Baum, Sabine Ottilie, Elizabeth A. Winzeler, Darren J. Creek, Nicholas Williamson, Michael W. Parker, Stephen L. Brand, Steven P. Langston, Lawrence R. Dick, Michael D.W. Griffin, Alexandra E. Gould, Leann Tilley

**Affiliations:** 1 Department of Biochemistry and Pharmacology, Bio21 Molecular Science and Biotechnology Institute, The University of Melbourne, Melbourne, VIC 3010, Australia; 2 Takeda Development Center Americas, Inc., Cambridge, Massachusetts 02139, USA; 3 Swiss Tropical and Public Health Institute, 4051 Basel, Switzerland; 4 University of Basel, 4003 Basel, Switzerland; 5 Melbourne Mass Spectrometry and Proteomics Facility, Bio21 Molecular Science and Biotechnology Institute, The University of Melbourne, Melbourne, VIC 3010, Australia; 6 Department of Pediatrics, School of Medicine, University of California, San Diego, La Jolla, California 92093, USA; 7 Department of Biological Engineering, Massachusetts Institute of Technology, Cambridge, United States; 8 Parasites and Microbes Programme, Wellcome Sanger Institute, Hinxton, CB10 1SA, United Kingdom; 9 Drug Delivery, Disposition and Dynamics, Monash Institute of Pharmaceutical Sciences, Monash University, Parkville, VIC 3052, Australia; 10 Department of Life Sciences, Imperial College London, London SW7 2AZ, UK; 11 St. Vincent’s Institute of Medical Research, Fitzroy, VIC 3065, Australia; 12 Medicines for Malaria Venture, PO Box 1826, 20, Route de Pré-Bois, 1215, Geneva 15, Switzerland; 13 Seofon Consulting, 30 Tucker Street, Natick, Massachusetts 01760, USA

## Abstract

Aminoacyl tRNA synthetases (aaRSs) are attractive drug targets. Here we show that class I and II aaRSs are previously unrecognized targets for AMP-mimicking nucleoside sulfamates. The target enzyme catalyzes the formation of an inhibitory amino acid-sulfamate conjugate, via a reaction-hijacking mechanism. We identified adenosine 5′-sulfamate (AMS) as a broad specificity compound that hijacks a range of aaRSs; and ML901 as a specific reagent that hijacks a single aaRSs in the malaria parasite, *Plasmodium falciparum*, namely, tyrosine RS (*Pf*YRS). ML901 exerts whole-of-life-cycle killing activity with low nanomolar potency and single dose efficacy in a mouse model of malaria. X-ray crystallographic studies of plasmodium and human YRSs reveal differential flexibility of a loop over the catalytic site that underpins differential susceptibility to reaction-hijacking by ML901.

Diseases caused by infectious organisms pose an enormous threat to global health, food security and sustainable development. Malaria is one such debilitating disease, caused by protist parasites of the genus *Plasmodium*. Every year at least 200 million new infections of *P. falciparum* malaria are established, causing more than 600,000 deaths (1). Current antimalarial treatments are rapidly losing efficacy; and standard-of-care artemisinin combination therapies fail to cure infections in ~50% of patients in some regions of Asia (2). Clinically validated resistance to artemisinins has now been detected in Africa (3), where most malaria deaths occur. New treatments with novel modes of action are urgently needed to overcome existing resistance, expand possible treatment options and enable more effective combination therapies.

## Adenosine 5'-sulfamate exhibits broad specificity reaction hijacking, revealing potential antimalarial drug targets

**Figure 1:**
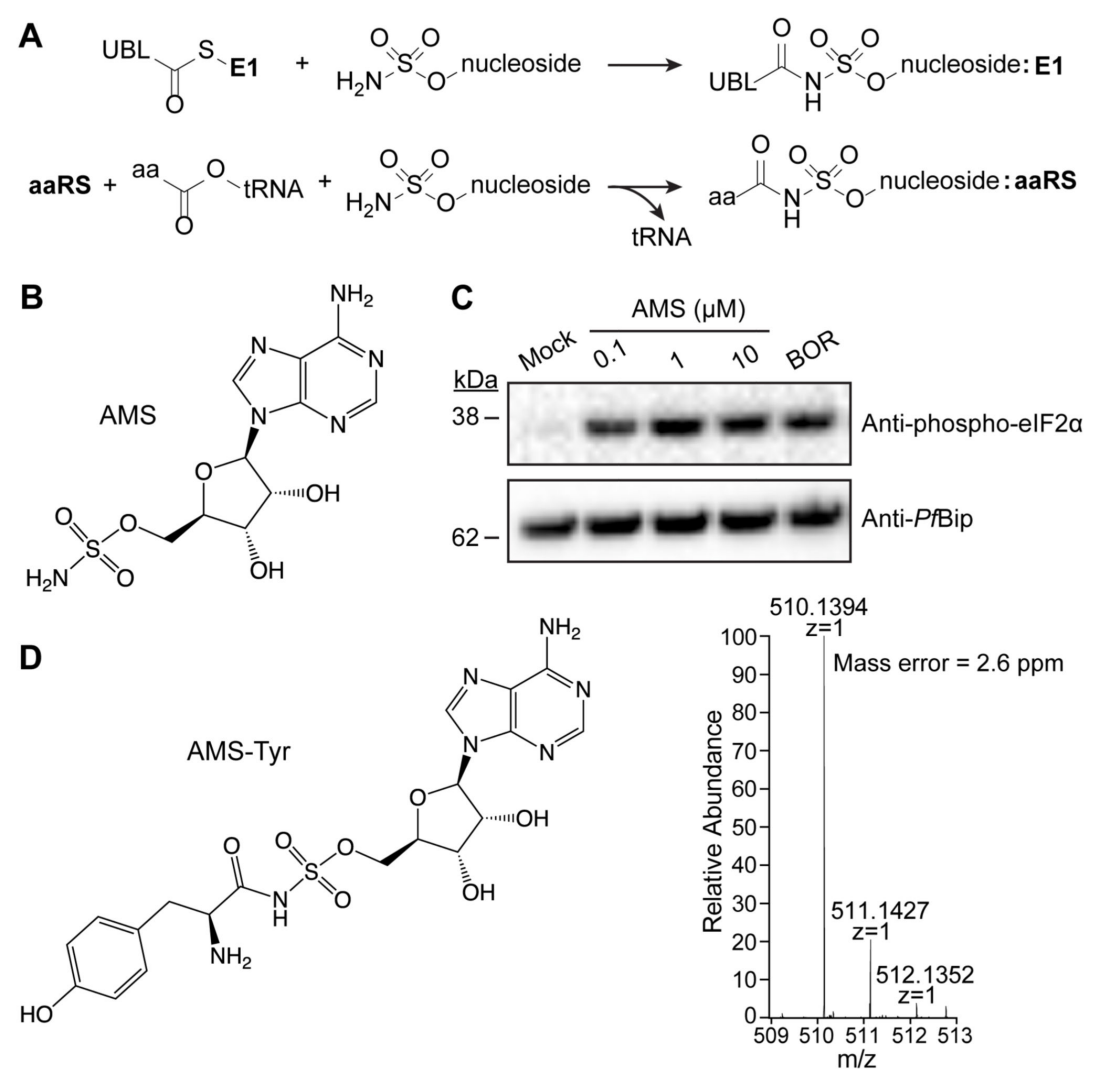
AMS-treated infected RBCs reveals aaRSs as potential targets. (**A**) E1 enzymes can catalyze attack of the sulfamate nitrogen on the carbonyl carbon of the thioester bond between the UBL and the E1 to form a UBL conjugate. aaRSs could catalyze nucleoside sulfamate attack on activated amino acids to form an amino acid adduct. (**B**) Structure of 5′-adenylate sulfamate. (**C**) Trophozoite stage parasites were incubated with DMSO (Mock), different concentrations of AMS, or, borrelidin (BOR; a threonyl-tRNA synthetase inhibitor). Western blots of lysates were probed for phosphorylated-eIF2α with *Pf*BiP as a loading control. The blot is typical of data from three independent experiments. (**D**) *P. falciparum-infected* RBCs were treated with 10 μM AMS for 3 h. Extracts were subjected to LCMS analysis identifying the Tyr-AMS conjugate. The profile is typical of data from three independent experiments.

Nucleoside sulfamates, such as the investigational drug Pevonedostat (4), inhibit ubiquitin-like protein (UBL) activating enzymes (E1s) by forming covalent conjugate inhibitors with the enzyme-bound UBL. The E1s catalyze nucleophilic attack of the sulfamate nitrogen on the thio-ester bond between the UBL and the E1 (Fig. 1A, fig. S1). Until now, attack on the thio-ester bonds of UBLs was the only known example of this type of inhibitor mechanism. However, naturally occurring nucleoside sulfamates and derivatives, such as nucleocidin (5), 2-Cl-adenosine sulfamate (6, 7) and adenosine 5′-sulfamate (AMS) (8), exhibit inhibitory activity against bacteria (6–8), which lack E1 enzymes. The compounds are broadly toxic and have been reported to inhibit protein synthesis (8, 9), but until now the mechanisms underlying these activities were unknown.

We explored the activity of adenosine 5′-sulfamate (AMS, Fig. 1B), a close mimic of AMP, as a potential starting point for identifying antimalarial compounds. We found that AMS is highly cytotoxic (IC_50_72h_ = 1.8 nM) to *P. falciparum* cultures, with an efficacy similar to that of current front-line drug, dihydroxyartemisinin (DHA), but is also cytotoxic to mammalian cell lines, such as HCT116 (IC_50_72h_ = 26 nM) (table S1). We found that treatment of *P. falciparum* cultures with AMS triggers eIF2α phosphorylation (Fig. 1C), a hallmark of stress caused by either accumulation of unfolded proteins or uncharged tRNAs (10). Like E1 enzymes, aminoacyl tRNA synthetases (aaRSs) are adenylate-forming enzymes (AFEs). They catalyze the transformation of amino acids into AMP conjugates, and then into aminoacyl-tRNAs, to supply protein synthesis. Given the reported effects on protein translation (8, 9), we considered the possibility that aaRSs might be able to catalyze nucleophilic attack of AMS on their cognate aminoacyl tRNAs (Fig. 1A).

The proposed mechanism would be expected to generate AMS-amino acid conjugates (Fig. 1A), so we used targeted mass spectrometry to search for the predicted conjugates in *P. falciparum*-infected red blood cells (RBCs) and cultured human cells (HeLa) that had been treated with 10 μM AMS for 2-3 h (see Supplementary Material for full methods). Following Folch extraction of lysates, the aqueous phase was subjected to liquid chromatography-coupled mass spectrometry (LCMS) and the anticipated masses for the 20 possible amino acid conjugates were interrogated. In *P. falciparum*, the extracts yielded a strong signal for AMS-Tyr (Fig. 1D), with matching precursor ion *m/z* (< 3 ppm) and anticipated fragmentation spectrum (fig. S2A). MS peaks were also detected for the adducts of Asn, Asp, Ser, Thr, Gly, Ala, Lys and Pro (fig. S2B). In the mammalian cell line, AMS conjugates were identified for Asn, Pro, Ala, Thr, Asp and Tyr (fig. S3). No peaks were detected in control samples. These data are consistent with aaRSs catalyzing nucleoside sulfamate attack on the activated oxy-ester bonds of their cognate aminoacyl tRNAs (Fig. 1A), Thus, both class I and class II aaRSs are potentially susceptible to inhibition via the reaction hijacking mechanism.

## Identifying a nucleoside sulfamate with potent and specific antimalarial activity

**Figure 2:**
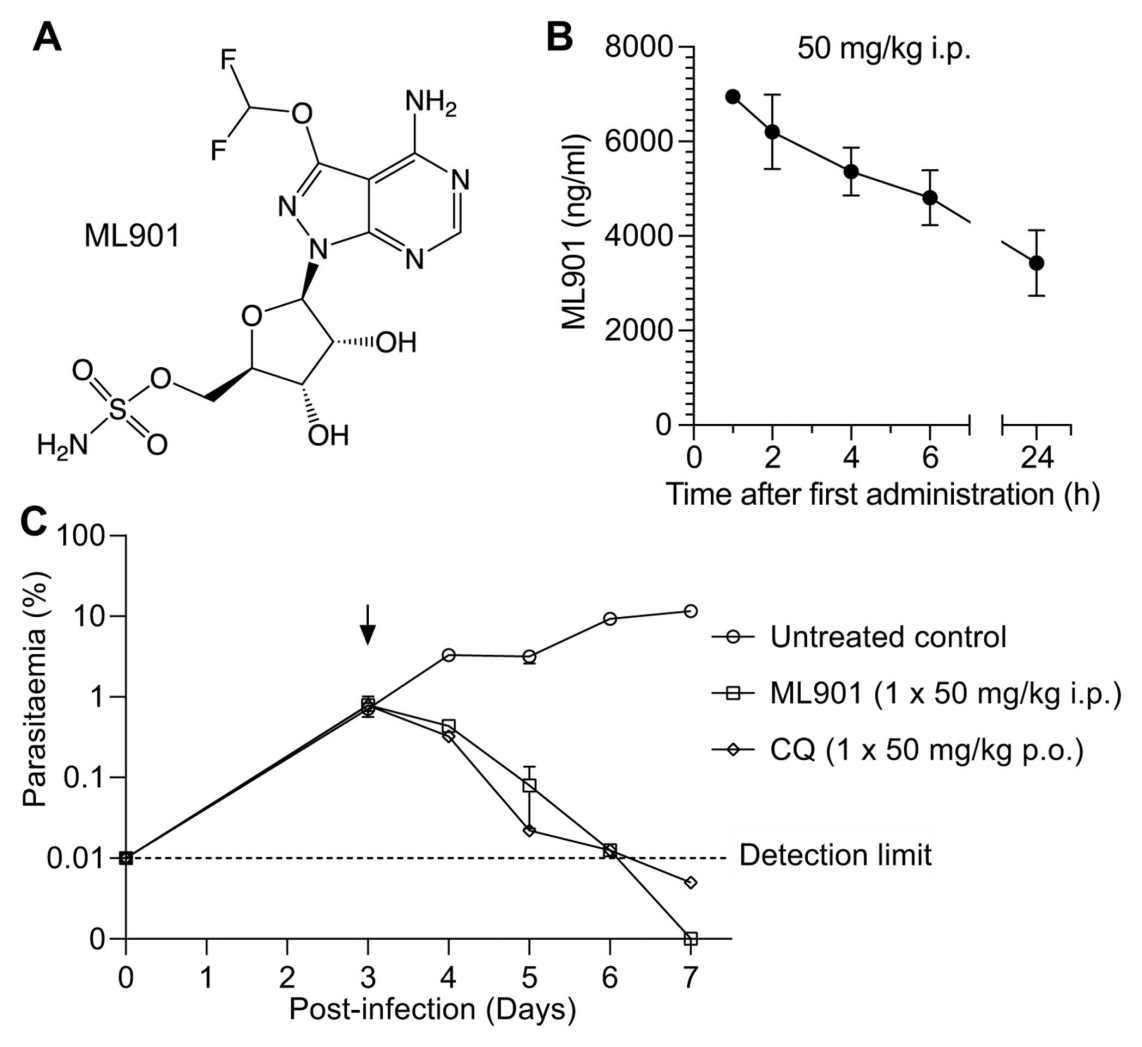
ML901 exhibits potent activity against *P. falciparum in vivo*. (**A**) Structure of pyrazolopyrimidine ribose sulfamate, ML901. (**B**) Pharmacokinetics profile (in blood) over the first day for SCID mice engrafted with human RBCs infected with *P. falciparum* following treatment with ML901 at 50 mg/kg i.p. (**C**) Therapeutic efficacy of ML901 in the SCID mouse *P. falciparum* model, dosed with ML901 at 50 mg/kg i.p. in comparison with gold standard antimalarial, chloroquine, dosed at 50 mg/kg p.o..

In an effort to identify aaRS-targeting nucleoside sulfamates with narrower specificity, we screened 2314 sulfamates from the Takeda compound library (Cambridge, MA, USA) for inhibition of the growth of *P. falciparum*. The library included compounds that were synthesized as potential inhibitors of Atg7, an E1 that activates UBLs, including the Atg8s (11). We identified several pyrazolopyrimidine sulfamates with a 7-position substituent (exemplar ML901; Fig. 2A) that possess potent activity against *P. falciparum*. The ML901 50% inhibitory concentration (IC_50_72h_ = 2.0 ± 0.1 nM) is similar to that for DHA (table S1).

ML901 was tested for cytotoxicity against different mammalian cell lines (table S1) and showed 800- to 5,000-fold selectivity towards *P. falciparum* (>1,000-fold higher selectivity than AMS). ML901 retained activity against all strains of *P. falciparum* tested, regardless of their resistance profile and geographical origin (table S2). It potently inhibited transmissible male gametes (table S2); and prevented development of *P. falciparum* in primary human hepatocytes (table S2). We confirmed that ML901 exerts activity against human Atg7 (IC_50_ = 33 nM); but has much weaker activity against other E1 enzymes (table S3), as previously reported for nucleoside sulfamates with a substitution at the 7-position (11); and consistent with the low mammalian cell cytotoxicity. By contrast, AMS is a potent inhibitor of each of the E1s tested (table S3). The rat pharmacokinetic profile of ML901 (fig. S4; table S3) is encouraging, with low blood clearance and a long terminal half-life in blood (T_1/2∞_ = 41 h) following intravenous or oral dosing.

We determined the *in vivo* antimalarial efficacy of ML901 in severe combined immune deficient (SCID) mice, engrafted with *P. falciparum* infected human RBCs (12, 13), which is the gold standard for testing *in vivo* efficacy of malaria drug candidates. A single dose (50 mg/kg i.p.) results in excellent exposure (area under the curve = 580 μM.h; Fig. 2B) and achieves reduction of parasitemia to baseline (Fig. 2C), with no evidence of toxicity. The clearance rate is similar to that of chloroquine (CQ; 50 mg/kg p.o.).

## ML901 selectively targets plasmodium tyrosine tRNA synthetase

To identify the target of ML901 in *P. falciparum*, extracts of infected RBCs that had been treated with 3 μM ML901 (3 h) were subjected to LCMS to search for amino acid-ML901 conjugates. An LCMS peak corresponding to protonated ML901-Tyr (m/z = 576.1324) was detected. Synthetic ML901-Tyr was generated and spiked into the untreated *P. falciparum* lysate to confirm the peak assignment (fig. S5). None of the other 19 possible amino acid conjugates were detected.

**Figure 3:**
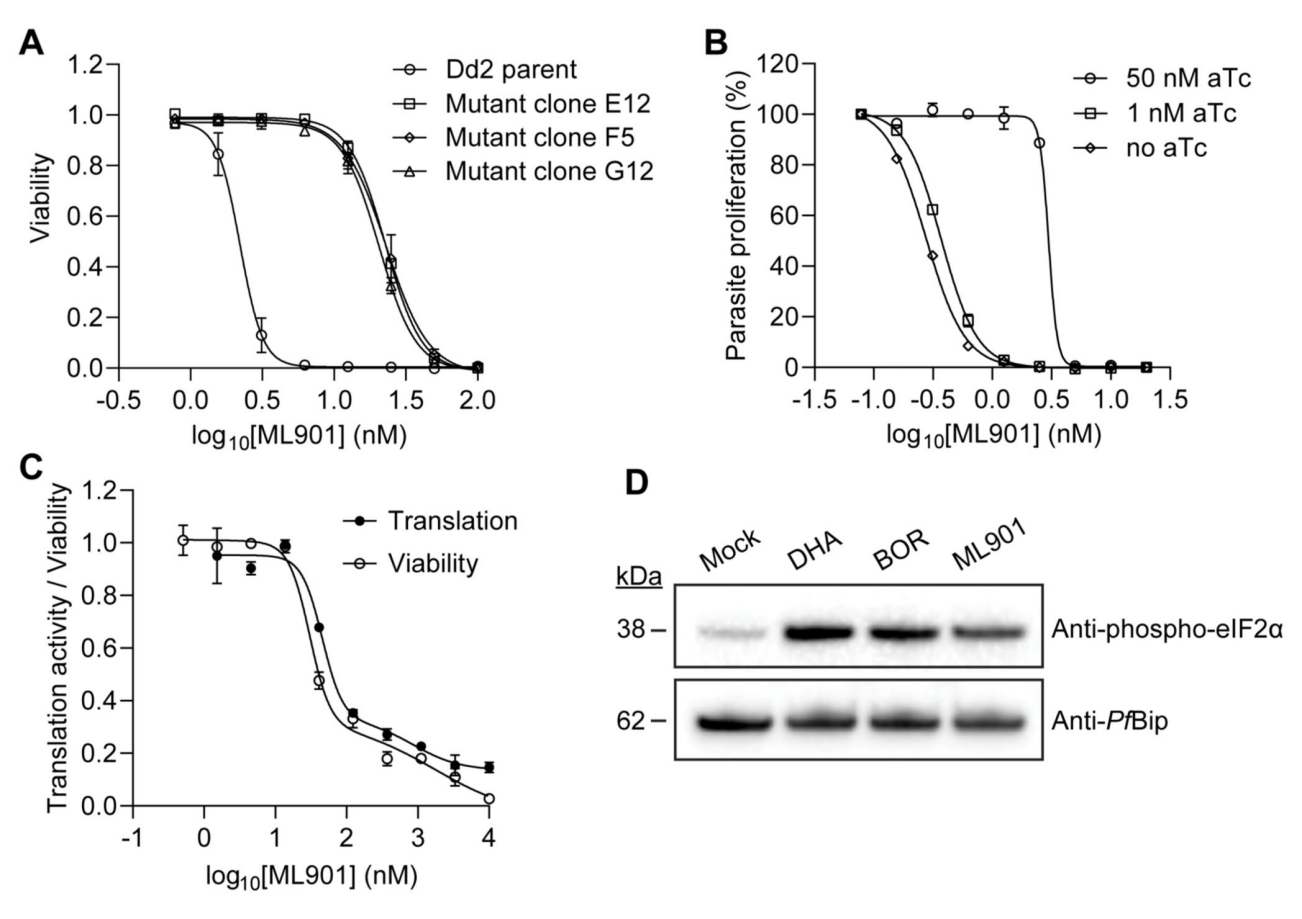
ML901 targets *Pf*YRS and inhibits protein translation. (**A**,**B**) Sensitivity to ML901 exposure (72-h) for a cloned wildtype line (Dd2) and 3 CRISPR-edited clones harboring *Pf*YRS_S234C_ (**A**) or an aptamer-regulatable *Pf*YRS line upon addition of aTc, with data normalized to a no drug control (**B**). See table S5 for data values. (**C**) RBCs infected with schizont stage (43-46 h p.i.) *P. falciparum* (Cam3.II-rev) were exposed to ML901 for 3 h. Protein translation was assessed in the second two hours of the incubation, via the incorporation of OPP. Aliquots of inhibitor-exposed cultures were washed and returned to cultures, and viability was estimated at the trophozoite stage of the next cycle. IC_50_ (Translation) = 65 nM, IC_50_ (Viability) = 56 nM. Data are representative of three independent experiments. Error bars correspond to the range of technical duplicates. (**D**) Schizont stage Cam3.II_rev parasites were incubated with DMSO (Mock), 1 μM DHA, 200 nM borrelidin (BOR) or 200 nM ML901 for 3 h and Western blots of lysates were probed for phosphorylated-eIF2α with *PfBiP* as a loading control. The blot is typical of data from three independent experiments.

To determine whether *P. falciparum* tyrosine tRNA synthetase (*Pf*YRS) is the critical target in *P*. falciparum, we subjected parasite cultures (3D7 line) to increasing concentrations of the compound over a period of 4 months; retrieving parasites with 10-fold reduced sensitivity (ML901 IC_50_72h_ = 28 nM) (fig. S6A). Whole genome sequencing of one parental and three resistant clones revealed nine newly acquired mutations in the resistant clones, three of which were in PF3D7_0807900 (position 403,556), corresponding to a Ser234Cys mutation in cytoplasmic *Pf*YRS (table S4). Insertion of an additional Asn in an Asn repeat in kinesin-5 is considered unlikely to be functionally significant (see table S4). No mutations were observed in any other AFE, including *P. falciparum* Atg7. Transfectants were generated (Dd2 parent) harboring the *Pf*YRS_S234C_ mutation (fig. S6B-D), which recapitulated the resistance phenotype (*i.e*., 10-fold decreased sensitivity) (Fig. 3A; table S5). Aptamer-induced downmodulation of *Pf*YRS decreased the growth rate compared with the control (fig. S6E) and increased sensitivity to ML901 (Fig. 3B; table S5); but not to DHA or Thr-RS inhibitor, borrelidin (fig S6F; table S5). The remarkable potency of ML901 against the knockdown parasites (IC_50_ = 0.4 nM) both validates *Pf*YRS as the target and points to an extremely potent inhibitory interaction.

ML901 inhibits protein translation in *P. falciparum* schizonts (as monitored by O-propargyl-puromycin incorporation (14); Fig. 3C), consistent with *Pf*YRS being the target. The IC_50_3h_ value (50 nM) correlates well with that for parasite killing potency (Fig. 3C). Another protein translation inhibitor, cycloheximide, has a similar profile (fig. S7A), while the folate pathway inhibitor, WR99210, kills parasites with no immediate effect on protein translation (fig. S7B). ML901 triggers eIF2α phosphorylation in wildtype *P. falciparum* (Cam3.II_rev; Fig. 3D), consistent with the presence of uncharged tRNA (10, 15). In eIK1 (GCN2 equivalent (16)) knockout parasites, the amino acid starvation pathway is disrupted and ML901 treatment does not result in eIF2α phosphorylation (fig. S7C), consistent with an aaRS target (fig. S7D).

**Figure 4:**
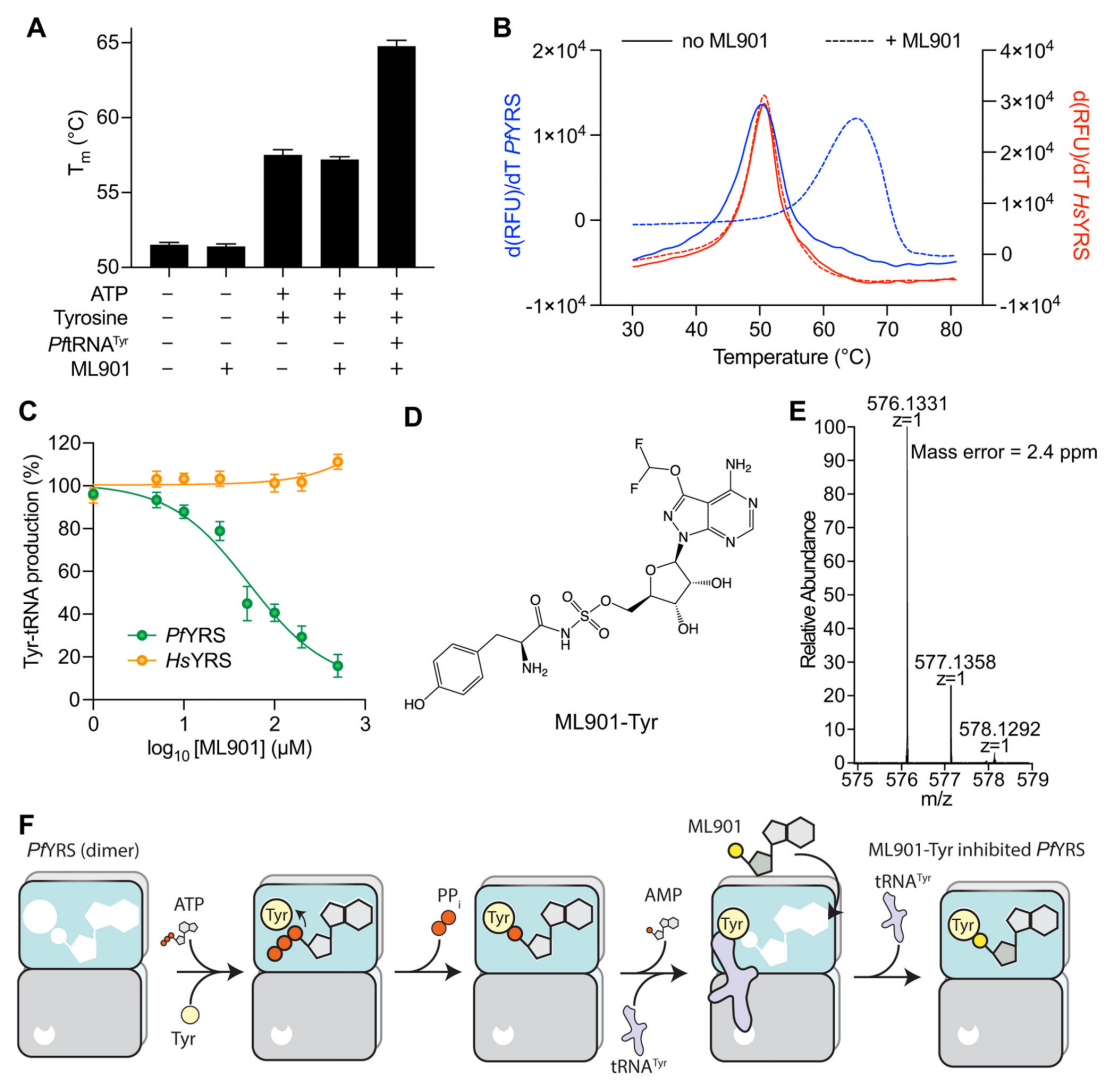
ML901 inhibits *Pf*YRS by a reaction-hijacking mechanism. (**A**) The apparent melting temperature (T_m_) of *Pf*YRS after incubation at 37°C for 3 h with the indicated reactants: ML901 (50 μM), ATP (50 μM), tyrosine (100 μM), *Pf*tRNA^Tyr^ (4 μM). Data represent the average of three independent assays and error bars correspond to SD. (**B**) First derivatives of melting curves for *Pf*YRS and *Hs*YRS with or without pre-incubation with ML901 (50 μM), ATP (50 μM), tyrosine (100 μM) and *Pf*tRNA^Tyr^ (4 μM). Data is representative of three independent assays. (**C**) Effects of increasing concentrations of ML901 on tyrosine acylation of the cognate tRNA^Tyr^ by *Pf*YRS and *Hs*YRS with YRS (0.25 μM), ATP (10 μM), tyrosine (100 – 200 μM), cognate tRNA^Tyr^ (24 μM) and pyrophosphatase (1 unit/mL), at 37°C for 1 h. IC_50_ (*Pf*YRS) = 53 μM; IC_50_ (*Hs*YRS) > 500 μM. Data represent the average of 8 independent assays and error bars correspond to SEM. (**D**) Structure of ML901-Tyr. (**E**) *Pf*YRS was incubated with ML901 (50 μM), ATP (10 μM), tyrosine (20 μM) and *Pf*tRNA^Tyr^ (8 μM). Following urea denaturation and TFA precipitation, the supernatant was subjected to LCMS analysis, revealing the expected protonated ML901-Tyr ion. The profile is typical of data from three independent experiments. (**F**) Schematic of reaction-hijacking mechanism.

YRSs from wildtype (*Pf*YRS), mutant (*Pf*YRS_S234C_) and human (mature *Hs*YRS (17)) were produced in *Escherichia coli*. Biophysical characterization revealed well-folded dimers (fig. S8,9; table S6). *Pf*tRNA^Tyr^ and *Hs*tRNA^Tyr^ (18) were generated by *in vitro* transcription (fig. S10). When ML901 was incubated with *Pf*YRS in the presence of all other substrates (*i.e*., Tyr, ATP and tRNA^Tyr^), the apparent protein melting point (T_m_), measured by differential scanning fluorimetry (DSF), increased by a remarkable 15°C (Fig. 4A,B; fig. S11A). The increase in thermal stability is even greater than that induced by the tightly bound adenylate intermediate, AMP-Tyr (table S7). Whereas formation of the AMP adenylate requires only Tyr and ATP, the thermal stabilization induced by ML901 required all three substrates (Tyr, ATP and tRNA^Tyr^). This result is consistent with a hijacking mechanism that requires charged tRNA^Tyr^ (Tyr-tRNA^Tyr^; see Fig. 1A). Importantly, recombinant *Hs*YRS was not stabilized in the presence of ML901 plus substrates, suggesting the inhibitory species is not produced by, or does not bind to, the human enzyme (Fig. 4B, red curves). The mutant *Pf*YRS_S234C_ was less well stabilized (fig. S11A, table S7), suggesting weaker binding of the inhibitory species, consistent with the mutant parasite’s resistance to ML901.

## ML901 exerts its activity by hijacking the active site-bound reaction product

We examined the abilities of the recombinant YRSs to consume ATP, *i.e*., to form and release AMP-Tyr in the initial reaction phase. *Hs*YRS and *Pf*YRS_S234C_ show higher activity (in the absence of tRNA) than *Pf*YRS (fig. S11B). This difference suggests that AMP-Tyr is bound less tightly to the *Hs* and mutant *Pf*YRS active sites. Upon addition of the cognate tRNA^Tyr^, ATP consumption is enhanced, consistent with productive aminoacylation. Acylation of the cognate tRNA^Tyr^ to radiolabeled tyrosine (19), occurs at a similar level in all three enzymes (fig. S11C). ML901 inhibits ATP consumption by *Pf*YRS when added in the presence of *Pf*tRNA^Tyr^ but not in its absence (fig. S11D). Similarly, ML901 inhibits tRNA^Tyr^ acylation to tyrosine *in vitro* by *Pf*YRS, but not by *Hs*YRS (Fig. 4C). Synthetically generated ML901-Tyr conjugate is able to inhibit the activity of both *Pf*YRS and *Hs*YRS (fig. S11E,F), suggesting that, although *Hs*YRS is unable to generate the ML901-Tyr inhibitor, it can bind the preformed conjugate.

To confirm that recombinant *Pf*YRS can generate the ML901-Tyr conjugate, we incubated *Pf*YRS with substrates and ML901, then precipitated the tRNA and protein. The supernatant was subjected to LCMS analysis and we detected a peak at m/z 576.1331 Da (Fig. 4D,E), consistent with the expected protonated ML901-Tyr ion, and confirmed using the synthetic ML901-Tyr standard (fig. S12A-C). Under the conditions used, *Pf*YRS generates more ML901-Tyr than *Pf*YRS_S234C_ dimer (fig. S12E); and no ML901-Tyr was detected when *Hs*YRS was incubated with ML901 and substrates. Thus, it appears that the ability of *Pf*YRS to catalyze formation of the ML901-Tyr conjugate is the primary factor controlling selectivity versus *Hs*YRS and potency versus *Pf*YRS_S234C_.

## A structured loop over the *Pf*YRS active site facilitates reaction hijacking

**Figure 5:**
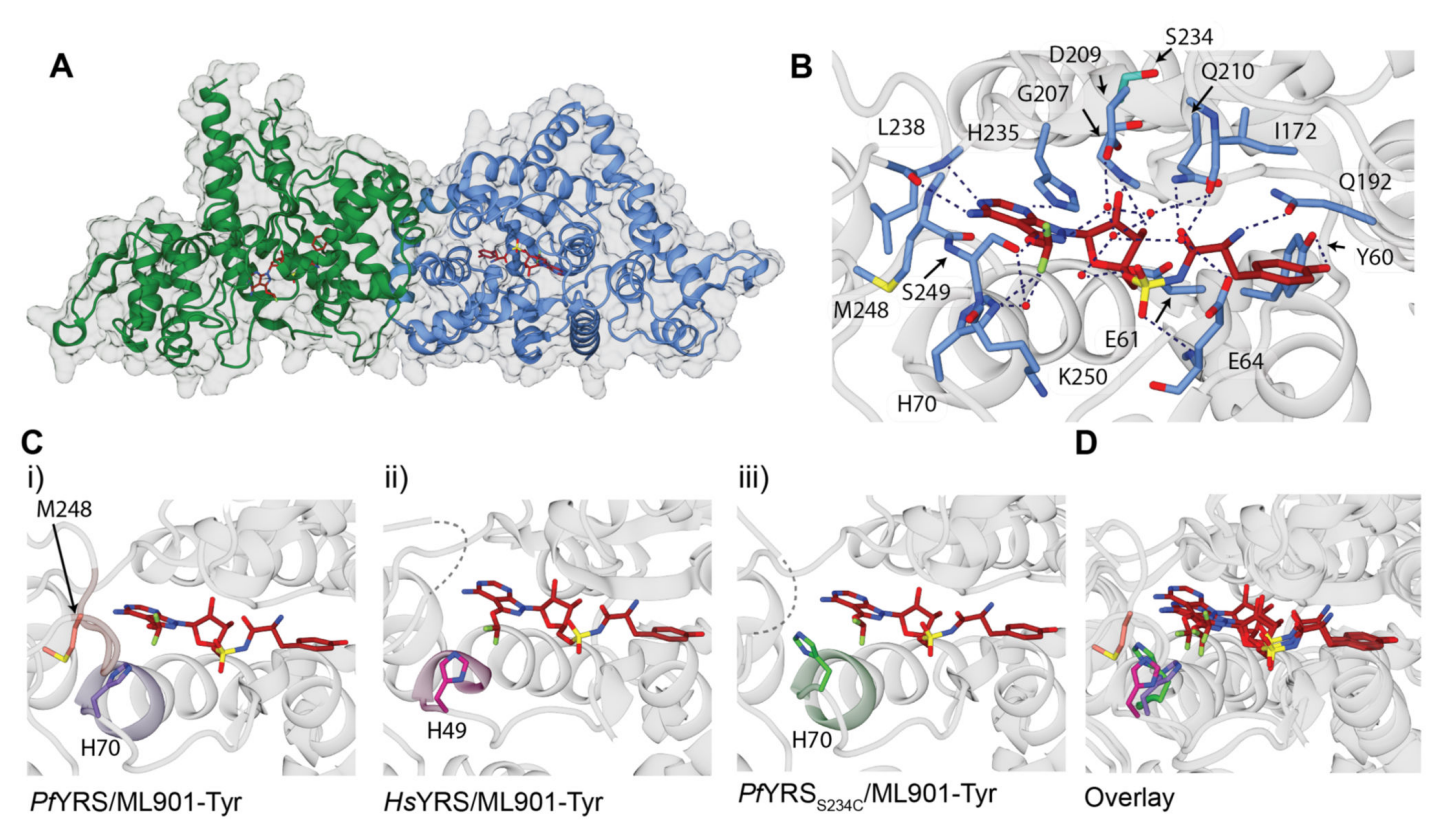
Structural analysis of YRSs reveals the determinants of potency and specificity. (**A**) The structure of the dimeric *Pf*YRS/ML901-Tyr complex showing chain A (green), chain B (blue), and bound ML901-Tyr (red stick representation). (**B**) Inhibitor/active site interactions for the B chain. (**C**) (i) The *Pf*YRS chain B active site highlighting the “HIGH” (_70_HIAQ_73_; light purple) and “KMSKS” (_247_KMSKS_251_; light brown) motifs with bound ML901-Tyr (colored by atom type). M248 and H70 are positioned to interact. (ii) Active site of *Hs*YRS with bound ML901-Tyr highlighting the “HIGH” motif (_49_HVAY_52_; light pink). (iii) Active site of *Pf*YRS_S234C_ with bound ML901-Tyr highlighting the “HIGH” (_70_HIAQ_73_; light green). Unmodelled loops are shown in Cii and Ciii as dashed lines. (**D**) Overlay of *Pf*YRS (B chain), *Hs*YRS, and *Pf*YRS_S234C_ showing the different configurations adopted by His70/His49. *Pf*YRS His70, purple; *Hs*YRS His49, pink; *Pf*YRS_S234C_ His70, green.

To obtain crystals of ML901-Tyr-bound *Pf*YRS, we incubated His-tagged *Pf*YRS with tyrosine, ATP and ML901 in the presence of tRNA and then purified the complex using nickel affinity and gel filtration chromatography. The crystal structure (refined at 2.15-Å resolution) revealed a homodimer organization with clear density for the ML901-Tyr ligand (Fig. 5A, fig. S13A).

*Pf*YRS is a Class I aaRS, characterized by a catalytic domain that adopts a Rossmann fold (residues 18–260) linked to a C-terminal domain (residues 261–370) that is involved in recognition of the anticodon stem of tRNA^Tyr^. *Pf*YRS contains the two motifs characteristic of the catalytic domain of Class I (sub-class c) tRNA synthetases: “HIGH” and “KMSKS” (_70_HIAQ_73_ and _247_KMSKS_251_ in *Pf*YRS). The overall structure of the ML901-Tyr/*Pf*YRS complex is very similar to our structure of *Pf*YRS with the native ligand, AMP-Tyr (fig. S14, table S8) and the previously published structure (19).

ML901-Tyr makes many interactions with active site residues, involving the pyrazolopyrimidine amine, ribose hydroxyls, sulfamate, and tyrosine (Fig. 5B; fig. S13B), which underpin the tight binding affinity; and potency. ML901-Tyr is present in the active sites of both monomers in the dimer (fig. S13C,D,J,K), but differences are observed with respect to the KMSKS loop, which forms a flap over the adenylate/ML901 binding site (Fig. 5B,Ci, fig. S13C-F). In the B chain, His70 (of _70_HIAQ_73_) makes close contact with Met248 in the KMSKS loop (Fig. 5Ci) and is well defined in the electron density (fig. S13F). In the A chain, part of the KMSKS loop is not well defined (fig. S13E), suggesting that the A chain loop is more mobile, and His70 adopts a side chain rotamer that would clash with Met248 if the loop was structured as in chain B (fig. S13F-I). In combination, these observations indicate different conformations of the KMSKS loop in individual monomers within the dimer. By contrast, the KMSKS loops of both monomers of AMP-Tyr-bound *Pf*YRS (fig. S14) are well defined, with the electron density clearly showing contacts between His70 and Met248 in both subunits.

In the published structure of tyrosine-bound *Hs*YRS (PDB: 4QBT (20)) the equivalent “KMSSS” loop is not modelled, suggesting that it is mobile. We were not able to generate a structure of *Hs*YRS in complex with enzyme-generated ML901-Tyr as *Hs*YRS does not catalyze formation of the conjugate. However, we were able to form crystals of *Hs*YRS in the presence of synthetic ML901-Tyr (fig. S15), consistent with our observation that the preformed conjugate can inhibit *Hs*YRS activity (fig. S11F). Although we observed clear density for ML901-Tyr (fig. S15F), the _222_KMSSS_226_ loops were not defined in the electron density (Fig. 5Cii; fig. S15D,E). Moreover, His49 (the equivalent of *Pf*YRS His70) adopts a position (Fig. 5D, magenta) similar to that in chain A of ML901-Tyr-bound *Pf*YRS (compare figures S13G,H and S15E), further suggesting that this configuration is associated with increased loop mobility. A comparison of the interaction networks (fig. S13B-D, S15B,C) reveals notably fewer interactions with the ML901 moiety in *Hs*YRS compared with *Pf*YRS; and specific interactions were poorly conserved between the two enzymes.

We also solved the structure of *Pf*YRS_S234C_ in complex with synthetic ML901-Tyr (fig. S16). Similar to *Hs*YRS, the KMSKS loops of both monomers were not defined in the electron density (fig. S16E,F), and His70 adopts a rotamer that is not consistent with a structured KMSKS loop (Fig. 5Ciii,D, green; compare figures S13G,H and S16G,H). Potency and selectivity of ML901 for *Pf*YRS thus appears to be associated with a stabilized loop over the active site. That is, the decreased susceptibility of *Hs*YRS and *Pf*YRS_S234C_ to reaction hijacking by ML901 is associated with mobility of the KMSSS/KMSKS loop, which is, in turn, associated with rotation of the His49/70 side chain. These conformational changes may promote dissociation of the charged tRNA, thereby preventing the hijacking reaction.

The pyrazolopyrimidine sulfamate chemotype is an attractive starting point for a malaria drug discovery program, based on our observation that the specific inhibition of *Pf*YRS by ML901 is lethal to disease-causing and transmissible stages of *P. falciparum*, and that ML901 exhibits a long *in vivo* half-life, underpinning its single-dose efficacy in a murine model of human malaria. Further exploration of substitutions at the 7-position of the pyrazolopyrimidine sulfamates class is expected to identify compounds with reduced activity against human Atg7, and thus even higher specificity for plasmodium. We note that the HIAQ and KMSKS motifs are conserved across apicomplexan and kinetoplastid parasites but not in metazoan organisms (fig. S17). This suggests that ML901-like compounds could exhibit cross-pathogen inhibitory activity. Use of the sulfamates in a drug combination could prevent evolution of resistant mutants.

Our finding that nucleoside sulfamates can hijack Class I and Class II tRNA aaRSs, as well as E1s, opens up the possibility of designing bespoke small molecular weight, membrane permeable AFE inhibitors with adjustable specificity. In addition to charging tRNA and activating ubiquitin, AFEs are involved in activating fatty acids for degradation, biosynthesis of natural products, and other diverse pathways (21). Thus, nucleoside sulfamates may find applications in a broad range of infectious, metabolic and neurodegenerative diseases.

## Supplementary Material

Supplementary Materials

## Data Availability

Coordinate files and structure factors have been deposited in the PDB: *Pf*YRS/AMP-Tyr: 7ROR; *Pf*YRS/ML901-Tyr: 7ROS; *Pf*YRS_S234C_/ML901-Tyr: 7ROT; *Hs*YRS/ML901-Tyr: 7ROU. All other data are available in the main text or the supplementary materials. The structure and synthesis of ML901 are detailed in the paper. For supply of materials developed as part of this work, please contact the corresponding authors. A Materials Transfer Agreement may be required for some materials.

## One-Sentence Summary

AMP-mimicking nucleoside sulfamates hijack the charging of tRNAs with amino acids, resulting in highly specific anti-infective agents.
