# Supplementary material for: Reaction hijacking of tyrosine tRNA synthetase as a whole-of-life-cycle antimalarial strategy
Source: Science. Author manuscript; Available in PMC 2022 Sep 22. (PMC7613620; doi:10.1126/science.abn0611)
Supplement: Supplementary Materials [file EMS154346-supplement-Supplementary_Materials.pdf]

Supplementary Information for

Reaction hijacking of tyrosine tRNA synthetase as a new whole-of-life-cycle antimalarial strategy

Stanley C. Xie[1+], Riley D. Metcalfe[1+], Elyse Dunn[1], Craig J. Morton[1], Shih-Chung Huang[2], Tanya Puhalovich[1], Yawei Du[1], Sergio Wittlin[3,4], Shuai Nie[5], Madeline R. Luth[6], Liting Ma[2], Mi-Sook Kim[2], Charisse Flerida A. Pasaje[7], Krittikorn Kumpornsin[8], Carlo Giannangelo[9], Fiona J. Houghton[1], Alisje Churchyard[10], Mufuliat T. Famodimu[10], Daniel C. Barry[1], David L. Gillett[1], Sumanta Dey[7], Clara C. Kosasih[1], William Newman[1], Jacquin C. Niles[7], Marcus C.S. Lee[8], Jake Baum[10], Sabine Ottilie[6], Elizabeth A. Winzeler[6], Darren J. Creek[9], Nicholas Williamson[5], Michael W. Parker[1,11], Stephen L. Brand[12], Steven P. Langston[2¥], Lawrence R. Dick[1,13¥], Michael D.W. Griffin[1¥], Alexandra E. Gould[2¥*], Leann Tilley[1 ¥*]

[1]Department of Biochemistry and Pharmacology, Bio21 Molecular Science and Biotechnology Institute, The University of Melbourne, Melbourne, VIC 3010, Australia; [2]Takeda Development Center Americas, Inc., Cambridge, Massachusetts 02139, USA; [3]Swiss Tropical and Public Health Institute, 4051 Basel, Switzerland; [4]University of Basel, 4003 Basel, Switzerland; [5]Melbourne Mass Spectrometry and Proteomics Facility, Bio21 Molecular Science and Biotechnology Institute, The University of Melbourne, Melbourne, VIC 3010, Australia; [6]Department of Pediatrics, School of Medicine, University of California, San Diego, La Jolla, California 92093, USA; [7]Department of Biological Engineering, Massachusetts Institute of Technology, Cambridge, United States; [8]Parasites and Microbes Programme, Wellcome Sanger Institute, Hinxton, CB10 1SA, United Kingdom; [9]Drug Delivery, Disposition and Dynamics, Monash Institute of Pharmaceutical Sciences, Monash University, Parkville, VIC 3052, Australia; [10]Department of Life Sciences, Imperial College London, London SW7 2AZ, UK; [11]St. Vincent's Institute of Medical Research, Fitzroy, VIC 3065, Australia; [12]Medicines for Malaria Venture, PO Box 1826, 20, Route de Pré-Bois, 1215, Geneva 15, Switzerland; [13]Seofon Consulting, 30 Tucker Street, Natick, Massachusetts 01760, USA

This PDF file includes:

Supplementary Figures 1 to 17

Supplementary Tables 1 to 8

Supplementary Methods, including chemical synthesis and characterization

SI References

Supplementary Figures

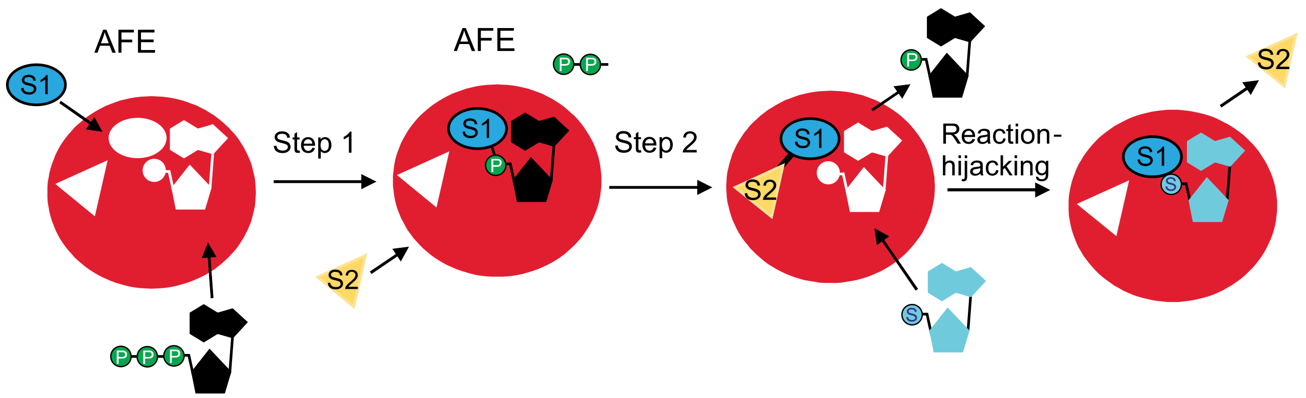

Supplementary Figure 1. Proposed mechanism for adenylate-forming enzyme (AFE) hijacking. An AFE binds ATP and a carboxylic acid substrate (S1, *e.g.* UBL carboxy terminus, amino acid or fatty acid) and catalyzes the formation of an AMP conjugate, as a reaction intermediate (Step 1). The intermediate is thereby primed for nucleophilic attack by a second substrate (S2), generating ester, thioester or amide products with the release of AMP (Step 2). A nucleoside sulfamate binds to the AMP vacated site and the enzyme catalyzes a nucleophilic attack on the reaction product. For E1 enzymes, the product of the reaction is a stable conjugate of the UBL and nucleoside sulfamate (*e.g.* Nedd-8-Pevonedistat) that resembles the natural adenylate intermediate and binds tightly to the enzyme inhibiting further activity. Amino acyl tRNA synthetases class I and II represent additional classes of AFEs. They activate amino acids to form charged amino acyl-tRNA. We explored the hypothesis that the activated esters could be hijacked by nucleoside sulfamates to form stable inhibitory conjugates.

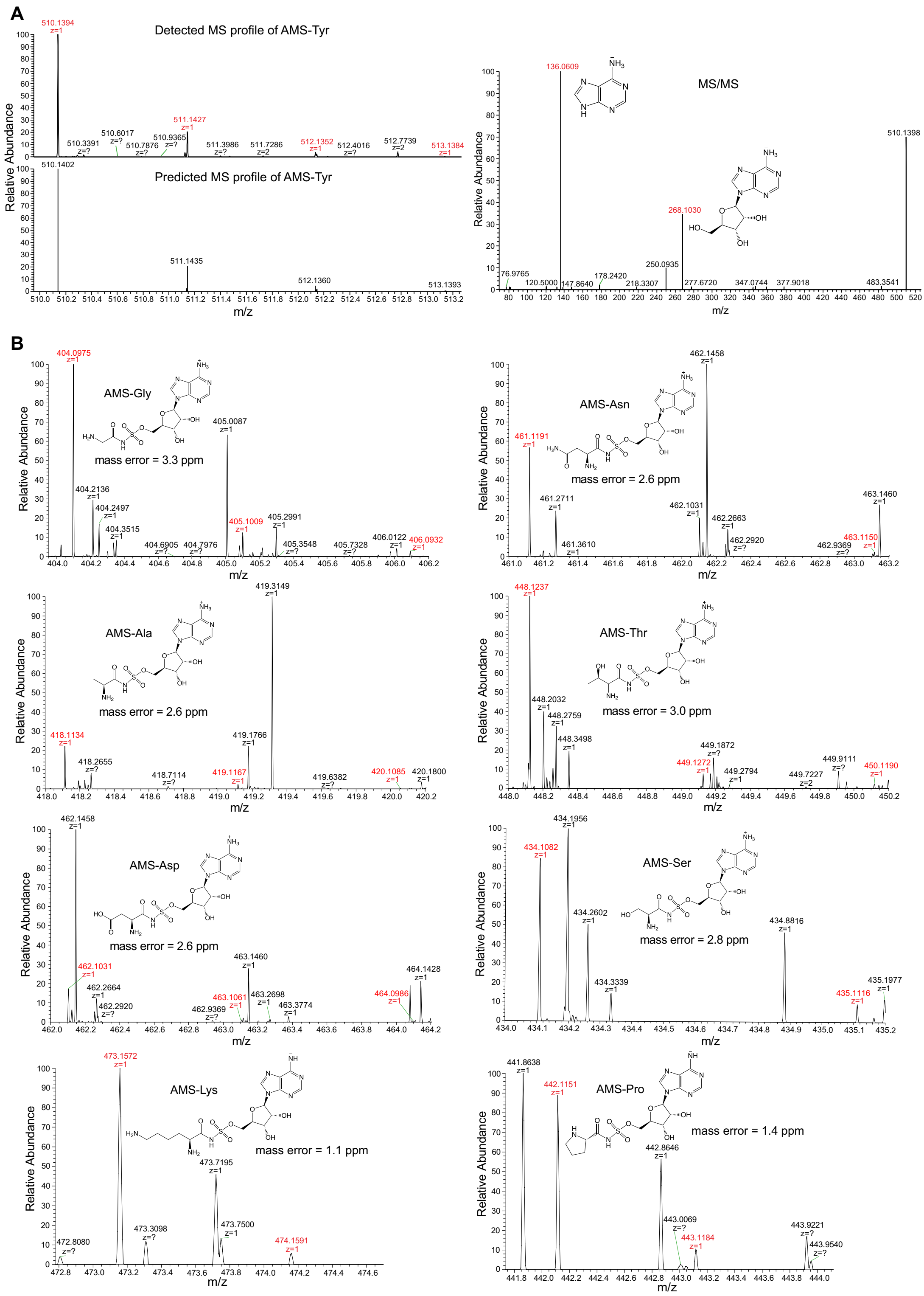

Supplementary Figure 2. Identification of adenosine 5'-sulfamate amino acid conjugates in *P. falciparum*. *P. falciparum*-infected red blood cells (RBCs) were treated with 10 μM AMS for 3 h. Extracts were subjected to high resolution LCMS analysis to search for amino acid-AMS conjugates. Protonated precursor and fragment ions of the expected adduct of Tyr-AMS are highlighted in red. (**A**) MS and MS/MS analysis of detected AMS-Tyr adduct. (**B**) MS analysis of AMS-Gly, AMS-Asn, AMS-Ala, AMS-Thr, AMS-Asp, AMS-Ser, AMS-Lys and AMS-Pro adducts detected (all spectra obtained from LCMS with reversed phase chromatography except for AMS-Lys and AMS-Pro, obtained using HILIC chromatography). The profile is typical of data from three independent experiments.

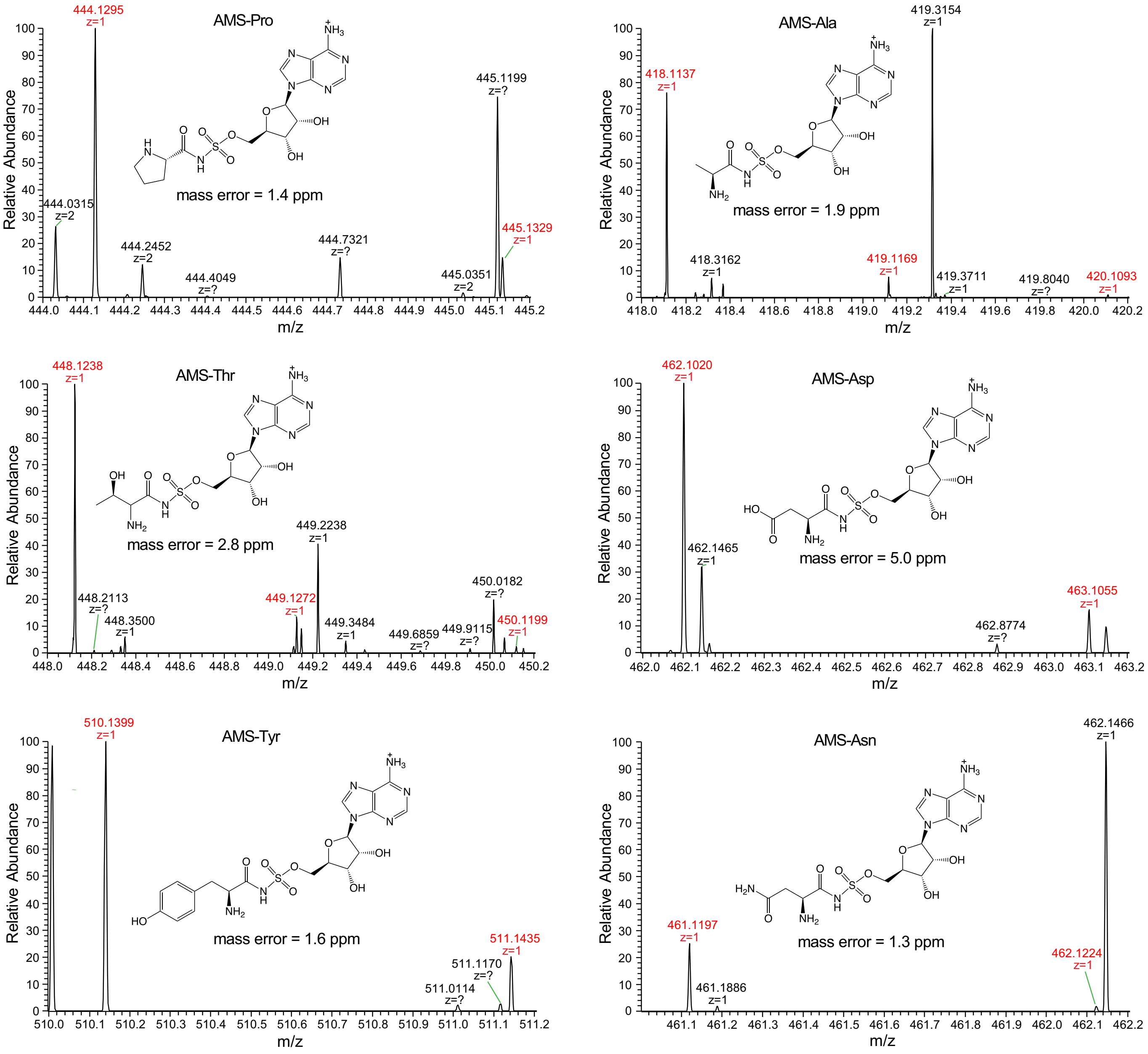

Supplementary Figure 3. Identification of adenosine 5'-sulfamate conjugates in HeLa cells. A culture of HeLa cells was treated with 10 µM AMS in c-DMEM or c-DMEM alone (control) for 2 h. Extracts were subjected to mass spectrometry analysis to search for amino acid-AMS conjugates. The *m/z* values corresponding to the protonated AMS conjugates of Pro, Ala, Thr, Asp, Tyr and Asn were retrieved. Protonated ions of the expected adducts are highlighted in red. The profile is typical of data from three independent experiments.

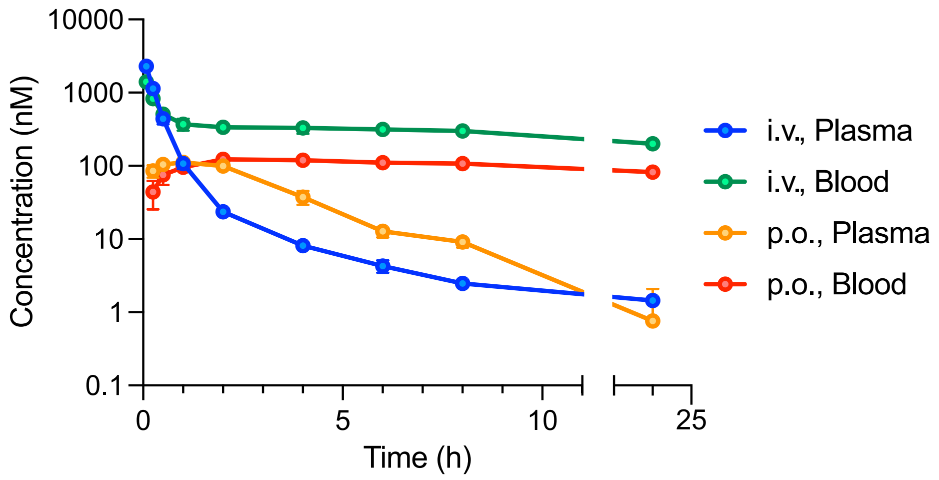

Supplementary Figure 4. Rat pharmacokinetics profiles for ML901. Rats (n = 2) were dosed with ML901 at 1 mg/kg i.v. (green, blue) or 10 mg/kg p.o. (red, orange) and plasma and blood samples were collected for analysis. See Suppl Table 3 for pharmacokinetics values.

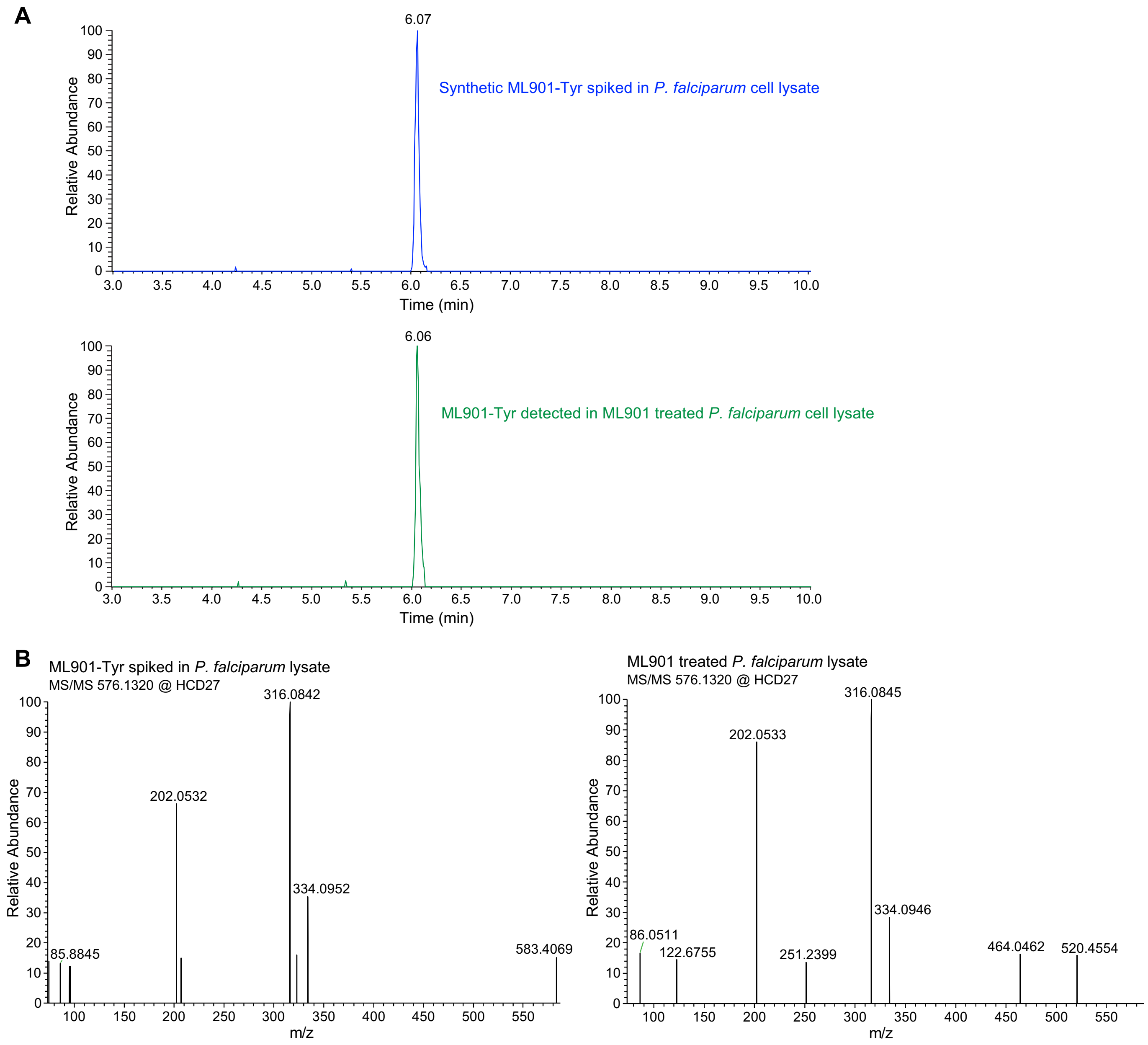

Supplementary Figure 5. Identification of ML901 conjugates in *P. falciparum*. *P. falciparum*-infected RBCs were treated with 3 μM ML901 for 3 h. Extracts were subjected to LCMS to search for amino acid-ML901 conjugates. (**A**) Extracted ion chromatogram (XIC) peaks for the synthetic ML901-Tyr (0.1 μM) spiked into a lysate of *P. falciparum* and the adduct detected from ML901-treated parasites. (**B**) MS/MS analysis of the spiked ML901-Tyr and the adduct made by *P. falciparum*. Profiles are typical of data from 3 independent experiments.

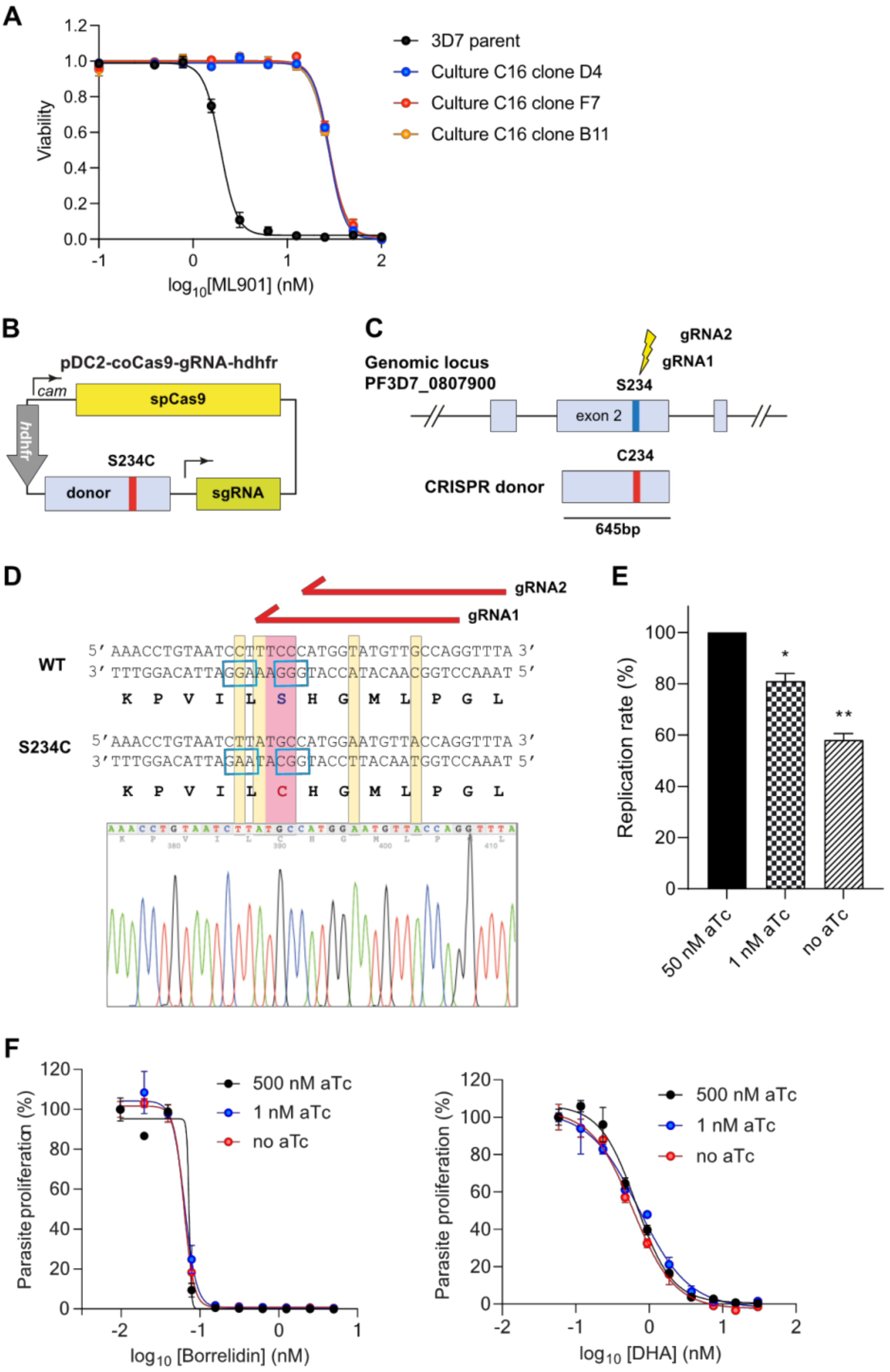

Supplementary Figure 6. *In vitro* evolution of resistance to ML901 and characterization of a conditional *Pf*YRS knockdown line. (A) IC_{50_72h} determination for the 3D7 parent and clones of a culture

(C16) selected at 16 nM ML901. IC_{50} (3D7) = 1.9 ± 0.1 nM, IC_{50} (clones D4, F7, B11) = 28 ± 1 nM. Data represent the average of three independent assays and error bars represent SEM. (**B**-**D**) Generation of a CRISPR-edited Ser234Cys mutant transfectant (Dd2 background). (**B**) Plasmid map illustrating the all-in-one Cas9-gRNA-donor plasmid used for editing the S234C mutation in *Pf*YRS. (**C**) Genomic locus of the three-exon *Pf*YRS gene (PF3D7_0807900). The S234C mutation was edited using a 645 bp donor template that encompasses most of exon 2. (**D**) Sequence of the edited region, indicating the binding site of the two gRNAs and their corresponding PAM motifs (blue boxes, AGG and GGG for gRNA 1 and 2, respectively), the S234C codon (highlighted in red) as well as the silent shield mutations (yellow) that were introduced to prevent gRNA binding. A corresponding chromatogram is shown for clone G12. (**E**) Relative growth rates of an aptamer-regulatable *Pf*YRS line in the absence or presence of aTc at 1 nM (low) or 50 nm (high). Data represent the mean of n = 3 ± SEM. * $p \leq 0.05$ and ** $p \leq 0.005$ by Student's t-test comparing low and no aTc, respectively, to high aTc. (**F**) Sensitivity to Thr-RS inhibitor, borrelidin, and dihydroxyartemisinin (DHA) exposure (72-h) for an aptamer-regulatable *Pf*YRS line, upon addition of aTc, with data normalized to a no drug control. See Supp Table 5 for data values.

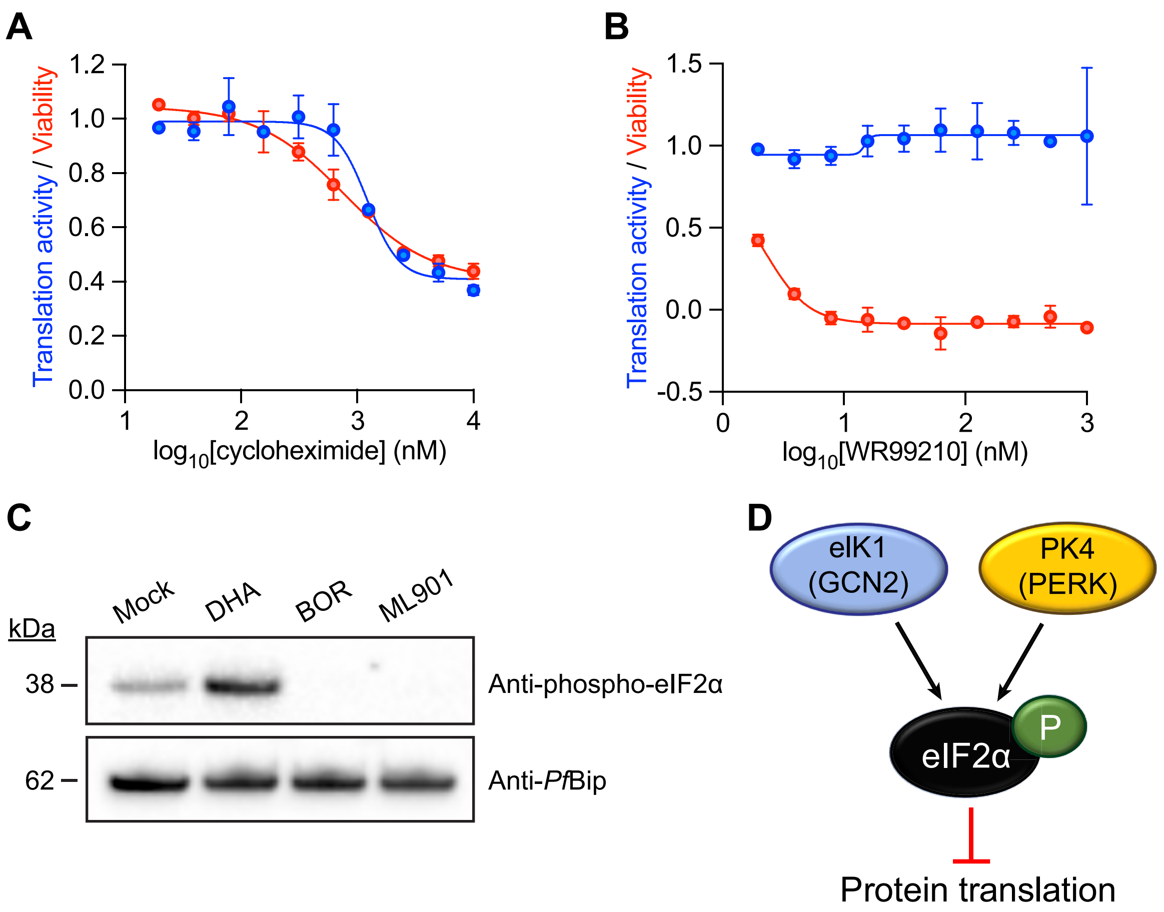

Supplementary Figure 7. ML901 inhibits protein translation. RBCs infected with *P. falciparum* (Cam3.II-rev) were exposed to cycloheximide (**A**) or antifolate WR99210 (**B**) at schizont stage (43-46 h p.i.). For the protein translation assay, cultures were incubated with inhibitor for 1 h prior to assessment of the incorporation of O-propargyl-puromycin (OPP) over the next 2 h. For the viability assay, cultures were exposed to inhibitor for 3 h, then washed and returned to culture. Viability was estimated at the trophozoite stage of the next cycle. For cycloheximide, IC_{50} (Translation) = 2.0 μM, IC_{50} (Viability) = 3.2 μM. For WR99210, IC_{50} (Translation) > 1 μM, IC_{50} (Viability) = 1.9 nM. Data are representative of three independent experiments. Error bars correspond to the range of technical duplicates. (**C**) Schizont stage 3D7_*eIK1* knockout parasites were incubated with the DMSO (Mock), 1 μM DHA, 200 nM borrelidin (BOR) or 200 nM ML901 for 3 h and lysates were subjected to Western blot analysis and probed for phosphorylated-eIF2α. The blot is representative of three independent experiments. (**D**) Diagram illustrating the two pathways that can lead to phosphorylation of eIF2α in Plasmodium. The amino acid starvation pathway (caused by a build-up of uncharged tRNA) is signalled via the eIK1 kinase (the GCN2 equivalent). The ER stress pathway is signalled via the PK4 kinase (PERK equivalent). Deletion of eIK1 ablates eIF2α phosphorylation caused by the borrelidin (BOR) and ML901, consistent with targeting tRNA synthetases. DHA causes ER stress (*15*) and its ability to cause eIF2α phosphorylation is not affected by eIK1 deletion.

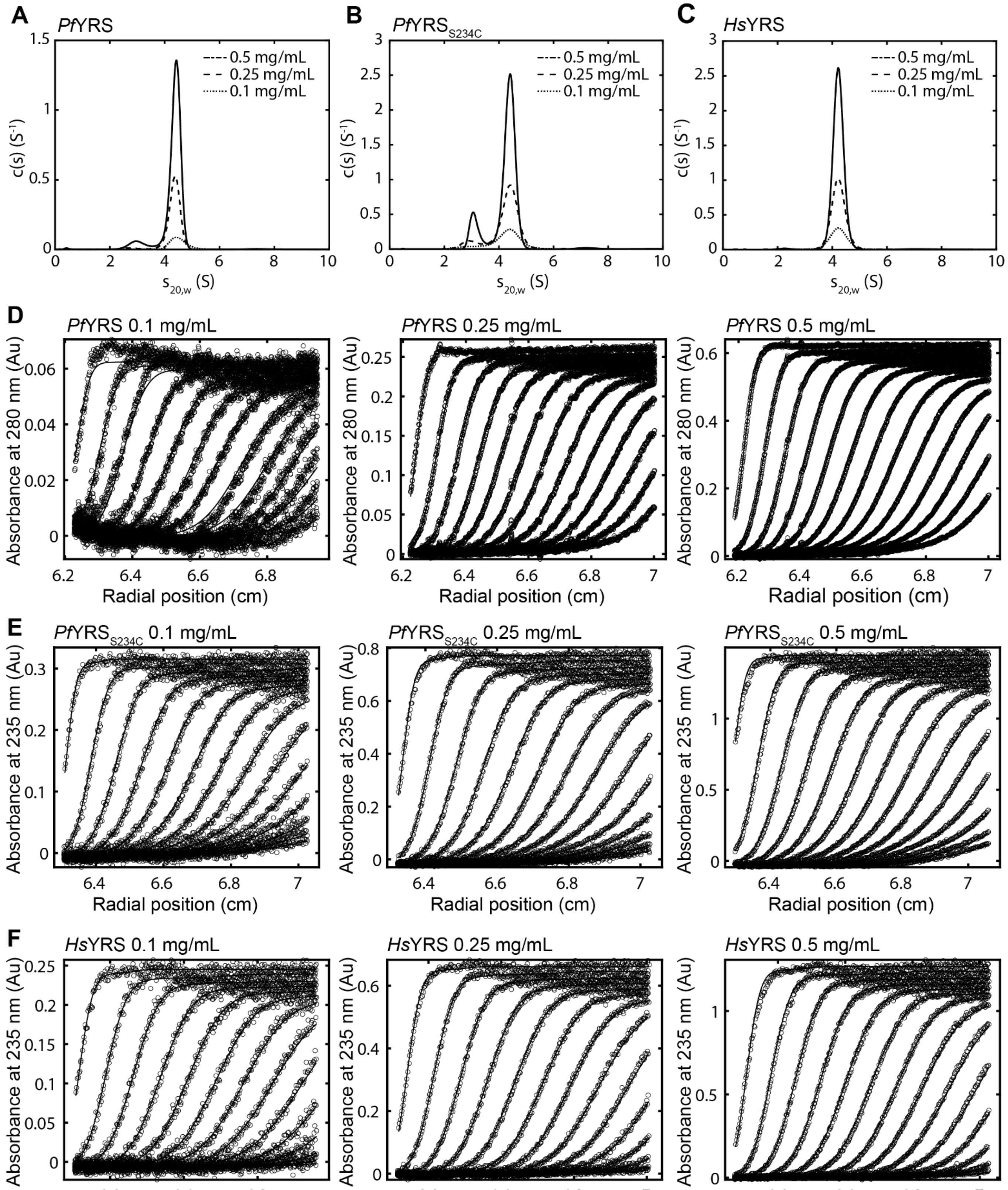

Supplementary Figure 8. **Physical characterization of recombinant *Pf*YRS, *Pf*YRS$_{S234C}$ and *Hs*YRS.** Continuous sedimentation coefficient (c(s)) distributions derived from sedimentation velocity analytical ultracentrifugation for *Pf*YRS (**A**), *Pf*YRS$_{S234C}$ (**B**) and *Hs*YRS (**C**). The measured sedimentation coefficients ($s_{20,w}$) at 0.5 mg/mL are 4.4 S for *Pf*YRS, 4.4S for *Pf*YRS$_{S234C}$ and 4.2S for *Hs*YRS, corresponding to a mass of 85.6 kDa for *Pf*YRS, 84.2 kDa for *Pf*YRS$_{S234C}$ and 82.1 kDa for *Hs*YRS, all

consistent with a dimeric state in solution. (**D**) Raw analytical ultracentrifugation-sedimentation velocity data for *Pf*YRS (collected at three concentrations), overlaid with fits to a continuous sedimentation coefficient (c(s)) model, at 0.5, 0.25 and 0.1 mg/mL. For clarity, every tenth scan is shown. (**E**) Raw analytical ultracentrifugation-sedimentation velocity data for *Pf*YRS$_{S234C}$ (collected at three concentrations), overlaid with fits to a continuous sedimentation coefficient (c(s)) model, at 0.5, 0.25 and 0.1 mg/mL. For clarity, every fourth scan is shown. (**F**) Raw analytical ultracentrifugation-sedimentation velocity data for *Hs*YRS (collected at three concentrations), overlaid with fits to a continuous sedimentation coefficient (c(s)) model, at 0.5, 0.25 and 0.1 mg/mL. For clarity, every fourth scan is shown.

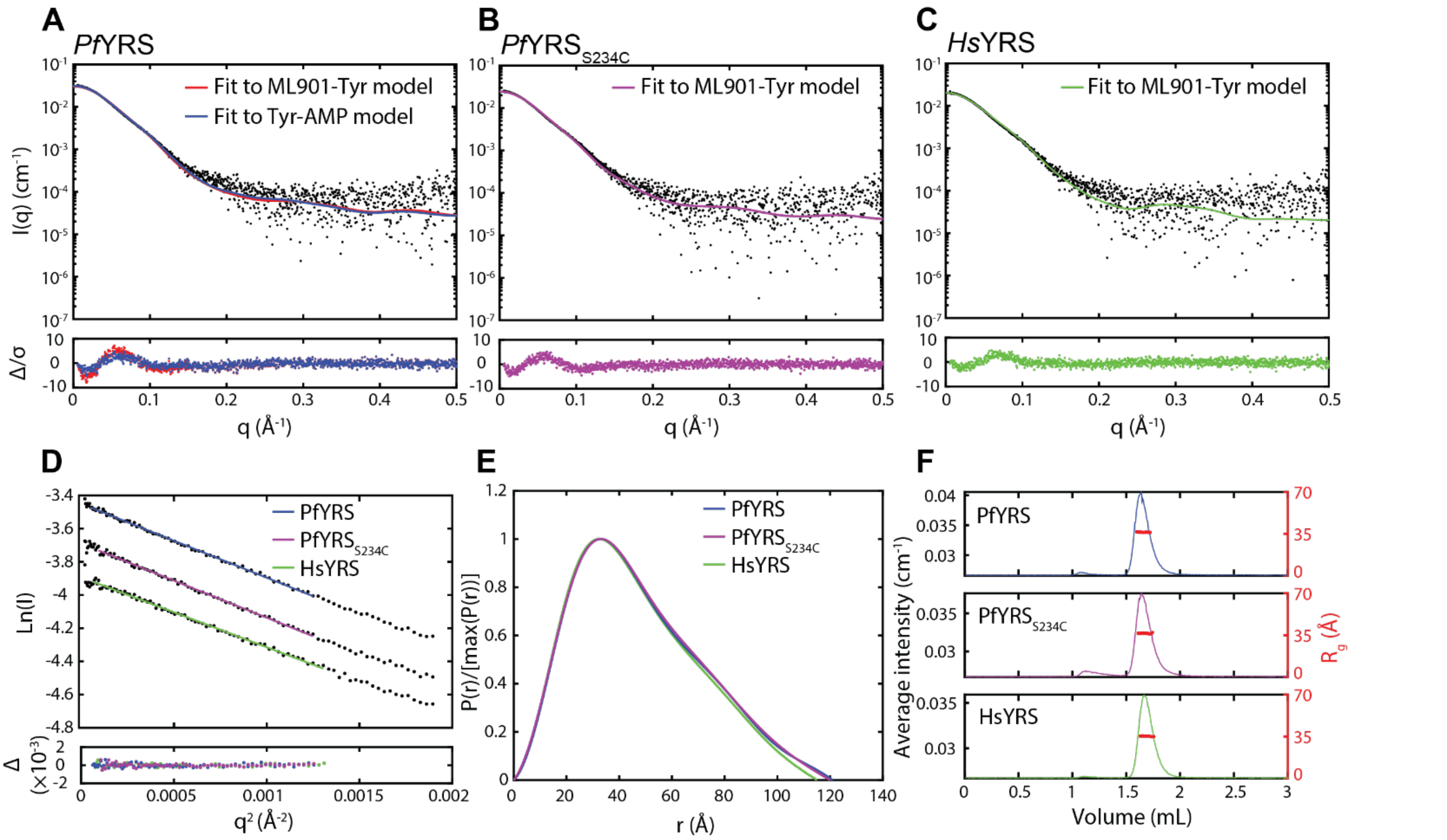

Supplementary Figure 9. **Small angle X-ray scattering (SAXS) analysis of *Pf*YRS, *Pf*YRS$_{S234C}$ and *Hs*YRS.** SAXS data for *Pf*YRS (**A**), *Pf*YRS$_{S234C}$ (**B**) and *Hs*YRS (**C**). CRYSOL fits are shown to the structures indicated. Guinier plots (**D**) and pairwise distance distribution (P(r)) functions (**E**) generated from SAXS data shown in **A**, **B**, and **C**. (**F**) SEC-SAXS chromatograms.

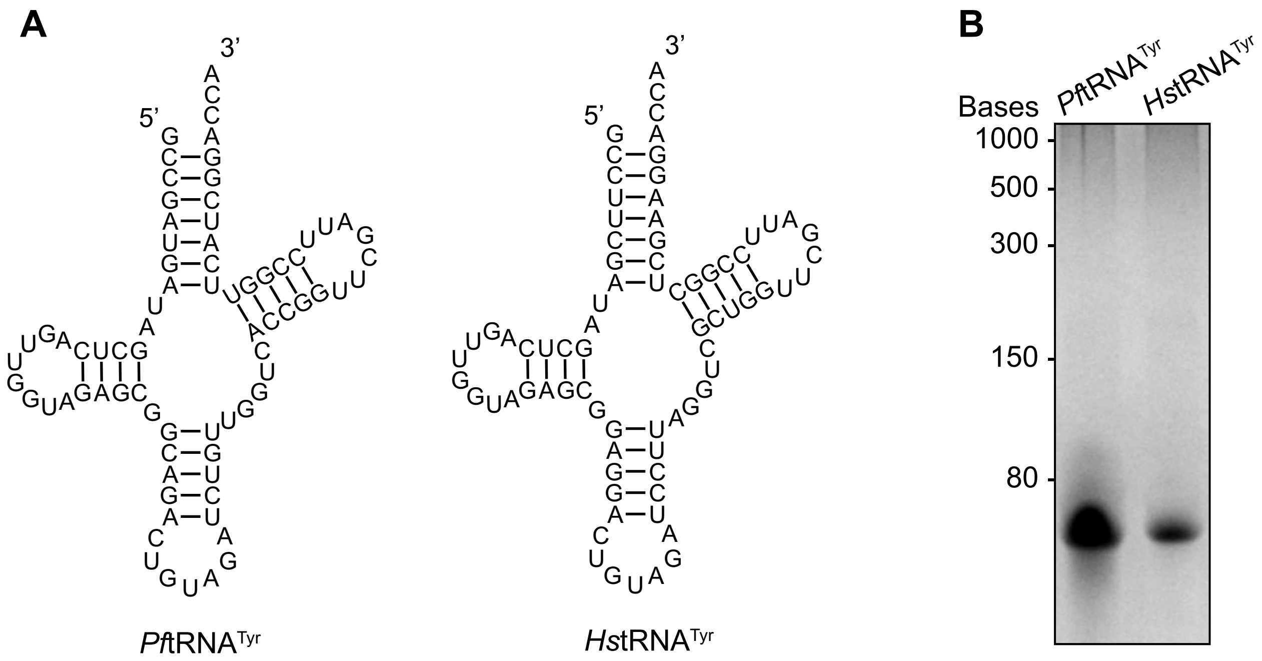

Supplementary Figure 10. ***In vitro* production of *Pf*tRNA^{Tyr} and *Hs*RNA^{Tyr}.** (**A**) Maps of *Pf*tRNA^{Tyr} and *Hs*tRNA^{Tyr}. (**B**) Urea PAGE gels showing the purified *Pf*tRNA^{Tyr} and *Hs*tRNA^{Tyr}.

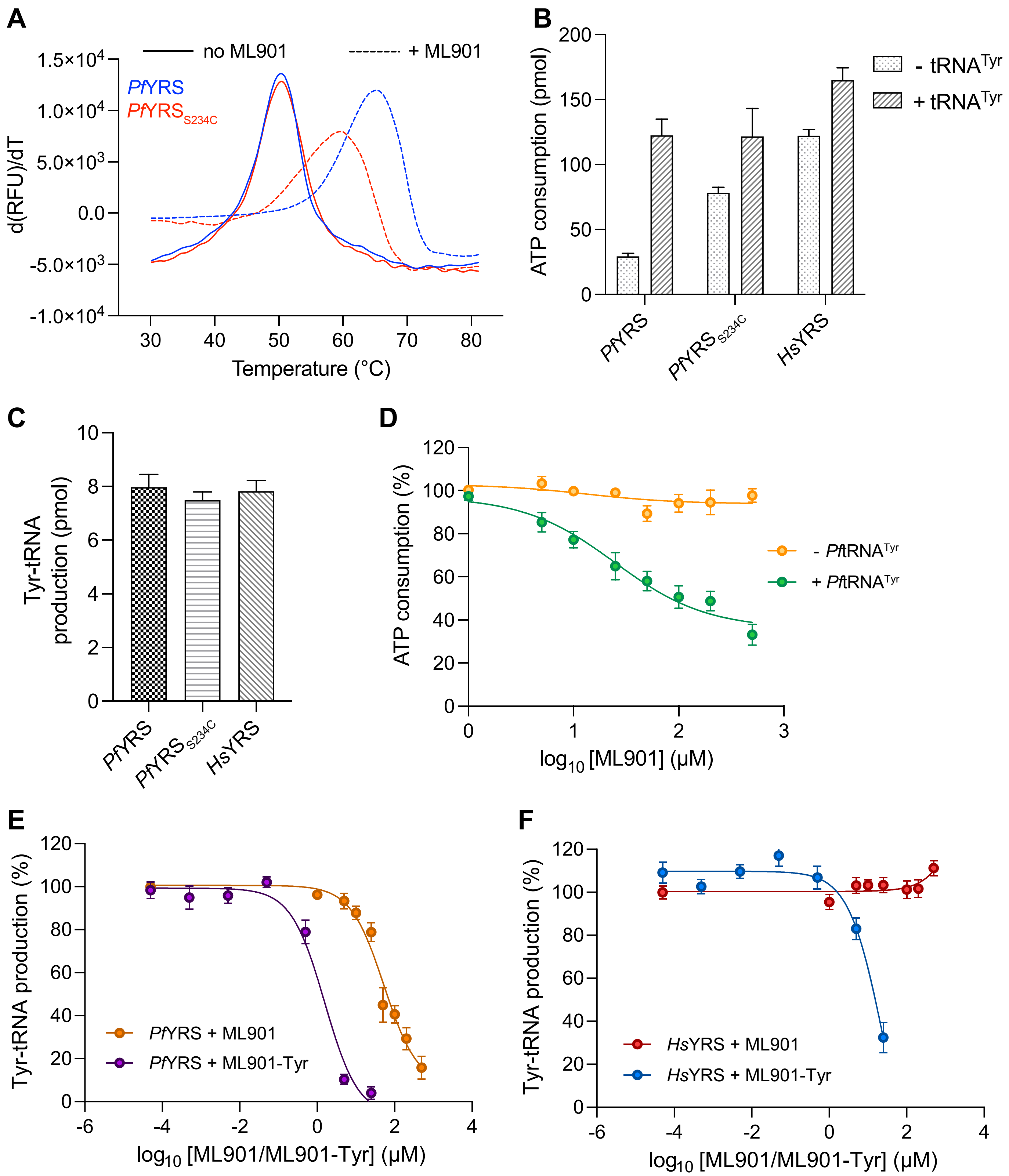

Supplementary Figure 11. **Ligand binding and enzymatic activities of recombinant YRSs.** (**A**) First derivatives of melting curves for *Pf*YRS and *Pf*YRS$_{S234C}$ (2.3 μM) was measured in the apo form or after incubation at 37°C for 3 h with ML901 (50 μM) in the presence of 10 μM ATP, 20 μM tyrosine, 4 μM cognate tRNA^{Tyr}. Data is representative of three independent experiments. (**B**) ATP consumption by wildtype *Pf*YRS, *Pf*YRS$_{S234C}$ and *Hs*YRS in the presence and absence of the cognate tRNA^{Tyr}. ATP consumption in the absence of tRNA^{Tyr} derives from the formation (and release) of AMP-Tyr in the initial

phase of the YRS reaction. Data represent the average of 5-12 independent assays and error bars correspond to SEM. (**C**) Efficiency of tyrosine acylation by *Pf*YRS, *Pf*YRS_{S234C} and *Hs*YRS. Data represent the average of 7-8 independent assays and error bars correspond to SEM. (**D**) Effect of increasing concentrations of ML901 on ATP consumption by wildtype *Pf*YRS in the presence or absence of the cognate $tRNA^{Tyr}$. The reaction concentrations for panels b-d are: YRS (0.2-0.25 μM), ATP (10 μM), tyrosine (100 – 200 μM), cognate $tRNA^{Tyr}$ (24 μM) and pyrophosphatase (1 unit/mL); and incubations were at 37°C for 1 to 3 h. IC_{50} (+*Pf*$tRNA^{Tyr}$) = 25 μM; IC_{50} (-*Pf*$tRNA^{Tyr}$) > 500 μM. Data represent the average of 3-8 independent assays and error bars correspond to SEM. (**E**,**F**) Effects of increasing concentrations of ML901 (alone) and ML901-Tyr on tyrosine acylation of the cognate $tRNA^{Tyr}$ by *Pf*YRS and *Hs*YRS (0.25 μM), at 37°C for 1 h. For *Pf*YRS (**E**), IC_{50} (ML901) = 53 μM; IC_{50} (ML901-Tyr) = 1.6 μM. For *Hs*YRS (**F**), IC_{50} (ML901) > 500 μM; IC_{50} (ML901-Tyr) = 23 μM. Data represent the average of 7-11 independent assays and error bars correspond to SEM.

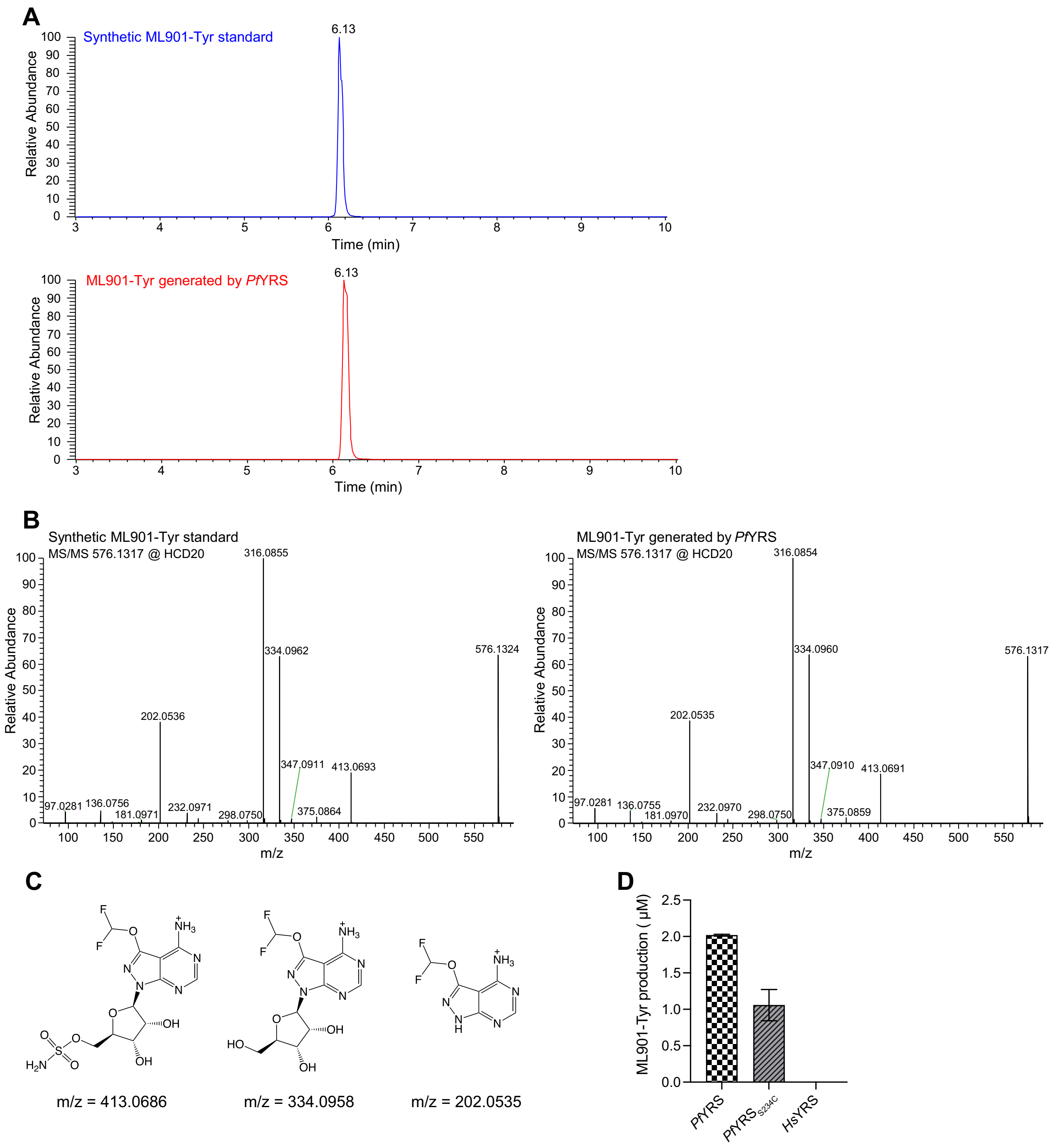

Supplementary Figure 12. Identification of the ML901-Tyr conjugate generated by recombinant YRS. *Pf*YRS, *Pf*YRS$_{S234C}$ and *Hs*YRS (2.3 µM) were incubated with 10 µM ATP, 50 µM ML901, 20 µM tyrosine, 8 µM *Pf*tRNA^{Tyr} for 3 h at 37C. Following denaturation in 4 M urea and precipitation with 1% TFA, the supernatant was subjected to liquid chromatography coupled to mass spectrometry (LCMS) analysis. (**A**) Extracted ion chromatogram (XIC) for protonated synthetic ML901-Tyr (2 µM) and the ML901-Tyr conjugate produced by *Pf*YRS at *m/z* 576.1331. (**B**) MS/MS analysis of synthetic ML901-Tyr and the conjugate made by *Pf*YRS. (**C**) Structures of ML901-Tyr protonated fragment ions. (**D**) The stoichiometry of production of ML901-Tyr by *Pf*YRS, *Pf*YRS$_{S234C}$ and *Hs*YRS was assessed by comparing the XIC peak areas of its precursor ions with an external calibration curve prepared using synthetic ML901-Tyr standards at 0.2 µM, 0.5 µM, 1 µM, 2 µM, 5 µM. Data represent the average of three independent

experiments and error bars correspond to SD. This stands in contrast to Pevonedistat where selectivity for NEDD8 activating enzyme (NAE) versus ubiquitin activating enzyme (UAE) and potency for NAE versus Pevonedistat-resistant NAE mutations is driven primarily by the affinities of the different enzymes for the Pevonedistat-UBL conjugates (*23, 24*).

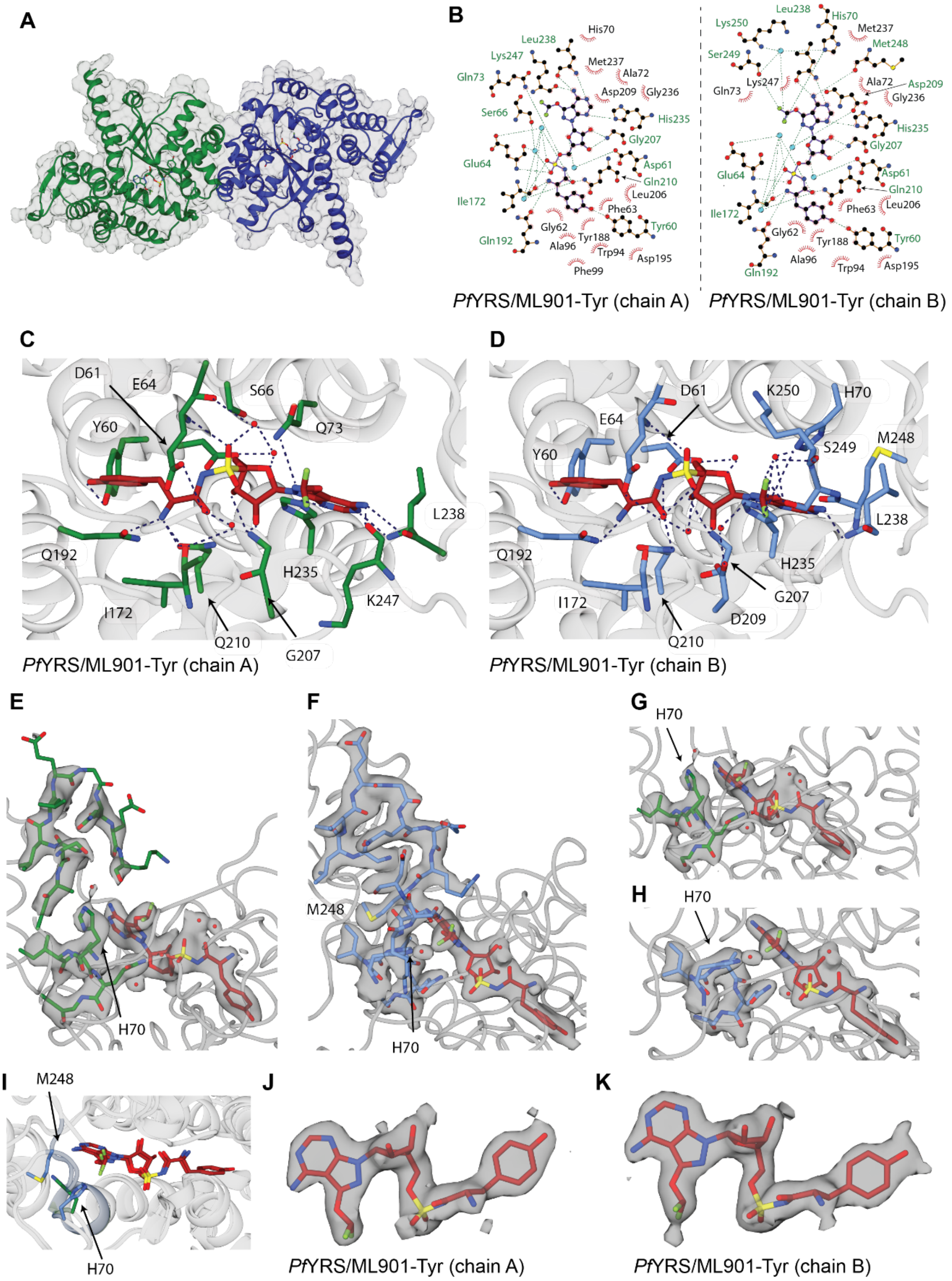

Supplementary Figure 13. Crystal structure of wildtype *Pf*YRS in complex with enzyme-generated ML901-Tyr. (**A**) The structure of the dimeric *PfYRS*/ML901-Tyr showing chain A (green), chain B (blue), and bound ML901-Tyr (red stick representation) looking down the 2-fold axis. (**B**) LigPlot maps of interacting residues for chains A and B. (**C**,**D**) Inhibitor/active site interactions for the A and B chains. (**E**-**H**) $2F_o$-F_c maps contoured at 1 σ (grey surface) showing electron density surrounding the $_{70}$HIAQ$_{73}$ and $_{247}$KMSKS$_{251}$ motifs for chain A (**E**,**G**) and chain B (**F**,**H**). (i) Overlay of chain A and chain B showing the different configurations adopted by His70. (**J**,**K**) $2F_o$-F_c maps contoured at 1 σ (grey surface) showing electron density supporting the position of ML901-Tyr bound to chain A and chain B.

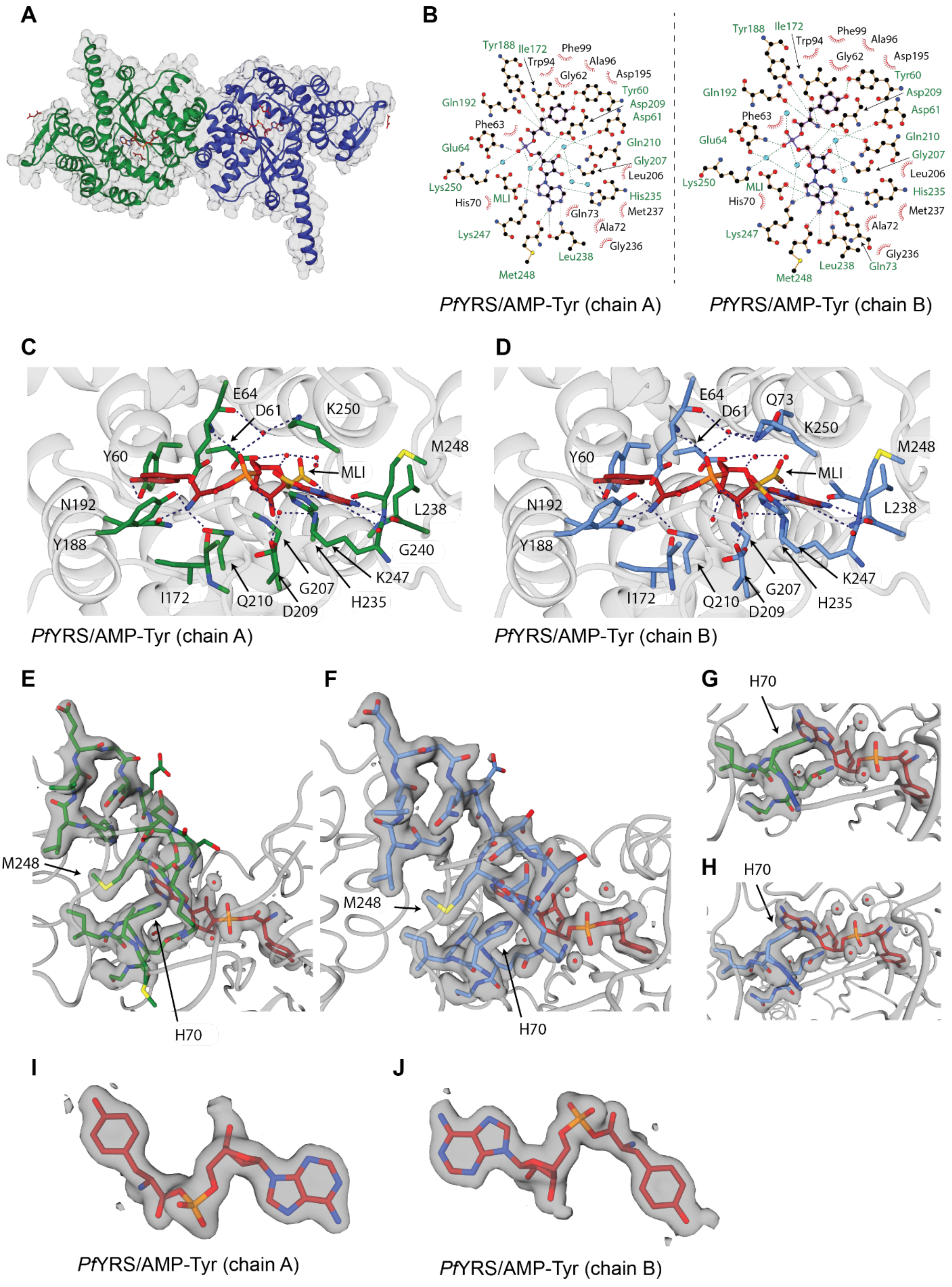

Supplementary Figure 14. Crystal structure of wildtype *Pf*YRS in complex with AMP-Tyr. (**A**) The structure of the dimeric *PfYRS*/AMP-Tyr showing chain A (green), chain B (blue), and bound AMP-Tyr (red stick representation). (**B**) LigPlot maps of interacting residues for chains A and B. (**C**,**D**) Inhibitor/active site interfaces for the chains A and B. (**E-H**) $2F_o$-F_c maps contoured at 1 σ (grey surface) surrounding the $_{70}$HIAQ$_{73}$ and $_{247}$KMSKS$_{251}$ motifs. (**I**,**J**) $2F_o$-F_c maps contoured at 1 σ (grey surface) showing electron density supporting the position of AMP-Tyr bound to chain A and chain B.

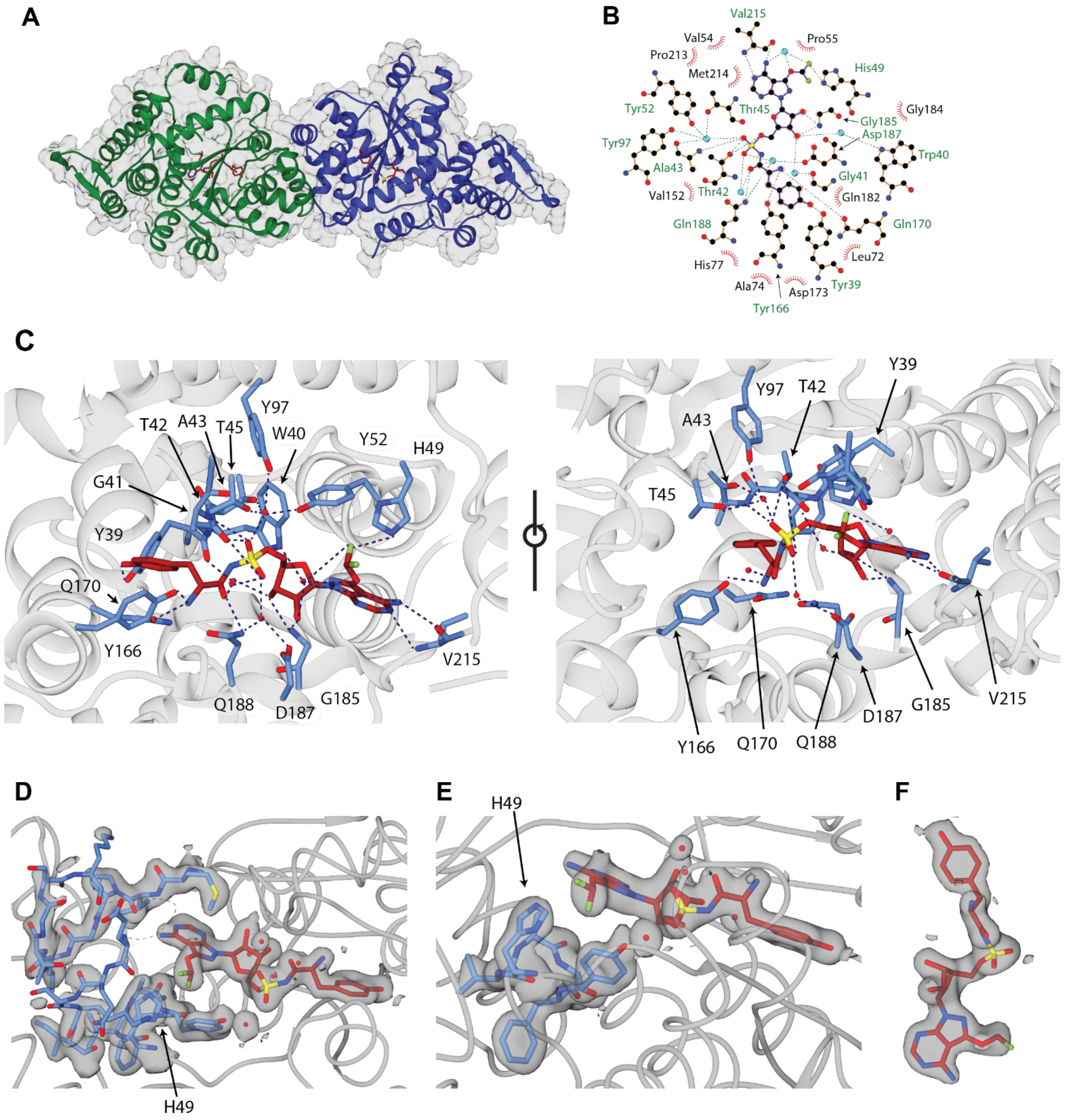

Supplementary Figure 15. Crystal structure of *Hs*YRS in complex with synthetic ML901-Tyr reveals flexibility of the KMSSS loop. (**A**) Ribbon representation of the dimeric *HsYRS*/ML901-Tyr complex (monomers shown in green and blue). The dimer was reconstructed from the crystallographic symmetry, as the asymmetric unit is the *Hs*YRS monomer. (**B**) LigPlot maps of interacting residues. (**C**) Orthogonal views of the inhibitor/active site interactions. (**D**,**E**) $2F_o$-F_c maps contoured at 1 σ (grey surface) surrounding the active site motifs. (**F**) $2F_o$-F_c map contoured at 1 σ (grey surface) showing electron density supporting the position of ML901-Tyr.

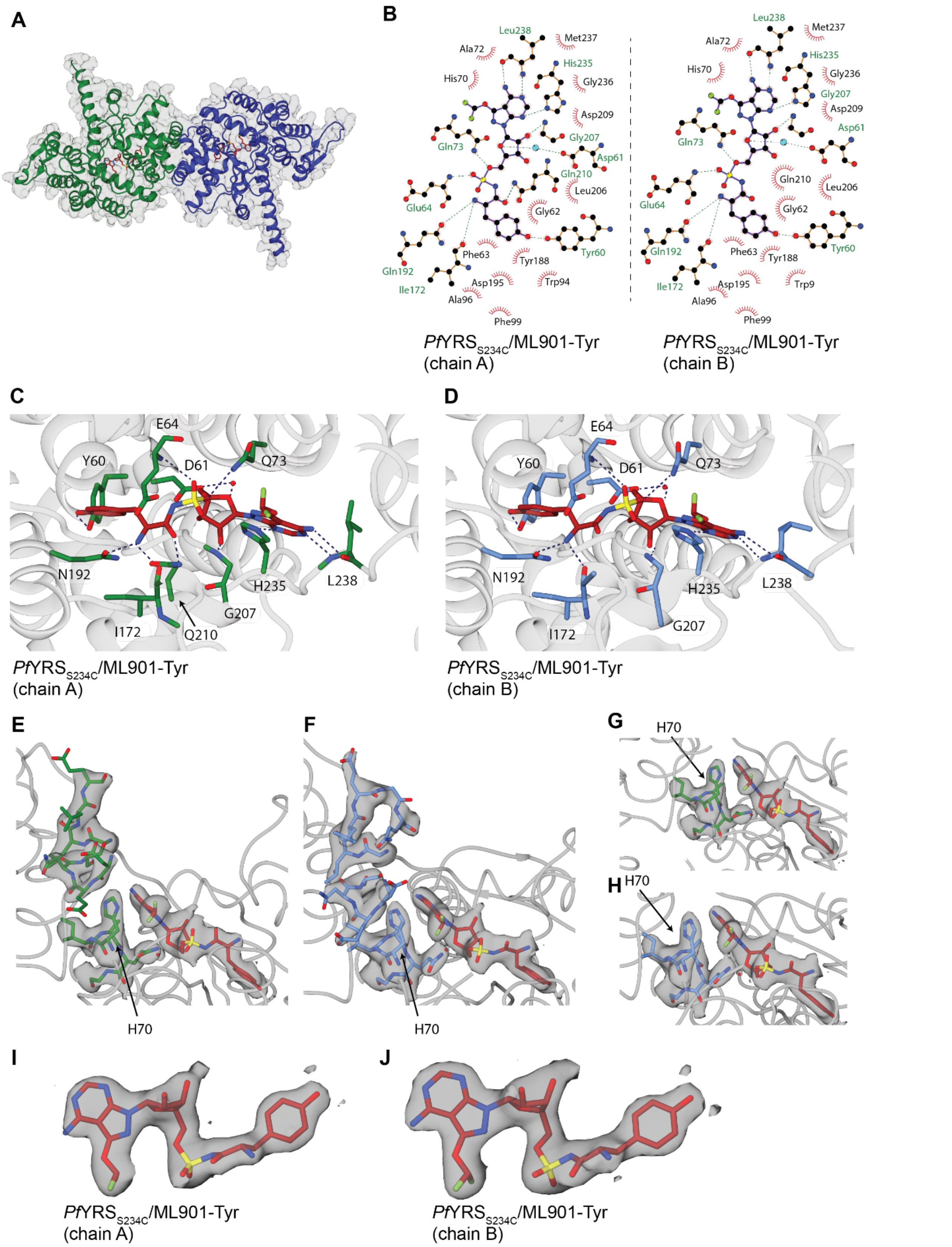

Supplementary Figure 16. Crystal structure of *Pf*YRS$_{S234C}$ in complex with synthetic ML901-Tyr reveals flexibility of the KMSKS loop. (**A**) Ribbon representation of the *Pf*YRS$_{S234C}$ dimer showing the A chain (green) and the B chain (blue). (**B**) LigPlot maps of interacting residues for the A and B chains. (**C**,**D**) Inhibitor/active site interfaces for the A and B chains. (**E-H**) 2F_o-F_c maps contoured at 1 σ (grey surface) surrounding the $_{70}$HIAQ$_{73}$ and $_{247}$KMSKS$_{251}$ motifs. (**I**,**J**) 2F_o-F_c maps contoured at 1 σ (grey surface) showing electron density supporting the position of ML901-Tyr bound to chain A and chain B.

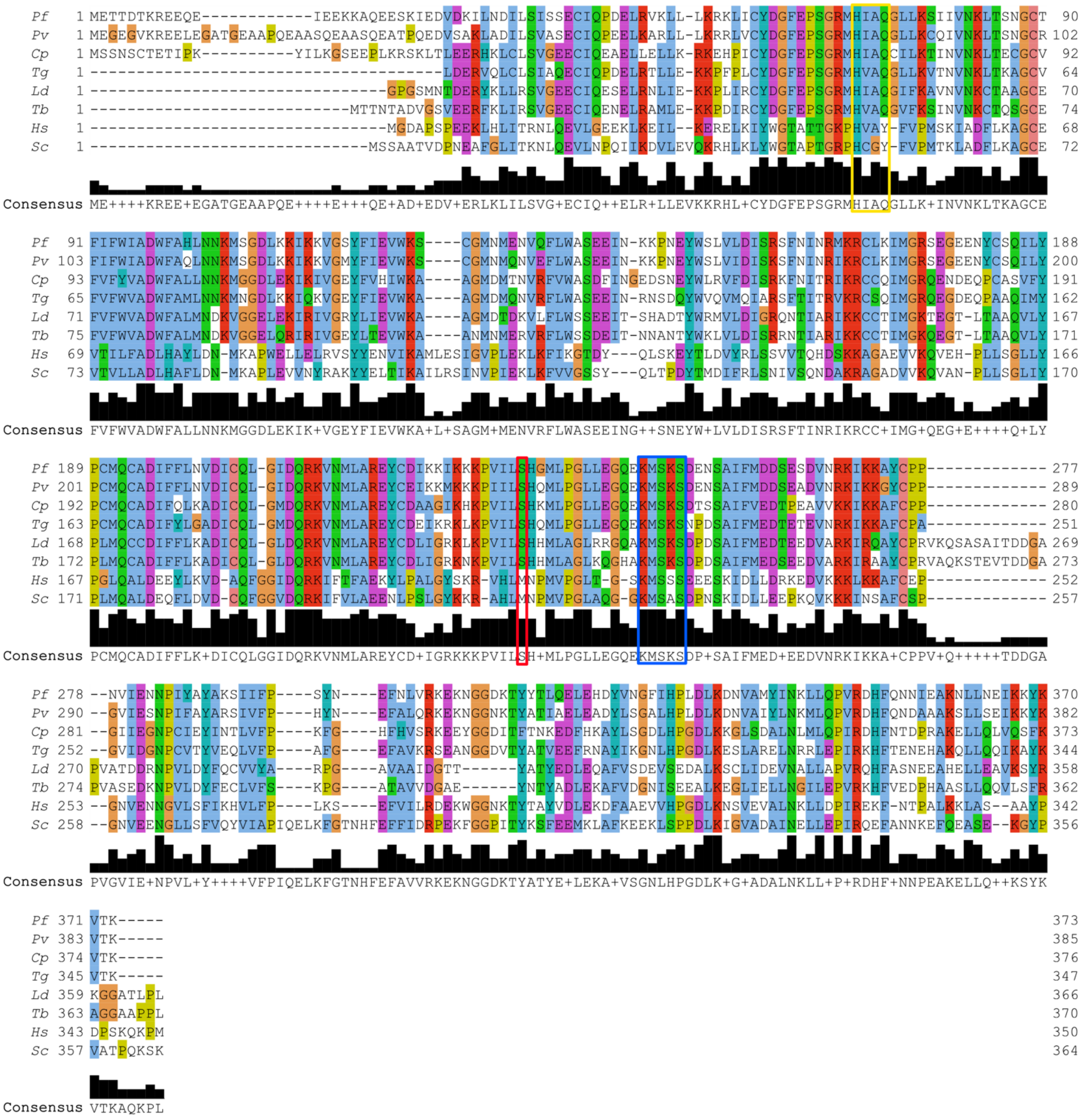

Supplementary Figure 17. **Sequence alignment of *Pf*YRS and other apicomplexan and yeast and human YRSs**. Sequences are shown for *Plasmodium falciparum* (*Pf*), *Plasmodium vivax* (*Pv*), *Cryptosporidium parvum* (*Cp*), *Toxoplasma gondii* (*Tg*), *Leishmania donovani* (*Ld*), *Trypanosoma brucei* (*Tb*), *Homo sapiens* (*Hs*) and *Saccharomyces cerevisiae* (*Sc*). Alignments were generated using Jalview (https://www.jalview.org/). The "HIGH" ($_{70}$HIAQ$_{73}$ in *Pf*YRS; yellow box) and "KMSKS" ($_{247}$KMSKS$_{251}$ in *Pf*YRS; blue box) motifs are indicated. Ser234 (red box) is mutated to Cys234 in the ML901-resistant mutant.

Supplementary Tables.

Table S1. Cytotoxicity of AMS, ML901 and DHA against *P. falciparum* (3D7) and mammalian cell lines. AMS = adenosine 5'-sulfamate. DHA = Dihydroartemisinin. n = Number of biological repeats. Data represent mean ± SEM.

Compound	*Pf*3D7 $IC_{50(72h)}$ (nM)	HepG2 (luminescence) $IC_{50(72h)}$ (nM)	H1650 $IC_{50(72h)}$ (nM)	H460 $IC_{50(72h)}$ (nM)	HCT116 $IC_{50(72h)}$ (nM)
AMS	1.8 ± 0.6 (n = 3)	N/A	140 (n = 1)	25 ± 7 (n = 3)	26 ± 7 (n = 3)
ML901	2.0 ± 0.1 (n = 3)	1,630 ± 590 (n = 4)	10,700 ± 1,430 (n = 6)	4,650 ± 490 (n = 10)	2,280 ± 180 (n = 10)
DHA	2.9 ± 0.2 (n = 6)				

Table S2. Activity of ML901 against drug resistant strains, and extraerythrocytic and transmissible forms. Number of experiments in brackets. Where available, data are presented as mean ± SEM.

**Activity of ML901 against different parasite lines**	
Strain (Sensitivity profile)	IC_{50} value (nM)
7G8 (Decreased sensitivity to chloroquine)	3.9/ 4.0 (2)
Cam3.1 (Decreased sensitivity to artemisinin) (*25*)	3.6/ 3.6 (2)
Dd2 (Decreased sensitivity to chloroquine)	3.5/ 5.1 (2)
Dd2 048 (Decreased sensitivity to phosphatidylinositol 4-kinase (PI4K) inhibitor, MMV048) (*26*)	3.4/3.5 (2)
Dd2 DDD107498 (Decreased sensitivity to translation elongation factor 2 (eEF2) inhibitor, DDD107498) (*27*)	3.5/3.6 (2)
Dd2 DSM265 (Decreased sensitivity to dihydroorotate dehydrogenase inhibitor, DSM265) (*28*)	3.5/3.5 (2)
Dd2 GNF156 (Decreased sensitivity to GNF156 (ganaplacide)) (*29*)	3.2/1.1 (2)
Dd2 ELQ300 (Decreased sensitivity to cytochrome bc1 inhibitor, ELQ300) (*30*)	3.7/3.3 (2)
K1 (Decreased sensitivity to chloroquine)	1.7/ 2.2 (2)
TM90C2B (Decreased sensitivity to atovaquone) (*31*)	3.7/3.5 (2)
NF54 (Sensitive)	1.9/ 2.0 (2)
**Activities of ML901 in transmission and exoerythrocytic stage assays**	
Assay	Effect
Dual gamete formation assay (male) (*32*)	$IC_{50(48h)}$ = 0.13 ± 0.01 µM (n = 9)<br>99% inhibition at 25 µM
Dual gamete formation assay (female) (*32*)	$IC_{50(48h)}$ = 4.7 ± 0.3 µM (n = 9)<br>81% inhibition at 25 µM
P. falciparum NF54 schizont development in primary human hepatocytes (*33*)	$IC_{50(96h)}$ = 13 ± 5 nM (n = 3)

Table S3. Activity in biochemical assays and physicochemical and ADME characterization of selected compounds. ATG7 = autophagy-related protein-7. NAE = NEDD8-activating enzyme. UAE = ubiquitin activating enzyme. SAE = SUMO- activating enzyme. GABARAP = GABAA receptor-associated protein. HTRF = Homogeneous Time-Resolved Fluorescence. Data represent mean $\pm$ SEM. n = Number of independent experiments. ALogP values were calculated using D360 (Certara, New Jersey, USA). Rats (n = 2) were dosed with ML901 at 1 mg/kg i.v. or 10 mg/kg p.o. and plasma and blood samples were collected for analysis (see Suppl Figure 4).

Compound	ATG7 IC_{50} HTRF RH-GABARAP (nM)	NAE IC_{50} HTRF (nM)	UAE IC_{50} HTRF (nM)	SAE IC_{50} HTRF (nM)
ML901	33 ± 3 (n = 57)	>1,000 (n = 5)	>1,000 (n = 5)	>1,000 (n = 5)
AMS	410 ± 20 (n = 90)	0.64 ± 0.11 (n = 9)	62 ± 32 (n = 3)	6.3 ± 1.5 (n = 7)

PK data

Compound	Dose (mg/kg)	CL (L/h/kg)	V_{ss} (L/kg)	p.o. C_{max} (nM)	p.o. $T_{1/2\infty}$ (h)
ML901	1 (iv); 10 (p.o.)	2.39 (p); 0.16 (b)	4.89 (p); 6.35 (b)	113 (p); 130 (b)	3.8 (p); 41 (b)

ADME data

Compound	ALogP	Solubility (µM)	Caco2 P_{app} (x10^{-6} cm/s) (A to B; B to A)
ML901	0.069	>200 (5)	0.70; 0.82

Table S4. Mutations identified in 3D7 parasites selected with ML901 and quality metrics for each sequenced parasite line. Three clones from an ML901-pressured culture were sequenced and analysed to identify potential resistance-conferring variants. Variants with ≥90% alleles mapping to the alternate allele are shown.

Clone name	Chrom	Position	Gene ID	Gene description	Mutation**
B11	3	716676	PF3D7_0317500	Kinesin-5*	Asn1568_Asn1569dup
	8	403556	PF3D7_0807900	Tyrosine-tRNA ligase	Ser234Cys
D4	3	716676	PF3D7_0317500	Kinesin-5*	Asn1568_Asn1569dup
	8	403556	PF3D7_0807900	Tyrosine-tRNA ligase	Ser234Cys
	9	705159	intergenic	NA	n.705159_705160insAATA
	13	1504075	PF3D7_1337300	Exoribonuclease	Leu354Pro
F7	3	716676	PF3D7_0317500	Kinesin-5*	Asn1568_Asn1569dup
	8	403556	PF3D7_0807900	Tyrosine-tRNA ligase	Ser234Cys
	9	705159	intergenic	NA	n.705159_705160insAATA

Clone name	Mean whole genome coverage (x)	Aligned reads	Percent bases covered by 5 or more reads
3D7 Parent	128.3	36,601,919	98.9
B11	116.9	31,716,675	98.7
D4	116.8	32,394,253	98.5
F7	127.5	35,084,867	98.9

* An insertion in the kinesin-5 coding sequence leads to an additional Asn in a run of 19 Asn's. Trinucleotide repeats (predominantly AAT), encoding Asn repeats of varying length, are common in the very AT-rich genome of plasmodium; and are generally considered non-functional. Moreover, this Asn repeat sequence motif is not present in human kinesin 5. The insertion is considered unlikely to be important.

** No mutations were observed in any E1 enzyme, including *P. falciparum* Atg7. We note that *Pf*Atg7 is involved in apicoplast biogenesis and that compounds hitting apicoplast targets exhibit a delayed death phenotype (*34*). Thus, while *Pf*Atg7 is a potential secondary target, the potent first cycle activity is consistent with *Pf*YRS being the primary target.

Table S5. Activity of ML901 and control compounds against CRISPR-edited *Pf*YRS$_{S234C}$ mutant lines and *Pf*YRS knockdown lines. Data represent mean ± SEM from three independent experiments.

CRISPR-edited Ser234Cys mutant lines				
Parasite line	Dd2 parent	Mutant E12	Mutant F5	Mutant G12
IC_{50} (nM)	2.2 ± 0.3	22 ± 1	22 ± 3	20 ± 1
Activity of ML901 against the *Pf*YRS knockdown line (n = 3)				
Condition	high aTc (50 nM)	low aTc (1 nm)	no aTc	Fold change in IC_{50} (high aTc/ no aTc)
IC_{50} (nM) against *Pf*YRS knockdown line	3.9 ± 0.9	0.5 ± 0.1	0.3 ± 0.1	13
IC_{50} (nM) against control line	2.4 ± 0.2	–	2.4 ± 0.2	no change
Activity of borrelidin and DHA against the *Pf*YRS knockdown line (n = 3)				
Compound	high aTc (500 nM)	low aTc (1 nM)	no aTc	Fold change in IC_{50} (high aTc/ no aTc)
IC_{50} (nM) of borrelidin	0.07 ± 0.00	0.07 ± 0.01	0.07 ± 0.01	no change
IC_{50} (nM) of DHA	1.1 ± 0.3	1.6 ± 0.5	1.2 ± 0.4	no change

Table S6: SAXS data collection and analysis.

	*Pf*YRS	*Pf*YRS$_{S234C}$	*Hs*YRS
**SAXS data collection**			
Instrument/source	Australian Synchrotron SAXS/WAXS beamline equipped with Pilatus 2M detector and sheath-flow cell for SEC-SAXS (*35-37*).		
Wavelength (Å)	1.078		
Beam energy (keV)	11.5		
Beam size (μm)	250 × 500		
Sample-to-detector distance (mm)	2590		
q measurement range (Å^{1}) [a]	0.005-0.54		
Absolute scaling method	Comparison with scattering from 1 mm pure water		
Normalization	To transmitted intensity from beamstop counter		
Exposure time	1 s measurements from SEC-SAXS elution		
Sample temperature (K)	293		
**SEC-SAXS parameters**			
Column	Superdex 200 5/150 Increase		
Flow rate (mL/min)	0.4		
Loading concentration (mg/mL)	5		
Injection volume (μL)	50		
Solvent	20 mM Tris, 150 mM NaCl, 5 mM TCEP pH 7.4		
**Software employed**			
SAXS data reduction	*I*(*q*) vs *q* using Scatterbrain 2.8.2, SECSAXS solvent subtraction using *CHROMIXS* from *ATSAS* 2.8.3		
Basic analysis (Guinier, *P(r)*, molecular mass)	*PRIMUS* from *ATSAS* 2.8.3, *GNOM* from *ATSAS* 2.83		
Calculation of theoretical intensities	*CRYSOL* from *ATSAS* 2.8.3		
**Structural parameters**			
Mass from V_c (kDa) (expected mass, ratio to expected, in brackets) [b,c]	83.7 (87,0.96)	84.3 (87,0.97)	75.3 (82.2,0.92)
Guinier analysis			
R_g (Å)	36.53 ± 0.10	36.71 ± 0.13	35.54 ± 0.14
I(0) (cm^{1})	0.032 ± 5.4×10^{-5}	0.025 ± 5.5×10^{-5}	0.020 ± 4.9×10^{-5}
qR_g min, max	0.29,1.29	0.40,1.26	0.31,1.29

P(r) analysis d			
R_g (Å)	37.60 ± 0.08	37.62 ± 0.06	36.51 ± 0.07
I(0) (cm^{1})	0.032 ± 4.6×10^{-5}	0.025 ± 3.9×10^{-5}	0.021 ± 3.9×10^{-5}
D_{max} (Å)	121	120	115
Porod volume (Å^3)	125507	127194	117645
**Atomic modelling**			
CRYSOL (no constant subtraction)			
Structure	PDB 7ROR *Pf*YRS with bound AMP-Tyr	PDB 7ROT *Pf*YRS$_{S234C}$ with bound ML901-Tyr	PDB 7ROU *Hs*YRS with bound ML901-Tyr
χ^2	1.91	1.92	1.71
Calculated R_g (Å)	36.64	36.22	36.28
Structure	PDB 7ROS PfYRS with bound ML901-Tyr		
χ^2	3.77		
Calculated R_g (Å)	35.64		
SASBDB accession codes	SASDMC4	SASDMD4	SASDME4

[a] $q=(4\pi \sin\theta)/\lambda$
[b] Ref(*38*)
[c] 'Expected' mass assumes the protein is dimeric.
[d] Errors from *AUTORG* or *GNOM*, ± standard deviation

Table S7. Estimates of YRS ligand binding constants from thermal stabilization analysis. The T_m values for *Pf*YRS, *Pf*YRS$_{S234C}$ or *Hs*YRS (2.3 µM) were measured in the apo form or after incubation at 37°C for 3 h with 1 mM ATP and 1 mM tyrosine (AMP-Tyr) or with ML901 (50 µM) in the presence of 10 µM ATP, 20 µM tyrosine, 4 µM cognate tRNA^{Tyr} (ML901-Tyr). Data represent the average of three independent experiments, mean ± SD. K_D values are estimated from differential scanning fluorimetry (DSF) analysis based on the empirical correlation of ΔT_m and K_D derived in reference (*39*). However, we note the caveat that for some protein : ligand systems it has been shown that changes in T_m do not directly correlate with changes in binding affinity (*40*).

Enzyme/Ligand	T_m (°C)	ΔT_m (°C)	Apparent K_D* (x10^{-9}) (M)
*Pf*YRS/apo	50.2 ± 0.1	---	---
*Pf*YRS$_{S234C}$ /apo	50.6 ± 0.2	---	---
*Hs*YRS/apo	50.5 ± 0.3	---	---
*Pf*YRS/AMP-Tyr	62.6 ± 0.2	12.4	2.2
*Pf*YRS$_{S234C}$/ AMP-Tyr	57.1 ± 0.3	6.5	24
*Hs*YRS/ AMP-Tyr	53.3 ± 0.2	2.8	116
*Pf*YRS/ ML901-Tyr	65.1 ± 0.1	14.9	0.7
*Pf*YRS$_{S234C}$/ ML901-Tyr	59.7 ± 0.3	9.1	3.9
**Based on data from ref (39). Assumes saturating ligand for AMP-Tyr complexes; Effective ML901-Tyr concentration is based on the measurement by LCMS (Suppl Figure 12D).*			

Table S8. X-ray diffraction data collection and refinement statistics. Values for the highest resolution shell are given in parentheses.

	***Pf*YRS/ AMP-Tyr**	***Pf*YRS/ ML901-Tyr**	***Pf*YRS $_{\mathbf{(S235C)}}$ /ML901-Tyr**	***Hs*YRS/ ML901-Tyr**
**Data collection**				
Space group	$C\,2_1$	$C\,2_1$	$C\,2_1$	$P\,2_1\,2_1\,2$
Wavelength (Å)	0.9537	0.9537	0.9537	0.9537
Number of images	3600	3600	3600	3600
Oscillation range per image (˚)	0.1	0.1	0.1	0.1
Detector	Eiger 16M	Eiger 16M	Eiger 16M	Eiger 16M
Cell dimensions				
a, *b*, *c* (Å)	137.61, 46.60, 141.40	137.29, 46.53, 139.01	138.19, 47.68, 140.63	75.62, 162.19, 35.23
α, β, γ (˚)	90, 93.81, 90	90, 93.77, 90	90, 94.46, 90	90, 90, 90
Resolution (Å)	47.65-1.80 (1.83-1.80)	47.21-2.15 (2.22-2.15)	47.33-2.20 (2.27-2.20)	43.98-1.70 (1.73-1.70)
$R_{\mathrm{sym}}^{\dagger}$	0.058 (1.953)	0.124 (1.711)	0.097 (1.020)	0.081 (2.795)
$R_{\mathrm{meas}}^{\S}$	0.069 (2.298)	0.146 (2.003)	0.115 (1.196)	0.087 (3.024)
$R_{\mathrm{pim}}^{\ddagger}$	0.036 (0.862)	0.076 (1.036)	0.061 (0.621)	0.033 (1.147)
$CC_{1/2}$	0.999 (0.492)	0.997 (0.655)	0.998 (0.854)	0.999 (0.467)
$I/\sigma(I)$	13.3 (0.9)	8.4 (1.2)	8.5 (1.6)	13.6 (1.0)
Total observations	576537 (32142)	332949 (30043)	318785 (29255)	647558 (33995)
Unique reflections	83538 (4539)	48258 (4197)	46975 (4078)	48890 (2547)
Completeness (%)	100.0 (100.0)	99.9 (100.0)	99.9 (99.9)	100.0 (100.0)
Multiplicity	6.9 (7.1)	6.9 (7.2)	6.8 (7.2)	7.0 (6.9)
Wilson *B* factor ($Å^2$)	35.6	40.2	43.1	31.794
**Refinement**				
Resolution (Å)	47.65-1.80 (1.86-1.80)	46.24-2.15 (2.23-2.15)	46.74-2.20 (2.28-2.20)	43.98-1.7 (1.76-1.70)
Reflections used in refinement	83482 (8346)	48229 (4774)	46893 (4662)	48816 (4828)
R_{free} reflections	4085 (383)	2336 (228)	2401 (261)	2395 (231)
R_{work}	0.1801 (0.3309)	0.2033 (0.3206)	0.2061 (0.3180)	0.1776 (0.3268)
R_{free}	0.2172 (0.3714)	0.2317 (0.3779)	0.2381 (0.3268)	0.2077 (0.3612)
Protein molecules in asymmetric unit	2	2	2	1
Total nonhydrogen atoms	6324	5927	5867	3060
Protein	5891	5719	5707	2690
Ligand/ion	102	82	81	74
Solvent	331	126	79	296
Mean B *factor ($Å^2$)*	51.16	58.44	65.00	45.94
Protein	51.12	58.71	65.34	44.84
Ligand/ion	43.88	44.79	49.99	64.05
Solvent	54.05	55.17	56.13	51.39
RMS deviations				

Bond lengths (Å) (outliers > 4σ)	0.014 (1)	0.002 (0)	0.004 (0)	0.010 (2)
Bond angles (°) (outliers > 4σ)	1.257 (4)	0.462 (0)	0.581 (0)	1.087 (2)
Rotamer outliers (%)	2.41	1.24	2.33	3.40
Clashscore	6.28	4.31	3.89	3.62
Cβ outliers	0	0	0	7
Molprobity score	1.66	1.37	1.63	1.77
Ramachandran Plot				
Favored (%)	97.90	97.56	96.97	96.68
Allowed (%)	1.82	2.01	2.74	2.72
Outliers (%)	0.28	0.43	0.29	0.60
PDB code	7ROR	7ROS	7ROT	7ROU

† $R_{sym} = \sum_{hkl}\sum_i |I_i(hkl) - \langle I(hkl)\rangle| / \sum_{hkl}\sum_i I_i(hkl)$

§ $R_{meas} = \sum_{hkl} [N/(N-1)]^{1/2} \sum_i |I_i(hkl) - \langle I(hkl)\rangle| / \sum_{hkl}\sum_i I_i(hkl)$

‡ $R_{pim} = \sum_{hkl} [1/(N-1)]^{1/2} \sum_i |I_i(hkl) - \langle I(hkl)\rangle| / \sum_{hkl}\sum_i I_i(hkl)$

$CC_{1/2}$ = Pearson correlation coefficient between independently merged half data sets.

Supplementary Methods

Chemistry Methods

All solvents and chemicals were used as purchased without further purification unless noted.

ML901 and AMS synthesis and characterization

((2R,3S,4R,5R)-5-[4-amino-3-(difluoromethoxy)-1H-pyrazolo[3,4-d]pyrimidin-1-yl]-3,4-dihydroxytetrahydrofuran-2-yl)methyl sulfamate (ML901) was synthesized and characterized as below, see also (*11*). Adenosine 5'-sulfamate (AMS) was generated as previously described (*41*).

Scheme 1. Synthesis of ML901

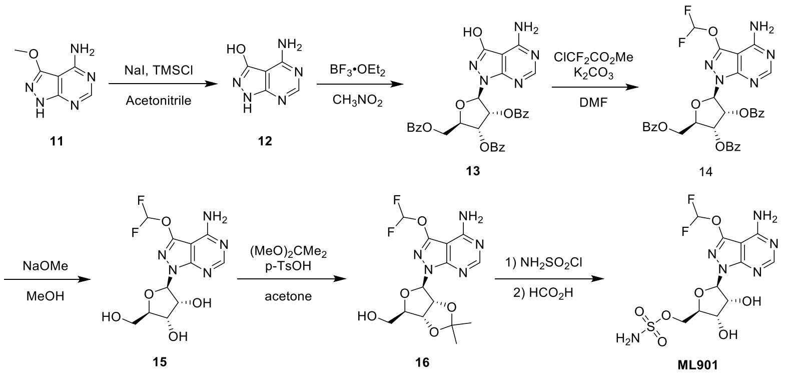

4-Amino-1H-pyrazolo[3,4-d]pyrimidin-3-ol (12).

To a mixture of 3-methoxy-1H-pyrazolo[3,4-d]pyrimidin-4-amine (**11**, 1.00 g, 6.05 mmol) in acetonitrile (170 mL), was added chlorotrimethylsilane (1.15 mL, 9.08 mmol) and sodium iodide (1.36 g, 9.08 mmol). The reaction was heated at 90 °C for 18 h. Additional portions of chlorotrimethylsilane (1.15 mL, 9.08 mmol) and sodium iodide (1.36 g, 9.08 mmol) were added and heating was continued for 3 days. After this time no acetonitrile remained. Approximately 75 mL acetonitrile was added and the solids were isolated by filtration, washed with water and hexane, coevaporated with toluene and dried under vacuum to afford **12** (0.430 g, 47% yield) as a beige solid. LC-MS (*m/z*): 152.0 $[M + H]^+$. ^{1}H NMR (400 MHz, DMSO-d_6): δ 11.38 (br s, 2H), 8.01 (s, 1H), 7.62 (s, 1H), 6.82 (s, 1H).

(2R,3R,4R,5R)-2-(4-amino-3-hydroxy-1H-pyrazolo[3,4-d]pyrimidin-1-yl)-5-[(benzoyloxy)methyl]tetrahydrofuran-3,4-diyl dibenzoate (13).

4-amino-1H-pyrazolo[3,4-d]pyrimidin-3-ol (**12**, 0.43 g, 2.85 mmol), and β-D-ribofuranose 1-acetate 2,3,5-tribenzoate (1.87 g, 3.70 mmol) were combined in a 100 mL, 3 neck flask fitted with a stirbar and reflux condenser. Nitromethane (28 mL) was added to the flask by syringe. The resulting suspension was warmed to reflux in an oil bath. Boron trifluoride etherate (0.631 mL, 4.98 mmol) was then added dropwise to the heated mixture by syringe. The reaction mixture turned homogeneous during completion of BF3 addition. Upon completion of addition, heating was continued 30 min. The dark brown mixture was cooled to rt, poured into ethyl acetate and washed with half-saturated aqueous sodium bicarbonate solution. The mixture was filtered and then the phases were separated. The aqueous phase was extracted with additional ethyl

acetate. The extracts were combined, washed with water then brine, dried over sodium sulfate, filtered and concentrated under reduced pressure. The crude product was purified by silica gel chromatography (dichloromethane to 90/10 dichloromethane/methanol gradient) to afford **13** (0.396 g, 23% yield) as an off white solid. LC-MS (*m/z*): 596.5 $[M + H]^+$. ^{1}H NMR (400 MHz, DMSO-d_6) δ 11.77 (s, 1H), 8.13 (s, 1H), 8.00-7.91 (m, 4H), 7.90-7.86 (m, 2H), 7.70-7.60 (m, 3H), 7.55-7.39 (m, 6H), 6.54 (d, J=2.3 Hz, 1H), 6.20-6.14 (m, 1H), 6.14-6.08 (m, 1H), 4.83-4.77 (m, 1H), 4.56 (ddd, J=16.9, 12.0, 4.5 Hz, 2H).

(2R,3R,4R,5R)-2-[4-amino-3-(difluoromethoxy)-1H-pyrazolo[3,4-d]pyrimidin-1-yl]-5-[(benzoyloxy)methyl]tetrahydrofuran-3,4-diyl dibenzoate (14).

To a solution of (2R,3R,4R,5R)-2-(4-amino-3-hydroxy-1H-pyrazolo[3,4-d]pyrimidin-1-yl)-5-[(benzoyloxy)methyl]tetrahydrofuran-3,4-diyl dibenzoate (**13**, 0.100 g, 0.168 mmol) in DMF (2 mL), was added potassium carbonate (0.070 g, 0.504 mmol) and methyl chlorodifluoroacetate (0.053 mL, 0.504 mmol). The reaction was stirred at room temperature for 90 min, then heated at 80 °C for 30 min. The reaction was cooled to room temperature, diluted with ethyl acetate and neutralized by the addition of 1N HCl. The mixture was extracted with ethyl acetate twice, and the combined organic phases were washed with water then brine, dried over sodium sulfate, filtered and concentrated under reduced pressure. The crude product was purified by silica gel chromatography (dichloromethane to 95/5 dichloromethane/methanol gradient) to afford **14** (0.087 g, 80% yield) as a yellow solid. LC-MS (*m/z*): 646.5 $[M + H]^+$. ^{1}H NMR (400 MHz, $CDCl_3$): δ 8.36 (s, 1H), 8.12-8.07 (m, 2H), 8.02 (s, 1H), 7.99-7.94 (m, 4H), 7.62-7.51 (m, 3H), 7.48-7.34 (m, 5H), 6.96 (t, J = 71.6 Hz, 1H), 6.77 (d, J = 4.4 Hz, 1H), 6.39-6.35 (m, 1H), 6.16 (t, J = 5.2 Hz, 1H), 4.84-4.72 (m, 2H), 4.65-4.53 (m, 1H).

(2R,3R,4S,5R)-2-[4-amino-3-(difluoromethoxy)-1H-pyrazolo[3,4-d]pyrimidin-1-yl]-5-(hydroxymethyl)tetrahydrofuran-3,4-diol (15).

(2R,3R,4R,5R)-2-[4-amino-3-(difluoromethoxy)-1H-pyrazolo[3,4-d]pyrimidin-1-yl]-5-[(benzoyloxy)methyl]tetrahydrofuran-3,4-diyl dibenzoate (**14**, 0.087 g, 0.135 mmol) was dissolved in methanol (2 mL). To this solution was added a solution of sodium methoxide (0.5 M in MeOH, 0.270 mL, 0.135 mmol). After stirring overnight at room temperature, the reaction mixture was concentrated under reduced pressure and purified by silica gel chromatography (dichloromethane to 90/10 dichloromethane/methanol gradient) to afford **15** (0.022 g, 49% yield) as a white solid. LC-MS (*m/z*): 334.2 $[M + H]^+$. ^{1}H NMR (400 MHz, MeOD): δ 8.19 (s, 1H), 7.36 (t, J = 71.8 Hz, 1H), 6.19 (d, J = 4.2 Hz, 1H), 4.68-4.65 (m, 1H), 4.42 (t, J = 5.0 Hz, 1H), 4.09-4.04 (m, 1H), 3.78 (dd, J = 12.2, 3.5 Hz, 1H), 3.66 (dd, J = 12.2, 5.1 Hz, 1H).

{(3aR,4R,6R,6aR)-6-[4-amino-3-(difluoromethoxy)-1H-pyrazolo[3,4-d]pyrimidin-1-yl]-2,2-dimethyltetrahydrofuro[3,4-d][1,3]dioxol-4-yl}methanol (16).

(2R,3R,4S,5R)-2-[4-amino-3-(difluoromethoxy)-1H-pyrazolo[3,4-d]pyrimidin-1-yl]-5-(hydroxymethyl)tetrahydrofuran-3,4-diol (**15**, 0.028 g, 0.084 mmol), acetone (1.561 mL), 2,2-dimethoxypropane (0.155 mL, 1.260 mmol) and p-toluenesulfonic acid monohydrate (0.032 g, 0.168 mmol) were combined in a flask and stirred overnight at room temperature. The reaction mixture was concentrated under reduced pressure and partitioned between saturated aqueous sodium bicarbonate solution and ethyl acetate. The aqueous phase was extracted with additional ethyl acetate and the combined organic phases were washed with water then brine, dried over sodium sulfate, filtered and concentrated under reduced pressure. The crude product was purified by silica gel chromatography (dichloromethane to 90/10 dichloromethane/methanol gradient) to afford **16** (0.016 g, 51% yield) as a white solid. LC-MS (*m/z*): 374.3 $[M + H]^+$. ^{1}H NMR (400 MHz, $CDCl_3$): δ 8.27 (s, 1H), 7.15 (t, J = 71.6 Hz, 1H), 6.32 (d, J = 3.5 Hz, 1H), 5.21 (dd, J = 5.9, 3.5 Hz, 1H), 5.01 (dd, J = 6.0, 1.4 Hz, 1H), 4.81 (s, 1H), 4.51-4.46 (m, 1H), 3.88 (dd, J = 12.6, 2.2 Hz, 1H), 3.81-3.73 (m, 1H), 1.63 (s, 3H), 1.38 (s, 3H).

{(2R,3S,4R,5R)-5-[4-amino-3-(difluoromethoxy)-1H-pyrazolo[3,4-d]pyrimidin-1-yl]-3,4-dihydroxytetrahydrofuran-2-yl}methyl sulfamate (ML901).

{(3aR,4R,6R,6aR)-6-[4-amino-3-(difluoromethoxy)-1H-pyrazolo[3,4-d]pyrimidin-1-yl]-2,2-dimethyltetrahydrofuro[3,4-d][1,3]dioxol-4-yl}methanol (**16**, 0.016 g, 0.043 mmol) was dissolved in N,N-dimethylformamide (0.4 mL). Chlorosulfonamide (0.015 g, 0.129 mmol) was added and the mixture was stirred for 5 h at room temperature. The mixture was poured into saturated aqueous sodium bicarbonate solution and extracted with ethyl acetate twice. The extracts were combined, washed with water then brine, dried over sodium sulfate, filtered and concentrated under reduced pressure.

The above crude product was dissolved in a mixture of formic acid (0.4 mL) and water (0.4 mL) and stirred overnight at room temperature. The mixture was twice diluted with toluene and concentrated to ¼ volume. Subsequently, the moist mixture was coevaporated from acetonitrile until dry. The crude product was purified by C18 chromatography (95/5 to 30/70 water/acetonitrile gradient, with 10 mM ammonium acetate). Product-containing fractions were concentrated under reduced pressure. Water was removed through acetonitrile then toluene azeotrope to afford **ML901** (0.008 g, 44% yield over 2 steps). ^{1}H NMR (400 MHz, MeOD): δ 8.20 (s, 1H), 7.41 (t, J = 73.3 Hz, 1H), 6.25 (d, J = 2.5 Hz, 1H), 4.62-4.56 (m, 2H), 4.34-4.28 (m, 1H), 4.24-4.18 (m, 2H). ^{13}C NMR (101 MHz, DMSO-d_6): δ 158.19, 157.53, 155.33, 149.15, 115.69 (t, J = 260.1 Hz, 1C), 89.29, 88.54, 81.41, 73.65, 71.14, 69.85. HRMS (ESI): *m/z* calculated for C11H14F2N6O7S $[M + H]^+$: 413.0686. Found: 413.0683.

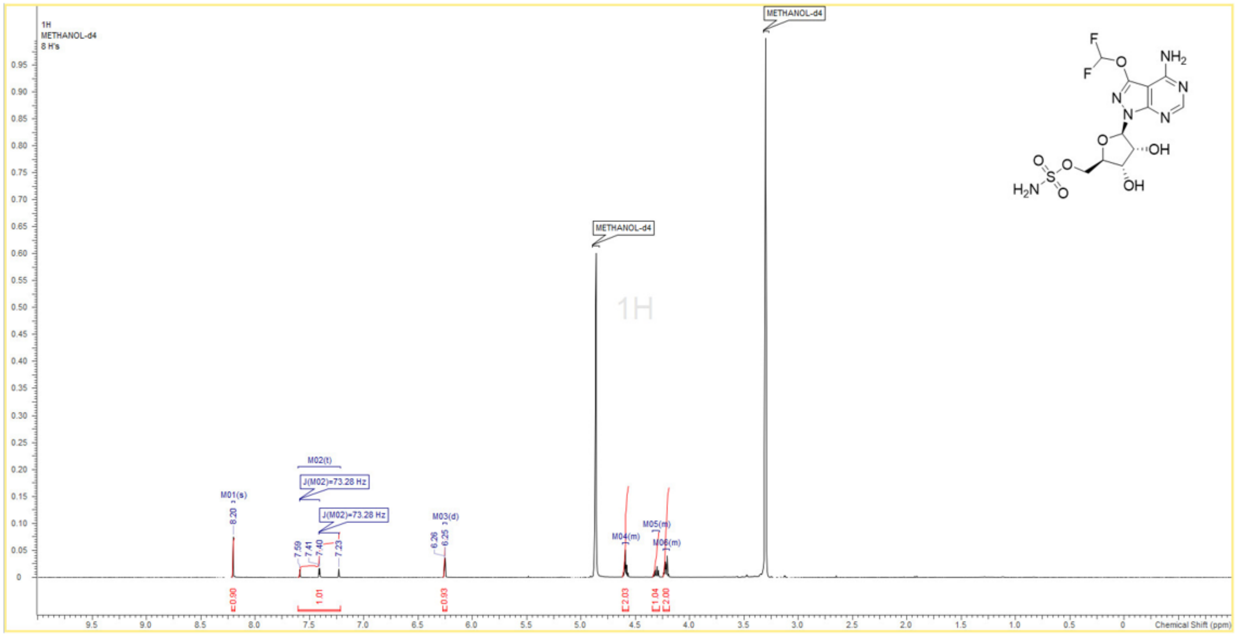

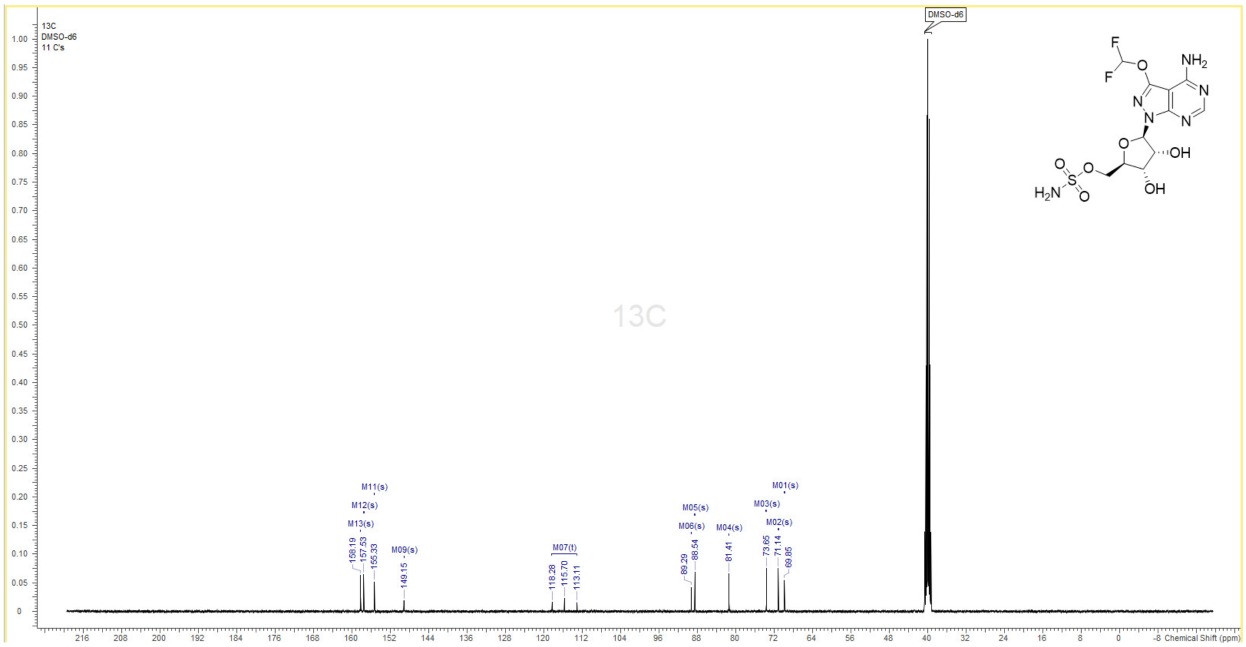

Synthesis of ML901-Tyr

Scheme 2. Synthesis of ML901-Tyr

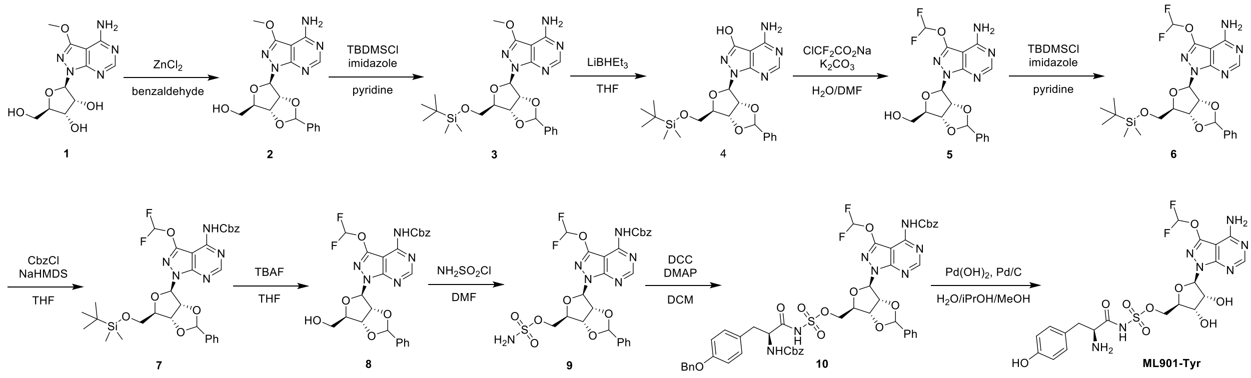

[(3aR,4R,6R,6aR)-4-(4-amino-3-methoxy-pyrazolo[3,4-d]pyrimidin-1-yl)-2-phenyl-3a,4,6,6a-tetrahydrofuro[3,4-d][1,3]dioxol-6-yl]methanol (2).

(2R,3R,4S,5R)-2-{4-amino-3-methoxy-1H-pyrazolo[3,4-d]pyrimidin-1-yl}-5-(hydroxymethyl)oxolane-3,4-diol (prepared as described in *(42)*, **1**, 7.3 g, 24.5 mmol) and zinc chloride (16.6 g, 121.8 mmol) was suspended in 70 mL freshly distilled benzaldehyde. The reaction mixture was stirred at room temperature for 72 h under N_2 atmosphere. The mixture was poured into saturated aqueous sodium bicarbonate solution and extracted twice with ethyl acetate. The extracts were combined, washed with brine and then dried over sodium sulfate, filtered and concentrated under reduced pressure. The crude product was purified by column chromatography (20/1 to 0/100 petroleum ether/ethyl acetate gradient) to afford **2** (6.6 g, 70 % yield) as a white solid. LC-MS (*m/z*): 386.2 $[M + H]^+$. ^1H NMR (400 MHz, $CDCl_3$): δ 8.25 (s, 1H), 7.60 (dd, J=6.5, 2.9 Hz, 2H), 7.43-7.48 (m, 3H), 6.62 (d, J=2.8 Hz, 1H), 5.99 (s, 1H), 5.78 (br s, 2H), 5.35 (dd, J=6.2, 2.9 Hz, 1H), 5.17 (d, J=6.0 Hz, 1H), 4.68 (s, 1H), 4.08 (s, 3H), 3.91-4.01 (m, 1H), 3.74-3.88 (m, 1H).

1-[(3aR,4R,6R,6aR)-6-[[tert-butyl(dimethyl)silyl]oxymethyl]-2-phenyl-3a,4,6,6a-tetrahydrofuro[3,4-d][1,3]dioxol-4-yl]-3-methoxy-pyrazolo[3,4-d]pyrimidin-4-amine (3).

[(3aR,4R,6R,6aR)-4-(4-amino-3-methoxy-pyrazolo[3,4-d]pyrimidin-1-yl)-2-phenyl-3a,4,6,6a-tetrahydrofuro[3,4-d][1,3]dioxol-6-yl]methanol (**2**, 7.46 g, 19.3 mmol), imidazole (11.7 g, 171.8 mmol) and *tert*-butyldimethylsilyl chloride (12.4 g, 82.9 mmol) were stirred in dry pyridine (70 mL) for 3 h at room temperature under N_2 atmosphere. The reaction was quenched by the addition of ice-water and the aqueous phase was extracted with dichloromethane three times. The organic phases were combined, washed with water, brine and then dried over sodium sulfate, filtered and concentrated under reduced pressure. The crude product was purified by column chromatography (20/1 to 0/100 petroleum ether/ethyl acetate gradient) to afford **3** (8.5 g, 84 % yield) as a white solid. LC-MS (*m/z*): 500.2 $[M + H]^+$. ^1H NMR (400 MHz, $CDCl_3$): δ 8.32 (s, 1H), 7.56-7.62 (m, 2H), 7.40-7.45 (m, 3H), 6.62 (d, J=2.0 Hz, 1H), 6.00 (s, 1H), 5.68 (br s, 2H), 5.54 (dd, J=6.4, 1.8 Hz, 1H), 5.09 (dd, J=6.5, 2.0 Hz, 1H), 4.43 (dd, J=5.3, 2.9 Hz, 1H), 4.07 (s, 3H), 3.81 (dd, J=10.3, 8.3 Hz, 1H), 3.67 (dd, J=10.3, 5.4 Hz, 1H), 0.83-0.91 (m, 9H), 0.02 (s, 6H).

1-[(3aR,4R,6R,6aR)-6-[[tert-butyl(dimethyl)silyl]oxymethyl]-2-phenyl-3a,4,6,6a-tetrahydrofuro[3,4-d][1,3]dioxol-4-yl]-4-amino-pyrazolo[3,4-d]pyrimidin-3-ol (4).

To a solution of 1-[(3aR,4R,6R,6aR)-6-[[tert-butyl(dimethyl)silyl]oxymethyl]-2-phenyl-3a,4,6,6a-tetrahydrofuro[3,4-d][1,3]dioxol-4-yl]-3-methoxy-pyrazolo[3,4-d]pyrimidin-4-amine (**3**, 8.5 g, 17.0 mmol) in THF (400 mL) at rt was added a solution of lithium triethylborohydride in THF (1.0 M, 45.0 mL, 45.0 mmol) dropwise. Upon completion of addition, the reaction mixture was allowed to stir at 80°C for 2 h in a heated oil bath. Upon completion of reaction, the flask was allowed to cool in an ice-water bath. Excess hydride reagent was carefully quenched by the slow addition of water (50 mL) over the period of 1 h. The reaction mixture was made neutral by the addition of a saturated aqueous ammonium chloride solution (200

mL). The mixture was transferred to a separatory funnel and extracted with ethyl acetate three times. The combined extracts were washed with saturated sodium bicarbonate solution and brine, dried over sodium sulfate, filtered and concentrated under reduced pressure. The crude product was purified by silica gel chromatography (100/1 to 5/1 dichloromethane/methanol gradient) to afford **4** (7.6 g, 83 % yield) as a white solid. LC-MS (*m/z*): 486.2 $[M + H]^+$. ^{1}H NMR (400 MHz, $CDCl_3$): δ 8.32 (s, 1H), 7.49-7.57 (m, 2H), 7.40-7.46 (m, 3H), 6.40 (d, J=1.3 Hz, 1H), 6.31 (br s, 2H), 5.97 (s, 1H), 5.29 (s, 1H), 5.03 (dd, J=6.4, 2.4 Hz, 1H), 4.42-4.52 (m, 1H), 3.75-3.93 (m, 2H), 0.90-0.95 (m, 9H), 0.09-0.18 (m, 6H).

[(3aR,4R,6R,6aR)-4-[4-amino-3-(difluoromethoxy)pyrazolo[3,4-d]pyrimidin-1-yl]-2-phenyl-3a,4,6,6a-tetrahydrofuro[3,4-d][1,3]dioxol-6-yl]methanol (5).

To a solution of 1-[(3aR,4R,6R,6aR)-6-[[tert-butyl(dimethyl)silyl]oxymethyl]-2-phenyl-3a,4,6,6a-tetrahydrofuro[3,4-d][1,3]dioxol-4-yl]-4-amino-pyrazolo[3,4-d]pyrimidin-3-ol (**4**, 7.1 g, 14.6 mmol) in DMF (56 mL) was added potassium carbonate (6.6 g, 48.1 mmol), sodium chlorodifluoroacetate (7.3 g, 48.1 mmol) and water (14.1 mL). The resulting mixture was degassed by active argon bubbling through the stirred reaction mixture. The degassed reaction mixture was stirred vigorously and placed in a pre-heated (80°C) oil bath and stirred overnight. Upon completion, the reaction mixture was allowed to cool to rt. The heterogeneous mixture was partitioned between ethyl acetate (300 mL) and water (300 mL) to which was added 200 mL saturated aqueous ammonium chloride solution. The phases were separated and the washings were extracted with additional ethyl acetate (300 mL). The extracts were combined, washed with water and brine, then dried over sodium sulfate, filtered and concentrated under reduced pressure. The crude product was purified by silica gel chromatography (100/1 to 5/1 dichloromethane/methanol gradient) to afford **5** (4.0 g, 57 % yield) as a yellow solid. LC-MS (*m/z*): 422.2 $[M + H]^+$. ^{1}H NMR (400 MHz, $CDCl_3$): δ 8.35 (s, 1H), 7.57-7.62 (m, 2H), 7.45-7.48 (m, 3H), 6.99-7.17 (m, 1H), 6.49 (d, J=3.5 Hz, 1H), 6.02 (s, 1H), 5.35 (dd, J=6.3, 3.5 Hz, 1H), 5.15 (dd, J=6.2, 1.3 Hz, 1H), 4.66 (s, 1H) 3.88-3.97 (m, 1H) 3.77-3.83 (m, 1H).

1-[(3aR,4R,6R,6aR)-6-[[tert-butyl(dimethyl)silyl]oxymethyl]-2-phenyl-3a,4,6,6a-tetrahydrofuro[3,4-d][1,3]dioxol-4-yl]-3-(difluoromethoxy)pyrazolo[3,4-d]pyrimidin-4-amine (6).

[(3aR,4R,6R,6aR)-4-[4-amino-3-(difluoromethoxy)pyrazolo[3,4-d]pyrimidin-1-yl]-2-phenyl-3a,4,6,6a-tetrahydrofuro[3,4-d][1,3]dioxol-6-yl]methanol (**5**, 4.00 g, 8.35 mmol), 1H-imidazole (5.11 g, 75.1 mmol) and *tert*-butyldimethylsilyl chloride (5.41 g, 35.9 mmol) were stirred in dry pyridine (50 mL) for 3 h at room temperature under N_2 atmosphere. The reaction was quenched by the addition of ice-water and the aqueous phase was extracted with dichloromethane three times. The organic phases were combined, washed with water, brine and then dried over sodium sulfate, filtered and concentrated under reduced pressure. The crude product was purified by column chromatography (100/1 to 1/1 petroleum ether/ethyl acetate gradient) to afford **6** (3.5 g, 76 % yield) as a white solid. LC-MS (*m/z*): 536.1 $[M + H]^+$. ^{1}H NMR (400 MHz, $CDCl_3$): δ 8.38 (s, 1H), 7.55-7.62 (m, 2H), 7.41-7.46 (m, 3H), 7.03-7.21 (m, 1H), 6.63 (d, J=2.0 Hz, 1H), 6.01 (s,

1H), 5.83 (br s, 2H), 5.51 (dd, J=6.5, 2.0 Hz, 1H), 5.08 (dd, J=6.5, 2.0 Hz, 1H), 4.40-4.49 (m, 1H), 3.70-3.75 (m, 1H), 3.59-3.69 (m, 1H), 0.88 (s, 9H), 0.02 (s, 6H).

Benzyl N-[1-[(3aR,4R,6R,6aR)-6-[[tert-butyl(dimethyl)silyl]oxymethyl]-2-phenyl-3a,4,6,6a-tetrahydrofuro[3,4-d][1,3]dioxol-4-yl]-3-(difluoromethoxy)pyrazolo[3,4-d]pyrimidin-4-yl]carbamate (7).

To a solution of 1-[(3aR,4R,6R,6aR)-6-[[tert-butyl(dimethyl)silyl]oxymethyl]-2-phenyl-3a,4,6,6a-tetrahydrofuro[3,4-d][1,3]dioxol-4-yl]-3-(difluoromethoxy)pyrazolo[3,4-d]pyrimidin-4-amine (**6**, 1.33 g, 2.48 mmol) in THF (100 mL) at -60°C was added a solution of sodium bis(trimethylsilyl)amide in THF (1.0 M, 6.19 mL, 6.19 mmol). After stirred at -60°C for 30 min, a solution of benzyl chloroformate (0.71 mL, 4.96 mmol) in THF (3 mL) was added and stirring was continued for 2 h at the same temperature. Upon completion of reaction, the reaction mixture was added a saturated aqueous ammonium chloride solution (200 mL). The mixture was transferred to a separatory funnel and extracted with ethyl acetate three times. The combined extracts were washed with brine, dried over sodium sulfate, filtered and concentrated under reduced pressure. The crude product was purified by silica gel chromatography (10/1 to 1/1 petroleum ether/ethyl acetate gradient) to afford **7** (2.5 g, 75 % yield) as a white solid. LC-MS (*m/z*): 670.1 $[M + H]^+$. ^1H NMR (400 MHz, $CDCl_3$): δ 8.80 (br s, 1H), 7.53-7.61 (m, 2H), 7.38-7.50 (m, 9H), 7.02-7.20 (m, 1H), 6.68 (d, J=2.0 Hz, 1H), 6.02 (s, 1H), 5.51 (dd, J=6.5, 2.1 Hz, 1H), 5.32 (s, 2H), 5.08 (dd, J=6.4, 1.9 Hz, 1H), 4.43-4.52 (m, 1H), 3.57-3.78 (m, 2H), 0.88 (s, 9H), 0.01 (s, 6H).

Benzyl N-[1-[(3aR,4R,6S,6aR)-6-(hydroxymethyl)-2-phenyl-3a,4,6,6a-tetrahydrofuro[3,4-d][1,3]dioxol-4-yl]-3-(difluoromethoxy)pyrazolo[3,4-d]pyrimidin-4-yl]carbamate (8).

To a solution of benzyl N-[1-[(3aR,4R,6R,6aR)-6-[[tert-butyl(dimethyl)silyl]oxymethyl]-2-phenyl-3a,4,6,6a-tetrahydrofuro[3,4-d][1,3]dioxol-4-yl]-3-(difluoromethoxy)pyrazolo[3,4-d]pyrimidin-4-yl]carbamate (**7**, 2.80 g, 4.18 mmol) in THF (30 mL) at rt was added a solution of tetrabutylammonium fluoride in THF (1.0 M, 12.5 mL, 12.5 mmol). The resulting mixture was stirred at rt for 3 h. The reaction mixture was concentrated under reduced pressure and the crude residue was purified by silica gel chromatography (100/1 to 10/1 ethyl acetate/methanol gradient) to afford **8** (2.4 g, 93 % yield) as a white solid. LC-MS (*m/z*): 556.1 $[M + H]^+$. ^1H NMR (400 MHz, $CDCl_3$): δ 8.79 (br s, 1H), 7.55-7.63 (m, 2H), 7.45-7.51 (m, 5H), 7.38-7.44 (m, 4H), 6.97-7.16 (m, 1H), 6.59 (d, J=3.5 Hz, 1H), 6.03 (s, 1H), 5.35 (dd, J=6.4, 3.4 Hz, 1H), 5.32 (s, 2H), 5.15 (dd, J=6.3, 1.5 Hz, 1H), 4.66 (br s, 1H) 4.05 (dd, J=6.0, 2.5 Hz, 1H), 3.89-3.97 (m, 1H) 3.74-3.86 (m, 1H).

Benzyl N-[1-[(3aR,4R,6S,6aR)-2-phenyl-6-(sulfamoyloxymethyl)-3a,4,6,6a-tetrahydrofuro[3,4-d][1,3]dioxol-4-yl]-3-(difluoromethoxy)pyrazolo[3,4-d]pyrimidin-4-yl]carbamate (9).

To a solution of benzyl N-[1-[(3aR,4R,6S,6aR)-6-(hydroxymethyl)-2-phenyl-3a,4,6,6a-tetrahydrofuro[3,4-d][1,3]dioxol-4-yl]-3-(difluoromethoxy)pyrazolo[3,4-d]pyrimidin-4-yl]carbamate (**8**, 2.1 g, 3.4 mmol) in

DMF (50 mL) at rt was added sulfamoyl chloride (2.35 g, 20.4 mmol). The resulting mixture was stirred at rt for 3 h. The reaction was quenched by the addition of ice-water and the aqueous phase was extracted with ethyl acetate three times. The organic phases were combined, washed with brine and then dried over sodium sulfate, filtered and concentrated under reduced pressure. The crude product was purified by column chromatography (20/1 to 1/100 petroleum ether/ethyl acetate gradient) to afford **9** (1.7 g, 76 % yield) as a white solid. LC-MS (*m/z*): 635.1 $[M + H]^+$. ^{1}H NMR (400 MHz, DMSO-d_6): δ 11.06 (br s, 1H), 8.78 (br s, 1H), 7.51-7.58 (m, 5H), 7.46-7.49 (m, 3H), 7.41-7.45 (m, 4H), 6.61 (s, 1H), 6.03 (s, 1H), 5.41-5.48 (m, 1H), 5.19-5.30 (m, 3H), 5.15 (dd, J=6.3, 2.3 Hz, 1H), 4.59 (t, J=5.6 Hz, 1H), 4.23-4.31 (m, 1H), 4.12 (dd, J=10.8, 7.3 Hz, 1H).

((3aR,4R,6R,6aR)-6-(4-(((benzyloxy)carbonyl)amino)-3-(difluoromethoxy)-1H-pyrazolo[3,4-d]pyrimidin-1-yl)-2-phenyltetrahydrofuro[3,4-d][1,3]dioxol-4-yl)methyl ((S)-2-(((benzyloxy)carbonyl)amino)-3-(4-(benzyloxy)phenyl)propanoyl)sulfamate (10).

To a solution of (2S)-2-{[(benzyloxy)carbonyl]amino}-3-[4-(benzyloxy)phenyl]propanoic acid (0.859 g, 2.118 mmol) in dichloromethane (30 mL) at rt was added N,N′-dicyclohexylcarbodiimide (0.583 g, 2.827 mmol). The reaction mixture was stirred at rt for 1 h and then cooled to -40 °C. To the reaction mixture was added a solution of benzyl N-[1-[(3aR,4R,6S,6aR)-2-phenyl-6-(sulfamoyloxymethyl)-3a,4,6,6a-tetrahydrofuro[3,4-d][1,3]dioxol-4-yl]-3-(difluoromethoxy)pyrazolo[3,4-d]pyrimidin-4-yl]carbamate (**9**, 0.450 g, 0.709 mmol) and DMAP (0.259 g, 2.118 mmol) in dichloromethane (5 mL). The reaction mixture was stirred at -40 °C for 6 h. The reaction was quenched by the addition of ice-water (10 mL) and saturated aqueous sodium bicarbonate solution (10 mL). The aqueous phase was extracted with dichloromethane three times. The extracts were combined, washed with brine and then dried over sodium sulfate, filtered and concentrated under reduced pressure. The crude product was purified by preparative reverse phase HPLC to afford **10** (0.200 g, 26% yield) as a white solid. LC-MS (*m/z*): 1022.6 $[M + H]^+$.

[(2R,3S,4R,5R)-5-[4-amino-3-(difluoromethoxy)pyrazolo[3,4-d]pyrimidin-1-yl]-3,4-dihydroxy-tetrahydrofuran-2-yl]methyl N-[(2S)-2-amino-3-(4-hydroxyphenyl)propanoyl]sulfamate (ML901-Tyr).

To a solution of ((3aR,4R,6R,6aR)-6-(4-(((benzyloxy)carbonyl)amino)-3-(difluoromethoxy)-1H-pyrazolo[3,4-d]pyrimidin-1-yl)-2-phenyltetrahydrofuro[3,4-d][1,3]dioxol-4-yl)methyl ((S)-2-(((benzyloxy)carbonyl)amino)-3-(4-(benzyloxy)phenyl)propanoyl)sulfamate (**10**, 0.200 g, 0.195 mmol) in water/isopropanol/methanol (5 mL, 1/19/10) was added palladium on carbon (0.102 g, 10 wt. %) and palladium hydroxide on carbon (0.068 g, 20 wt. %). The mixture was allowed to stir under an atmosphere of hydrogen at 70°C for 16 h. The mixture was filtered through a pad of Celite and the filtrate was concentrated under reduced pressure. The crude product was purified by preparative reverse phase HPLC to afford **ML901-Tyr** (0.038 g, 30 % yield) as a white solid. LC-MS (*m/z*): 576.1 $[M + H]^+$. ^{1}H NMR (400 MHz, MeOD): δ 8.19 (s, 1H), 7.32-7.76 (m, 1H), 7.10 (d, J=8.4 Hz, 2H), 6.76 (d, J=8.4 Hz, 2H), 6.25 (d,

J=2.3 Hz, 1H), 4.63-4.67 (m, 2H), 4.60 (s, 1H), 4.14-4.28 (m, 3H), 3.72 (dd, J=8.6, 4.6 Hz, 1H), 3.19 (dd, J=14.6, 4.6 Hz, 1H), 2.89 (dd, J=14.5, 8.8 Hz, 1H). HRMS (ESI): *m/z* calculated for ML901-Tyr $C_{20}H_{23}F_2N_7O_9S + H^+$ $[M + H^+]$: 576.131879. Found: 576.1323.

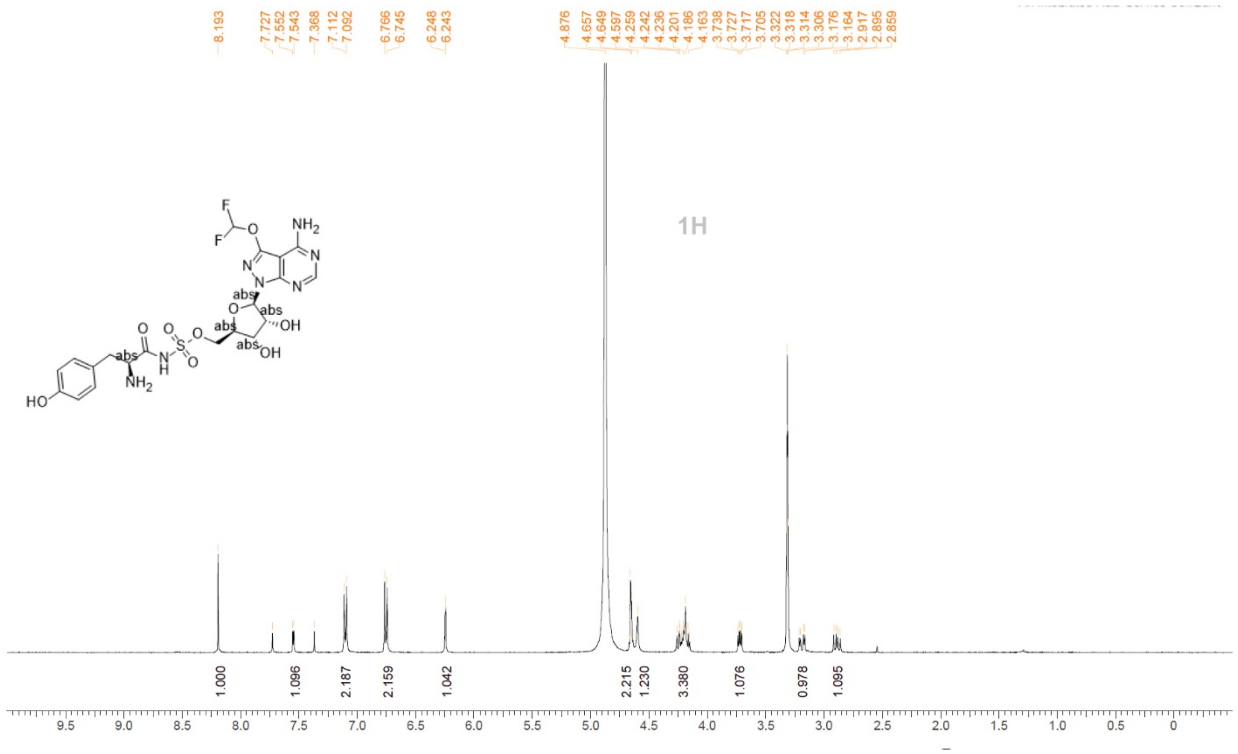

Biological methods

Screening of nucleoside sulfamates for inhibition of the growth of cultures of P. falciparum (3D7).

High throughput screening of compounds from the Takeda library was performed by SCYNEXIS, Inc. Jersey City, NJ, USA. Synchronized (5% sorbitol) ring stage parasites (3D7 strain; 1.5% haematocrit; 0.1 – 0.5% starting parasitemia) were incubated in 96-well plates, in triplicate, diluted in DMSO, for 72 h in RPMI 1640 supplemented with 10% human serum, 5% CO_2, 5% O_2, 90% N_2. Infected RBCs were lysed with 10 mM Tris HCl, 5 mM EDTA, 0.008% saponin, 0.08% Triton X-100, pH 7.5, containing SYBR Green. Following incubation for 15-60 min, fluorescence signal was assessed. Sigmoidal dose response curves were generated and the IC_{50} values determined using CLfit curve fitting software from IDBS (Guilford, UK).

Activity against laboratory strains and panels of drug resistant P. falciparum.

For repeat measurements of the activity of ML901 and assays of AMS activity, sorbitol-synchronsied ring stage parasites (3D7 strain (*43*)) were incubated with the inhibitors for 72 h and viability was assessed in the second cycle by flow cytometry, following labelling with 2 μM Syto-61 (Thermo Fisher Scientific), as described previously (*44, 45*). Viability represents the parasitemia normalized to untreated and "kill-treated" controls, where "kill-treated" refers to samples treated with 2 μM dihydroartemisinin (DHA;

Sigma-Aldrich) for 48-72 h. For drug pulse assays, tightly synchronized parasites (1-1.5% parasitemia, 0.2% final hematocrit) were added to the plates and incubated for 3 h. Drugs were removed and the parasitemia assessed in the trophozoite stage of the next cycle.

Compounds were tested against a range of drug resistant field isolates and laboratory strains using a modified [^{3}H]-hypoxanthine incorporation assay, as previously reported (*46*). Field isolates 7G8, Cam3.1, Dd2, K1, TM90C2B and NF54 were obtained from MR4 (www.beiresources.org).

Mammalian cell culture experiments

The HepG2 (Human Caucasian hepatocyte carcinoma) cell line was procured from ATCC (American Type Culture Collection, Manassas, USA; HB-8065) and viability assessed using Cell Titer-GLO luminescent cell viability assay reagents (Promega, catalogue number: G7570) (*47, 48*). For the assay, 2000 cells/well were plated in 384-well plates 24 h prior to the experiment and incubated at 37°C in a CO_2 incubator. The medium was removed and cells were treated with fresh medium containing either vehicle (0.5% DMSO) or serially diluted test compounds or doxorubicin (1.3 nM to 25 μM) in a final volume of 50 μl/well and further incubated for 72 h at 37°C in a CO_2 incubator. In the positive control wells (100% inhibition) cells were treated with 5 μl of 1% Triton X-100 (final assay conc. 0.1%). Following incubation, 25 μl of medium was discarded and 25 μl of Cell Titer-GLO reagent was added to each well and the plate was kept on a plate shaker for 15 min at 25°C with shaking at 300 rpm. Luminescence signals were measured in a Spectramax M5 reader (Molecular Devices).

H1650 is a human lung cancer derived ATG7-null cell line (*49*) that can be used to assess effects on targets other than ATG7. (ATCC: CRL-5883 NCI-165, Cat# CRL-5883 Lot : 3881497 (human lung adenocarcinoma; bronchoalveolar carcinoma). Other cells lines used are: H460 (NCI-H460; human lung; carcinoma; large cell lung cancer; ATCC: HTB-177); and HCT116 (human colon; colorectal carcinoma; ATCC: CCL-247; Lot : 5056139). Each of the cell lines was received from the American Type Culture Collection (ATCC) and passaged twice before freezing. Cells were cultured in the presence or absence of inhibitors and cell viability was assessed by comparing the ATP content in compound-treated wells with that in DMSO-treated wells as previously described (*11*).

HeLa cells (human cervical adenocarcinoma; genome sequenced (Garvin Institute, Australia) and verified as 100% match with ATCC HELA CCL) for cellular target ID studies, were maintained in a 10% CO_2, 37°C humidified incubator as adherent semi-confluent monolayers in complete Dulbecco's Modified Eagle Media (c-DMEM). DMEM (Gibco™, Thermo Fisher Scientific, Australia) was supplemented with 10% (v/v) fetal bovine serum (FBS), 100 units/ μL of penicillin and 100 μg/ml of streptomycin (Sigma-Aldrich, Australia); and 2 mM L-glutamine (Gibco™, Thermo Fisher Scientific, Australia). HeLa cells were verified

by genomic sequencing and were routinely tested for mycoplasma contamination using MycoAlert™ Mycoplasma Detection Kit (Lonza, Capsugel Australia).

E1-E2 Transthiolation Assays

An Homogeneous Time-Resolved Fluorescence (HTRF) enzyme assay was employed to evaluate compound activity against ATG7 as previously described (*11*). In this assay, a Flag-tagged ATG8 homolog (GABA type A receptor-associated protein; GABARAP) is activated by ATG7 and then transthiolated to a GST-tagged E2 (ATG3). The product of the enzyme reaction, *Flag*-GABARAP-ATG3-*GST*, is quantified by measuring FRET between Europium-Cryptate labelled monoclonal anti-Flag M2 (CisBio International, Cat# 61FG2KLB) and PHYCOLINK goat anti-GST allophycocyanin (Prozyme, PJ252p1). The activation and transthiolation of ubiquitin by Ubiquitin Activating Enzyme, activation and transthiolation of NEDD8 by NEDD8 Activating Enzyme. Activation and transthiolation of SUMO by SUMO Activating Enzyme were all assayed in a similar fashion with appropriately tagged ubiquitin-like proteins and E2 conjugating enzymes as described (*23, 50*).

Activity against transmissible stages of P. falciparum

Activity against male gametes was assessed using the dual gamete formation assay (*Pf*DGFA)(*32*). Compounds were incubated with mature stage V gametocytes (NF57, kind gift from Professor Chris Janse at Leiden University Medical Center (LUMC), the Netherlands) for 48 hours in 384 well plates before gamete formation was triggered by a drop in temperature and addition of xanthurenic acid. Male gamete exflagellation was recorded and quantified by automated microscopy. The ability of female gametes to express *Pf*s25 on their surface after 24 h at 28°C was assessed by single-image fluorescence microscopy. Multiple data sets using different gametocyte cultures were collected on each of two separate days.

Activity against exoerythrocytic stages

Primary human hepatocytes (H1500.H15B+ Lot No. HC10-10 Cryopreserved human hepatocytes, female) were cultured for 2 days and then overlaid with *P. falciparum* NF54 sporozoites and compounds. Supernatant was refreshed daily with fresh compounds. 4 days post-infection, hepatocytes were stained for the presence of liver stage parasites (*33*).

P. falciparum humanized NOD-scid IL2Rnull mouse model

The model using *P. falciparum Pf3D7*$^{0087/N9}$ in NODscidIL2Rγ^{null} mice engrafted with human RBCs was adapted from a previously described procedure (*51*). Female NODscidIL2Rγ^{null} mice were purchased from Charles River (Germany). *3D7*$^{0087/N9}$ was kindly provided by Javier Gamo (GSK, Tres Cantos, Spain). Briefly, two engrafted mice/dosing group (females, 20 - 22 g) were infected intravenously with 2 x 10^7 *P. falciparum* (*Pf3D7*$^{0087/N9}$) on day 0. The antimalarial efficacy was assessed following administration (i.p.)

of 50 mg/kg of compound on day 3 post-infection and measuring the effect on blood parasitemia by microscopic analysis of Giemsa-stained blood smears (on days 3, 4, 5, 6 and 7 post-infection). Mice were euthanized on day 7.

Ethics approvals

Human biological samples were sourced ethically; and their research use was in accord with the terms of the informed consent. All animal studies were ethically reviewed and carried out in accredited facilities in accordance with the relevant country's directives and the institution's Policy on the Care, Welfare and Treatment of Animals.

Plasma exposure in infected mouse model

Compound was administered orally to two mice at 25 mg/kg on days 3, 4, 5, 6 after infection. On day 3, blood samples (20 μL) were obtained at time points up to 24 hours after the first administration. Protein was precipitated with acetonitrile and the remaining compound was assessed by LC-MS/MS in the selected reaction monitoring mode using HESI ionization in positive ion mode.

Rat pharmacokinetics (PK) analyses

Sprague-Dawley rats (11 weeks old) were sourced from Hilltop Lab Animals, Inc (Scottdale, Pennsylvania, USA). ML901 was formulated in EtOH: DMAC: PEG400: H_2O at 1:1:4:4 (v/v) and 10% captisol in 50 mM citrate (pH 3.3) for i.v. (1 mg/kg) and p.o. (10 mg/kg) administration to male Sprague-Dawley rats (n = 3 per route of administration). Blood was collected from a jugular cannula at 0.083, 0.25, 0.5, 1, 2, 4, 8 and 24 hours post i.v. dosing and at the same times (except the 0.083-hour sample) following oral administration. A portion of the blood samples were processed into plasma. Samples were precipitated with 0.5% formic acid in methanol and the supernatants were analysed by positive ion electrospray LC-MS for the administered compound. Non-compartmental pharmacokinetic parameters were calculated from individual concentration *vs* time profiles using Phoenix 64 (WinNonlin) Version 8.1 Certara, Princeton NJ.

In vitro evolution of P. falciparum with reduced sensitivity to ML901

Multiple cloned *P. falciparum* 3D7 lines (*43*) were subjected to gradually increasing concentrations (up to 16 nM) of ML901 in culture over 4 months. One culture exhibited an ~10-fold increase in the 50% inhibitory concentration, indicating that resistance-conferring mutations had occurred.

Whole genome sequencing and analysis of ML901-resistant parasites

The sequencing library for parasite genomic DNA was prepared with the Nextera XT kit (Cat. No. FC-131-1024, Illumina) following the standard dual index protocol. The library was sequenced at the UC San Diego IGM Genomics Center on the Illumina HiSeq 2500 in RapidRun mode to generate 100bp paired-end reads.

Fastq files were aligned to the *P. falciparum* 3D7 reference genome (PlasmoDB v13.0) using the previously described Platypus pipeline (*52*). The four clones generated in the study (one parent clone and three ML901-resistant clones) were sequenced to an average depth of 122.3x.

SNVs and INDELs were called against the 3D7 reference genome using GATK HaplotypeCaller and filtered according to GATK recommendations (*53*). Briefly, SNVs were retained if they met the following filter criteria: ReadPosRankSum >8.0 or <-8.0, QUAL<500, Quality by Depth (QD) <2.0, Mapping Quality Rank Sum <-12.5, and filtered depth (DP) <7. INDELs were retained if they passed ReadPosRankSum <-20, QUAL<500, QD<2, and DP<7. SnpEff was used to annotate variants in the resulting VCF file (*54*). Variants with passing quality metrics and ≥90% allele frequency were further filtered to remove mutations that were also present in the 3D7 parent clone, as these would not have evolved over the course of ML901 selection. Each resistant clone contained 2-4 mutations that met all filtering criteria.

Generation and analysis of PfYRS knock-down transfectants

A conditional regulatable line for the cytosolic *Pf*YRS (PF3D7_0807900) was generated by integrating the gene regulatable system, TetR-DOZI-RNA aptamer module at the 3'-end of the target gene, in *P. falciparum* parasites using CRISPR-Cas9 and homology-driven repair (*55, 56*). A line harbourbing an aptamer-regulatable fluorescent protein (eYFP) integrated in the *cg6* locus is used as the control (*55*). Transfection into parasites was carried out by preloading RBCs with the donor vector as described previously (*57*). Briefly, 50 µg of purified plasmid DNA was mixed with human RBCs and subjected to 8 square wave electroporation pulses of 365 V for 1 ms each, separated by 0.1 s in a 0.2 cm cuvette. The plasmid DNA preloaded cells were inoculated with NF54 expressing Cas9 and T7 RNA polymerase. Cultures were maintained in 500 nM anhydrotetracycline (aTc; Sigma-Aldrich 37919) and the selectable drug 2.5 µg/mL of Blasticidin (RPI Corp B12150-0.1), and recovered parasites were monitored via Giemsa smears and RLuc measurements.

Assessment of parasite proliferation rate over two intraerythrocytic developmental cycles (IDC) by titrating the expression of YRS^{cyt} were carried out by maintaining the cultures in varying aTc concentrations and using luminescence as a readout of growth. In a 96-well U-bottom BD Falcon™ plate, synchronous ring-stage parasites were set up in triplicate and cultured in the presence (50 nM) and absence of aTc and expansion was measured at 0, 72, and 120 h by quantitating luminescence using the Renilla-Glo(R) Luciferase Assay System (ref no E2750, Promega) and the GloMax® Discover Multimode Microplate Reader (Promega). The luminescence values were normalized to chloroquine-treated (200 nM) samples, and results were visualized on a scatter plot using GraphPad Prism (version 8; GraphPad Software).

Stock solutions of ML901, DHA or borrelidin (Cayman Chemicals) were dispensed into 96-well U-bottom BD Falcon™ plates and serially diluted in complete medium to yield final concentrations ranging from 20-0.08 nM. Synchronous ring-stage YRS^{cyt} conditional knockdown line and the aptamer-regulatable control line in varying aTc concentrations (high = 50 or 500 nM, low = 1 nM, and no aTc) were distributed into the drug plate. Lowest drug treatment and a lethal dose of chloroquine (200 nM) served as reference controls. Growth inhibition was analysed after 72 h using the Renilla-Glo(R) Luciferase Assay System (ref no E2750 Promega) and the GloMax® Discover Multimode Microplate Reader (Promega), and IC_{50} values were obtained from corrected dose-response curves using GraphPad Prism (version 8; GraphPad Software).

Generation of PfYRS$_{S234C}$ transfectants

CRISPR/Cas9 was used to generate parasites encoding the S234C mutation in exon 2 of *Pf*YRS, as shown in Suppl Figure 6. Two guide RNAs (gRNA1: CTGGCAACATACCATGGGAA and gRNA2: TAAACCTGGCAACATACCAT) were selected using Benchling (benchling.com), and cloned into the pDC2-coCas9-gRNA plasmid essentially as described previously (*58*). A donor template of 645 bp, encompassing the majority of the 708 bp exon 2, was synthesized (ThermoFisher) and cloned into the *Aat*II/*Eco*RI sites of both the pDC2-coCas9-gRNA1 and pDC2-coCas9-gRNA2 plasmids. In addition to the TCC>TGC (S234C) mutation, additional silent shield mutations were introduced to prevent gRNA-Cas9 complex binding, as shown in Suppl Figure 6. Ring-stage *P. falciparum* parasites (Dd2 strain) at 10% parasitemia (150 µL of packed cells) were transfected with 50 µg of plasmid DNA using a BioRad Gene Pulser II (310 V, 950 µF), and selected with 5 nM WR99210 (Jacobus Pharmaceuticals) for 8 days, after which culture was continued without drug pressure until transfectants were obtained. Clones of the *Pf*YRS$_{S234C}$ mutant parasites were isolated by limiting dilution and verified by Sanger sequencing.

Protein translation assay

P. falciparum Cam 3.II_Rev (*25*) schizont- (43-48 h p.i.) infected RBCs were pre-incubated with inhibitor for 1 h at 0.2% hematocrit and 1% parasitemia. Using a modification of a published procedure (*14*), the cells were labelled with O-propargyl-puromycin (OPP; 4 µM) for 1 h, washed 3x (PBS +3% serum) and then fixed for 10 minutes (4% formaldehyde/0.02% glutaraldehyde in PBS). The click reaction was performed for 1 h in the presence of $CuSO_4$ (0.1 mM), THPTA (0.5 mM) and sodium ascorbate (5 mM) to bring about azide-alkyne cycloaddition to Alexa Fluor 488 azide (0.1 µM). Flow cytometry was performed using the FITC and Cy™5.5 channels to detect the Alexa Fluor 488 and PI, respectively.

Western blotting

Cam 3.II_Rev (*25*) trophozoite-infected RBCs (25-30 h p.i.) were incubated with inhibitor for 3 h at 10% hematocrit and 5% parasitemia. The RBC pellet was washed 3 x in PBS + cOmplete. Cells were centrifuged at 380 x g for 5 min and the pelleted cells lysed by resuspension in PBS + 0.05% saponin for 5 min on ice.

The samples were centrifuged at 380 x g for 10 min at 4°C. The wash with PBS + cOmplete and centrifugation (380 x g, 10 min, 4°C) was repeated 3 times. The pellet was resuspended in Bolt LDS sample buffer plus reducing agent. The samples were vortexed for 10 min at room temperature.

Membranes were blocked with 5% (w/v) skim milk for 1 h at room temperature and probed with primary antibody overnight at 4°C, followed by secondary antibody for 1 h at room temperature. Primary antibodies: rabbit anti-phospho-eIF2α (Cell Signaling Technology-119A11; 1:1,000); polyclonal mouse anti-*Pf*BiP, generated using recombinant *Pf*BiP at the WEHI Antibody Services (1:1,000). Secondary antibodies: goat anti-rabbit IgG-peroxidase (Sigma-Aldrich-A0545; 1:20 000), goat anti-mouse IgG-peroxidase (Chemicon-AP127P; 1:20,000). Chemiluminescence was detected using the Bio-Rad ChemiDoc^{TM} MP imaging system.

The *eIK1* knock-out line was kindly provided by Prof Christian Doerig (RMIT University) (*16*).

Preparation of P. falciparum YRS and human mature YRS

The *Pf*YRS construct comprised a 6x histidine tag at the N-terminus followed by a TEV protease cleavage sequence and the *Pf*YRS protein sequence (ID: PF3D7_0807900) from *P. falciparum*. The *Pf*YRS$_{S234C}$ construct was generated by site-directed mutagenesis (GenScript). For the human mature YRS construct, the same His-tag and Tev cleavage sequence were added to the N-terminus of the human mature YRS sequence (amino acids 1-364) (Uniprot ID: P54577). The YRS sequences were codon optimized for *E. coli*, synthesized and cloned into the pET-11a expression vector (GenScript). The vector was transformed into *E. coli* BL21(DE3) cells and expressed in 2 L of LB media. *Pf*YRS, *Pf*YRS$_{S234C}$ and *Hs*YRS were induced for 3 h at 37°C with 0.1 mM and 0.5 mM IPTG respectively. Cell pellets were resuspended in 40 mL lysis buffer containing 50 mM Tris-HCl, pH 8, 500 mM NaCl, 50 mM imidazole, 1 mM TCEP, 1 mg/mL lysozyme and 1x protease inhibitor cocktail (Roche). Cells were lysed by sonication (Microtip, QSonica) and the lysate was clarified by centrifugation at 30,000 *g* for 25 min. The supernatant was applied to a 5 mL HisTrap HP column (GE Healthcare) and washed with 50 mL binding buffer containing 50 mM Tris-HCl, pH 8, 500 mM NaCl, 50 mM imidazole, and 1 mM TCEP. His-YRS was eluted using a 0-500 mM imidazole gradient in the above buffer over 100 mL. The eluted His-YRS was dialyzed overnight at 4°C against 50 mM Tris-HCl, pH 8, 500 mM NaCl, 30 mM imidazole, 1 mM TCEP with the addition of His-tagged TEV protease (L56V/S135G/S219V triple-mutant (*59*)). Cleaved His tags from His-YRS and TEV protease were removed by running the dialyzed protein through a HisTrap HP column and collecting the flow-through. YRS was further purified by gel filtration using a HiLoad 16/600 Superdex 200 column (GE Healthcare). The molecular mass of purified YRS was verified using an Agilent 6220 Accurate-Mass ESI-TOF mass spectrometer after online desalting.

Preparation of ML901-Tyr-bound P. falciparum YRS

Recombinant *Pf*YRS was used without removal of the His-tag. *Pf*YRS (0.25 mg/ml was incubated in the presence of ATP (25 μM), tyrosine (50 μM), ML901 (150 μM) and *E. coli* tRNA (7 mg/ml), in 25 mM Tris, pH 8, 150 mM NaCl, 5 mM $MgCl_2$, 1 mM TCEP at 37°C for 3.5 h. The complex was bound to a HisTrap HP column in the presence of 50 mM Tris, pH 8, 150 mM NaCl, 30 mM imidazole, 1 mM TCEP and eluted using a 0-500 mM imidazole gradient in 50 mM Tris, pH 8, 150 mM NaCl, 1 mM TCEP. The eluted complex was concentrated and further purified by gel filtration using a HiLoad 16/600 Superdex 200 column (GE Healthcare). Formation of a complex with the ML901-Tyr conjugate was verified using DSF.

Sedimentation velocity analysis

*Pf*YRS, *Pf*YRS_{S234C} or *Hs*YRS samples were diluted to 0.1, 0.25 or 0.5 mg/mL in 25 mM Tris-HCl, pH 8, 150 mM NaCl and 1 mM TCEP. 380 μL aliquots at each concentration were loaded into double-channel quartz window cells (Beckman Coulter), with the above buffer in the reference compartment. Cells were centrifuged at 50,000 rpm (201,600 *g*) at 20°C using an XL-I analytical ultracentrifuge (Beckman Coulter) or an Optima analytical ultracentrifuge (Beckman Coulter). Radial absorbance data were acquired at a wavelength of 235 or 280 nm (as indicated), with radial increments of 0.003 cm, in continuous scanning mode. The sedimenting boundaries were fitted to a model that describes the sedimentation of a distribution of sedimentation coefficients with no assumption of heterogeneity (c(s)) using the program SEDFIT (*60*). Data were fitted using a regularization parameter of $p = 0.95$, floating frictional ratios, and 250 sedimentation coefficient increments.

Small Angle X-Ray Scattering (SAXS) analysis

Small angle X-ray scattering (SAXS) analyses were conducted at the Australian Synchrotron SAXS/WAXS beamline, using co-flow to reduce radiation damage and allow higher X-ray flux onto the sample, and an optimized chromatography system to limit sample dilution (*35-37*). Data were collected from purified *Pf*YRS, *Pf*YRS_{S234C}, and *Hs*YRS without addition of exogenous ligand. The sample-to-detector distance was 2210 mm, and the X-ray beam energy was 11,500 eV (λ = 1.078 Å), providing a total q range of 0.005 – 0.54 $Å^{-1}$, $q = (4\pi\sin\theta)/\lambda$. Data were collected following fractionation using an in-line size-exclusion chromatography column (Superdex 200 5/150 Increase; GE Healthcare) pre-equilibrated in buffer containing 20 mM Tris-HCl, pH 7.4, 150 mM NaCl and 5 mM TCEP. YRS samples were loaded at 5 mg/mL. Data were collected from a 1.5 mm capillary under continuous flow, with frames collected every second. Data reduction and buffer subtraction were performed using the Scatterbrain software, and data was further analysed using the ATSAS suite (*61*). Theoretical scattering profiles from the YRS crystal structures were calculated and fitted to the experimental scattering data using *CRYSOL* (*62*). A summary of the SAXS data acquisition and processing parameters is provided in Suppl Table 6.

In vitro transcription/ translation of Pf$tRNA^{Tyr}$ *and Hs*$tRNA^{Tyr}$

An intron was detected in the *Pf*tRNA^{Tyr} sequence, that was not indicated in PlasmoDB. A T7 RNA polymerase promoter sequence was added to the 5' end of the DNA sequence of *P. falciparum* and *H. sapiens* tRNA^{Tyr}. This DNA template and its complementary strand were custom-synthesized by Sigma-Aldrich. Two oligonucleotides were annealed at 95°C for 5 min and the double-stranded DNA template was used for *in vitro* transcription. The transcription reaction was incubated at 37°C overnight. The reaction mixture consists of template DNA, T7 RNA polymerase and NTP mix as per manufacturer's instructions (HiScribe™ T7 Quick High Yield RNA Synthesis Kit, NEB). On the following day, the reaction mixture was treated with DNase I at 37°C for 15 min. tRNA was purified using RNA clean-up spin columns (Monarch® RNA Cleanup Kit, NEB) to remove contaminants from the transcription reaction.

Differential scanning fluorimetry (DSF)

*Pf*YRS and *Hs*YRS at 0.1 mg/ml were incubated with various substrates at 37°C for 1-3 h. *Pf*YRS was assayed in 25 mM Tris, pH 8, 150 mM NaCl, 5 mM $MgCl_2$ and 1 mM TCEP. *Hs*YRS was assayed in 50 mM Tris, pH 7.5, 150 mM NaCl, 50 mM KCl, 5 mM $MgCl_2$ and 1 mM TCEP. SYPRO Orange (Sigma-Aldrich; 5,000X concentrate in DMSO) was added to the reaction mixture at a final concentration of 2.5X. 25 µL of the sample was added into each well of a 96-well qPCR plate (Applied Biosystems). The plate was sealed and analysed using StepOnePlus Real-Time PCR system (Applied Biosystems). The samples were heated from 20°C to 90°C with a 1% continuous gradient. The thermal unfolding curve was plotted as the first derivative curve of the raw fluorescence values. The melting temperature (T_m), defined as the peak of the first derivative curve, was used to assess the thermal stability of protein-ligand complexes. K_D values were calculated from DT_m using the equation: $\ln(K_D/[L]) = -131.5 \text{ x } (\Delta T_m/T_{m,\text{apo}}) - 1.9$, where $[L]$ is the concentration of ligand and T is the temperature in Kelvin (*40*).

ATP consumption of PfYRS, PfYRS$_{S234C}$ and HsYRS

Consumption of ATP by YRS was measured using a luciferase-based assay as per the manufacturer's instructions (Kinase-Glo Luminescent Kinase Assay, Promega). Reactions were conducted in 50 mM Tris-HLC pH 7.6, 50 mM KCl, 25 mM $MgCl_2$, 0.1 mg/mL BSA, 1 mM DTT, with 100-200 µM L-tyrosine, 24 µM *Pf*tRNA^{Tyr} (*Pf*YRS and *Pf*YRS$_{S234C}$) or 33 µM human tRNA^{Tyr} (human YRS), 10 µM ATP (unless stated otherwise), 0.2 – 0.25 µM enzyme and 1 unit/mL inorganic pyrophosphatase. Reactions were incubated at 37°C for 1 h (unless stated otherwise).

Aminoacylation of PfYRS, PfYRS$_{S234C}$ and HsYRS

The aminoacylation activity of YRS was measured by the incorporation of tritiated L-tyrosine into tRNA^{Tyr} using an adaption from a published protocol (*19*). Reactions were conducted in 50 mM Tris-HCL, pH 7.6, 50 mM KCl, 25 mM $MgCl_2$, 0.1 mg/mL BSA, 1 mM DTT, with 97 µM L-tyrosine and 3 µM L-[3,5-^{3}H]

tyrosine, 24 μM *Pf*tRNA^{Tyr} (*Pf*YRS and *Pf*YRS$_{S234C}$) or 33 μM human tRNA^{Tyr} (human YRS), 10 μM ATP (unless stated otherwise), 0.2 – 0.25 μM enzyme, 1 unit/mL inorganic pyrophosphatase. Reactions were initiated with the addition of YRS and incubated for at 37°C for 1 h, followed by the collection of samples by spotting 15 μL onto Whatman GF/C glass microfiber filters. Reactions were quenched and product captured by TCA precipitation (10% TCA (10 minutes), 5% TCA (10 minutes), 5% TCA (20 minutes), 95% ethanol (10 minutes)), followed by measurement of incorporated L-[3,5-^{3}H]tyrosine (corresponding to amino acid ligated to tRNA^{Tyr} substrate) by liquid scintillation.

Mass spectrometry to identify and quantify the ML901-tyrosine conjugate

YRS reactions were set up with following components: 2.3 μM *Pf*YRS/*Hs*YRS, 20 μM tyrosine, 10 μM ATP, 50 μM ML901 and 8 μM *Pf*tRNA^{Tyr} or 32 μM *Hs*tRNA^{Tyr}. The *Pf*YRS reaction buffer consists of 25 mM Tris, pH 8, 150 mM NaCl, 5 mM $MgCl_2$ and 1 mM TCEP. The buffer for *Hs*YRS reaction consists of 50 mM Tris, pH 7.5, 150 mM NaCl, 50 mM KCl, 5 mM $MgCl_2$ and 1 mM TCEP. The mixture was incubated at 37°C for 3 h. After that, an equal volume of 8 M urea was added to the mixture. Finally, trifluoroacetic acid was added to a final concentration of 1%. The sample was centrifuged at 15,000 g for 10 min and the supernatant was used for mass spectrometry analysis. Synthetic ML901-Tyr standards were prepared in the same way at: 0.1 μM, 0.5 μM, 1 μM, 2 μM and 5 μM.

Identification of conjugates in cell cultures

A late trophozoite stage *P. falciparum* (3D7 strain (*43*)) culture was exposed to 3 μM ML901 or 10 μM AMS for 3 h. Following drug treatment, parasite-infected RBCs were lysed with 0.1% saponin and the parasite pellet was washed 3 times with ice-cold PBS. HeLa cells in suspension were seeded at 1 x 10^6 cells per 100 mm cell culture dish (3 dishes per treatment) and cultured for 48 h. c-DMEM media was replaced with 10 μM AMS in c-DMEM, or control c-DMEM alone for 2 h at 37°C. Cells were washed twice in PBS then detached from the dishes into a single cell suspension with TrypLE-Express enzyme (1x) (Gibco™, Thermo Fisher Scientific, Australia), washed in c-DMEM and pelleted at 400 x g, 4°C for 5 min, then washed twice in PBS. Cell pellets were kept on ice until processing for mass spectrometry.

P. falciparum and HeLa cell pellets were resuspended in water as one volume, followed by the addition of five volumes of cold chloroform-methanol (2:1 [vol/vol]) solution. Samples were incubated on ice for 5 min, subjected to vortex mixing for 1 min and centrifuged at 12,000 rpm for 10 min at 4°C to form 3 phases. The top aqueous layer was transferred to a new tube and subjected to LCMS analysis.

High-performance liquid chromatography (HPLC) and mass spectrometric (MS) analyses

Samples were analysed by reversed-phase ultra-high performance liquid chromatography (UHPLC) coupled to tandem mass spectrometry (MS/MS) employing a Vanquish UHPLC linked to an Orbitrap

Fusion Lumos mass spectrometer (Thermo Fisher Scientific, San Jose, CA, USA) operated in positive ion mode. Solvent A was 0.1% formic acid in water and solvent B was 0.1% formic acid in acetonitrile. 10 μL of each sample was injected into an RRHD Eclipse Plus C18 column (2.1 × 1000 mm, 1.8 μm; Agilent Technologies, USA) at 50 °C at a flow rate of 350 μL/min for 3 min using 3% solvent B. During separation, the percentage of solvent B was increased from 3% to 50% in 6 min. Subsequently, the percentage of solvent B was increased to 99% in 0.1 min and then maintained at 99% for 0.9 min. Finally, the percentage of solvent B was decreased to 3% in 0.1 min and maintained for 3.9 min.

MS experiments were performed using a Heated Electrospray Ionization (HESI) source. The spray voltages were 3.5 kV in positive ionization mode. The flow rates of sheath, auxiliary and sweep gases were 20 and 6 and 1 ‘arbitrary’ unit(s), respectively. The ion transfer tube and vaporizer temperatures were maintained at 350°C and 400°C, respectively, and the S-Lens RF level was set at 50%. A full-scan MS spectrum and targeted MS/MS for proton adduct of ML901-Tyr or 20 possible common amino acid-containing inhibitor adducts were acquired in cycles throughout the run. The full-scan MS-spectra were acquired in the Orbitrap at a mass resolving power of 120,000 (at m/z 200) across an m/z range of 200–1500 using quadrupole isolation and the targeted MS/MS were acquired using higher-energy collisional dissociation (HCD)-MS/MS in the Orbitrap at a mass resolving power of 7500 (at m/z 200), a normalized collision energy (NCE) of 20% and an m/z isolation window of 1.6.

Samples were also analysed using hydrophilic interaction liquid chromatography (HILIC) coupled to high-resolution mass spectrometry (Q Exactive, ThermoFisher). Samples (10 μL) were injected onto a Dionex Ultimate 3000 UHPLC system (ThermoFisher) and analytical separation was performed with a 150 mm × 4.6 mm, 5 μm ZIC-pHILIC column (Merck), using a guard column of the same material. Compounds were eluted at a flow rate of 300 μL/min using a binary gradient solvent system consisting of 20 mM ammonium carbonate pH 9 (solvent A) and acetonitrile (solvent B). The gradient profile was as follows: 0–15 min, 80–50% B; 15–18 min, 50–5% B; 18–21 min, 5% B; 21–24 min, 5–80% B and 24–32 min, 80% B. Full scan MS acquisition was performed in polarity switching mode, with the following settings: resolution 35,000, AGC target 1×10^6, *m/z* range 85–1275, sheath gas 50, auxiliary gas 20, sweep gas 2, probe temperature 120 °C, capillary temperature 300 °C and S-Lens RF level was set to 50. The spray voltage was set at 4 kV for positive ionization mode and 3.5 kV for negative ionization mode.

Crystallization and X-ray diffraction data collection

*Pf*YRS and *Hs*YRS were crystallized using the sitting drop vapor diffusion technique at 20°C. For *Pf*YRS crystals in complex with AMP-Tyr, crystals were formed in 2.25 M sodium malonate, pH 6. Drops contained 1.5 μL of protein-ligand solution (10 mg/mL *Pf*YRS in Tris-HCl (25 mM, pH 8)), 100 mM NaCl,

10 mM $MgCl_2$, 1 mM TCEP, 10 mM ATP and 1.2 mM tyrosine) and 1.5 µL of crystallant solution (2.25 M sodium malonate, pH 6).

Crystals of the *Pf*YRS/ML901-Tyr complex were generated by incubating the His-tagged protein (0.25 mg/mL) with 25 µM ATP, 50 µM tyrosine, 150 µM ML901 and 7 mg/ml *E.coli* tRNA. The complex was purified as described above. Crystals of purified complex were formed in 2.5 M sodium malonate, pH 6. Drops contained 1.5 µL of purified protein complex (7 mg/mL protein in Tris-HCl (50 mM, pH 8)), 150 mM NaCl and 1 mM TCEP) and 1.5 µL of crystallant solution (2.5 M sodium malonate, pH 6).

Crystals of the *Pf*YRS_{S234C}/ML901-Tyr complex were generated by adding 500 µM synthetic ML901-Tyr to the protein (5 mg/ml). Crystals of the complex were formed in 2.25-2.5 M sodium malonate, pH 6.5-7 with 2 mM TCEP. Drops contained 1.5 µL of the protein complex (in Tris-HCl (25 mM, pH 8)), 150 mM NaCl and 1 mM TCEP) and 1.5 µL of crystallant solution (2.25 M sodium malonate, pH 6.5). After 1 week, crystals were harvested and a seed stock created using the seed bead method (*63*). After seeding, crystals formed in 2.25 M sodium malonate, pH 6.5 with 2 mM TCEP, drops contained 1.5 µL protein solution, 1.5 µL crystallant and 0.5 µL seed.

A complex of *Hs*YRS with ML901-Tyr was generated by adding 500 µM synthetic ML901-Tyr to the protein (10 mg/mL). Crystals of the complex were formed in 2.1-2.3 M ammonium sulfate, 2% acetone, 0.1 M NaH_2PO_4/K_2HPO_4, pH 5.5. Drops contained 1.5 µL of the protein complex (in Tris-HCl (25 mM, pH 8)), 150 mM NaCl and 1 mM TCEP) and 1.5 µL of crystallant solution.

Crystals were flash-cooled in liquid nitrogen directly from the crystallization drop, and X-ray diffraction data were collected at 100 K and a wavelength of 0.9537 Å using the Eiger 16M detector at the MX2 beamline of the Australian Synchrotron (*64*). Diffraction data were indexed and integrated using XDS (*65*) and analysed using *POINTLESS* (*66*), prior to merging by *AIMLESS* (*67*) from the *CCP4* software suite (*68*). Initial phase estimates for *Pf*YRS in complex with AMP-Tyr were obtained by molecular replacement in *PHASER* (*69*) using modified crystal structure coordinates of *Pf*YRS as the search model (PDB ID: 3VGJ; (*19*)). Initial phase estimates for *Pf*YRS and *Pf*YRS_{S234C} in complex with ML901-Tyr were obtained using our *Pf*YRS/AMP-Tyr coordinates (PDB ID: 7ROR, this work). Initial phase estimates for *Hs*YRS in complex with ML901-Tyr were obtained using modified crystal structure coordinates of the structure of *Hs*YRS (PDB ID: 4QBT; (*20*)) as the search model. Automated structure refinement using *phenix.refine* (*70*) was followed iteratively by manual model building in *COOT* (*71*). Structure refinement was performed using translation/libration screw (TLS) refinement with each chain comprising a single TLS group. Restraints for AMP-Tyr and ML901-Tyr were generated using *phenix.elbow* (*72*). Final data collection and refinement statistics are shown in Suppl Table 8.

References

1. World_Health_Organisation, World Malaria Report 2021. https://www.who.int/publications/i/item/world-malaria-report-2021 (2021).
2. R. W. van der Pluijm *et al.*, Determinants of dihydroartemisinin-piperaquine treatment failure in *Plasmodium falciparum* malaria in Cambodia, Thailand, and Vietnam: a prospective clinical, pharmacological, and genetic study. *Lancet Infect Dis* **19**, 952-961 (2019).
3. B. Balikagala *et al.*, Evidence of artemisinin-resistant malaria in Africa. *New England Journal of Medicine* **385**, 1163-1171 (2021).
4. J. E. Brownell *et al.*, Substrate-assisted inhibition of ubiquitin-like protein-activating enzymes: the NEDD8 E1 inhibitor MLN4924 forms a NEDD8-AMP mimetic in situ. *Mol Cell* **37**, 102-111 (2010).
5. J. Florini, in *Antibiotics I: Mechanism of Action,* G. Shaw, Ed. (Springer, 1967), pp. 427 - 433.
6. K. Isono *et al.*, Ascamycin and dealanylascamycin, nucleoside antibiotics from *Streptomyces sp*. *J Antibiot (Tokyo)* **37**, 670-672 (1984).
7. H. Osada, K. Isono, Mechanism of action and selective toxicity of ascamycin, a nucleoside antibiotic. *Antimicrob Agents Chemother* **27**, 230-233 (1985).
8. A. Bloch, C. Coutsogeorgopoulos, Inhibition of protein synthesis by 5'-sulfamoyladenosine. *Biochemistry* **10**, 4395-4398 (1971).
9. J. R. Florini, H. H. Bird, P. H. Bell, Inhibition of protein synthesis in vitro and in vivo by nucleocidin, an antitrypanosomal antibiotic. *J Biol Chem* **241**, 1091-1098 (1966).
10. B. A. Castilho *et al.*, Keeping the eIF2 alpha kinase Gcn2 in check. *Biochim Biophys Acta* **1843**, 1948-1968 (2014).
11. S.-C. Huang *et al.*, Discovery and optimization of pyrazolopyrimidine sulfamates as ATG7 inhibitors. *Bioorganic & medicinal chemistry* **28**, 115681 (2020).
12. I. Angulo-Barturen *et al.*, A murine model of falciparum-malaria by in vivo selection of competent strains in non-myelodepleted mice engrafted with human erythrocytes. *PLoS One* **3**, e2252 (2008).
13. M. B. Jimenez-Diaz *et al.*, Quantitative measurement of *Plasmodium*-infected erythrocytes in murine models of malaria by flow cytometry using bidimensional assessment of SYTO-16 fluorescence. *Cytometry A* **75**, 225-235 (2009).
14. J. Liu, Y. Xu, D. Stoleru, A. Salic, Imaging protein synthesis in cells and tissues with an alkyne analog of puromycin. *Proc Natl Acad Sci U S A* **109**, 413-418 (2012).
15. J. L. Bridgford *et al.*, Artemisinin kills malaria parasites by damaging proteins and inhibiting the proteasome. *Nature Communications* **9**, 3801 (2018).
16. L. Solyakov *et al.*, Global kinomic and phospho-proteomic analyses of the human malaria parasite *Plasmodium falciparum*. *Nat Commun* **2**, 565 (2011).
17. X. L. Yang, R. J. Skene, D. E. McRee, P. Schimmel, Crystal structure of a human aminoacyl-tRNA synthetase cytokine. *Proc Natl Acad Sci U S A* **99**, 15369-15374 (2002).
18. A. G. Torres, O. Reina, C. Stephan-Otto Attolini, L. Ribas de Pouplana, Differential expression of human tRNA genes drives the abundance of tRNA-derived fragments. *Proc Natl Acad Sci U S A* **116**, 8451-8456 (2019).
19. T. K. Bhatt *et al.*, Malaria parasite tyrosyl-tRNA synthetase secretion triggers pro-inflammatory responses. *Nat Commun* **2**, 530 (2011).
20. M. Sajish, P. Schimmel, A human tRNA synthetase is a potent PARP1-activating effector target for resveratrol. *Nature* **519**, 370-373 (2015).
21. M. C. Lux, L. C. Standke, D. S. Tan, Targeting adenylate-forming enzymes with designed sulfonyladenosine inhibitors. *J Antibiot (Tokyo)* **72**, 325-349 (2019).
22. S. Adhikari *et al.*, Atg7 inhibitors and the uses thereof. *World Intellectual Property Organization* **WO/2018/089786 PCT/US2017/061094**, (2017).
23. J. J. Chen *et al.*, Mechanistic studies of substrate-assisted inhibition of ubiquitin-activating enzyme by adenosine sulfamate analogues. *J Biol Chem* **286**, 40867-40877 (2011).
24. M. A. Milhollen *et al.*, Treatment-emergent mutations in NAEβ confer resistance to the NEDD8-activating enzyme inhibitor MLN4924. *Cancer Cell* **21**, 388-401 (2012).

25. J. Straimer *et al.*, Drug resistance. K13-propeller mutations confer artemisinin resistance in *Plasmodium falciparum* clinical isolates. *Science* **347**, 428-431 (2015).
26. T. Paquet *et al.*, Antimalarial efficacy of MMV390048, an inhibitor of *Plasmodium* phosphatidylinositol 4-kinase. *Science translational medicine* **9**, (2017).
27. B. Baragaña *et al.*, A novel multiple-stage antimalarial agent that inhibits protein synthesis. *Nature* **522**, 315-320 (2015).
28. J. S. McCarthy *et al.*, Safety, tolerability, pharmacokinetics, and activity of the novel long-acting antimalarial DSM265: a two-part first-in-human phase 1a/1b randomised study. *Lancet Infect Dis* **17**, 626-635 (2017).
29. M. Y. Lim *et al.*, UDP-galactose and acetyl-CoA transporters as *Plasmodium* multidrug resistance genes. *Nat Microbiol* **1**, 16166 (2016).
30. A. M. Stickles *et al.*, Subtle changes in endochin-like quinolone structure alter the site of inhibition within the cytochrome bc1 complex of *Plasmodium falciparum*. *Antimicrobial Agents and Chemotherapy* **59**, 1977 (2015).
31. M. J. Smilkstein *et al.*, A drug-selected *Plasmodium falciparum* lacking the need for conventional electron transport. *Mol Biochem Parasitol* **159**, 64-68 (2008).
32. M. J. Delves *et al.*, A high throughput screen for next-generation leads targeting malaria parasite transmission. *Nature Communications* **9**, 3805 (2018).
33. J. Schalkwijk *et al.*, Antimalarial pantothenamide metabolites target acetyl-coenzyme A biosynthesis in *Plasmodium falciparum*. *Science translational medicine* **11**, (2019).
34. D. M. Walker *et al.*, *Plasmodium falciparum* erythrocytic stage parasites require the putative autophagy protein PfAtg7 for normal growth. *PLoS One* **8**, e67047 (2013).
35. N. Kirby *et al.*, Improved radiation dose efficiency in solution SAXS using a sheath flow sample environment. *Acta Crystallographica Section D Structural Biology* **72**, 1254-1266 (2016).
36. N. M. Kirby *et al.*, A low-background-intensity focusing small-angle X-ray scattering undulator beamline. *Journal of Applied Crystallography* **46**, 1670-1680 (2013).
37. T. M. Ryan *et al.*, An optimized SEC-SAXS system enabling high X-ray dose for rapid SAXS assessment with correlated UV measurements for biomolecular structure analysis:. *Journal of Applied Crystallography* **51**, 97-111 (2018).
38. R. P. Rambo, J. A. Tainer, Accurate assessment of mass, models and resolution by small-angle scattering. *Nature* **496**, 477-481 (2013).
39. J. Hall, A simple model for determining affinity from irreversible thermal shifts. *Protein Sci* **28**, 1880-1887 (2019).
40. R. Dai *et al.*, Fragment-based exploration of binding site flexibility in *Mycobacterium tuberculosis* BioA. *Journal of medicinal chemistry* **58**, 5208-5217 (2015).
41. P. Mujumdar, S. Bua, C. T. Supuran, T. S. Peat, S. A. Poulsen, Synthesis, structure and bioactivity of primary sulfamate-containing natural products. *Bioorg Med Chem Lett* **28**, 3009-3013 (2018).
42. J. D. Anderson, N. K. Dalley, G. R. Revankar, R. K. Robins, Synthesis of certain 3-alkoxy-1-β-D-ribofuranosylpyrazolo[3,4-d]pyrimidines structurally related to adenosine, inosine and guanosine. **23**, 1869-1878 (1986).
43. G. Lawrence *et al.*, Effect of vaccination with 3 recombinant asexual-stage malaria antigens on initial growth rates of *Plasmodium falciparum* in non-immune volunteers. *Vaccine* **18**, 1925-1931 (2000).
44. C. Dogovski *et al.*, Targeting the cell stress response of *Plasmodium falciparum* to overcome artemisinin resistance. *PLoS Biol* **13**, e1002132 (2015).
45. S. C. Xie, C. Dogovski, S. Kenny, L. Tilley, N. Klonis, Optimal assay design for determining the in vitro sensitivity of ring stage *Plasmodium falciparum* to artemisinins. *Int J Parasitol* **44**, 893-899 (2014).
46. C. Snyder, J. Chollet, J. Santo-Tomas, C. Scheurer, S. Wittlin, *In vitro* and *in vivo* interaction of synthetic peroxide RBx11160 (OZ277) with piperaquine in Plasmodium models. *Exp Parasitol* **115**, 296-300 (2007).
47. S. P. Crouch, R. Kozlowski, K. J. Slater, J. Fletcher, The use of ATP bioluminescence as a measure of cell proliferation and cytotoxicity. *J Immunol Methods* **160**, 81-88 (1993).

48. R. D. Petty, L. A. Sutherland, E. M. Hunter, I. A. Cree, Comparison of MTT and ATP-based assays for the measurement of viable cell number. *J Biolumin Chemilumin* **10**, 29-34 (1995).
49. J. Mandelbaum *et al.*, Identification of a lung cancer cell line deficient in atg7-dependent autophagy. *Autophagy*, 0 (2015).
50. T. A. Soucy *et al.*, An inhibitor of NEDD8-activating enzyme as a new approach to treat cancer. *Nature* **458**, 732-736 (2009).
51. M. B. Jiménez-Díaz *et al.*, Improved murine model of malaria using *Plasmodium falciparum* competent strains and non-myelodepleted NOD-scid IL2R gamma-null mice engrafted with human erythrocytes. *Antimicrobial agents and chemotherapy* **53**, 4533-4536 (2009).
52. M. J. Manary *et al.*, Identification of pathogen genomic variants through an integrated pipeline. *BMC Bioinformatics* **15**, 63 (2014).
53. A. McKenna *et al.*, The Genome Analysis Toolkit: a MapReduce framework for analyzing next-generation DNA sequencing data. *Genome Res* **20**, 1297-1303 (2010).
54. P. Cingolani *et al.*, A program for annotating and predicting the effects of single nucleotide polymorphisms, SnpEff: SNPs in the genome of Drosophila melanogaster strain w1118; iso-2; iso-3. *Fly* **6**, 80-92 (2012).
55. S. M. Ganesan, A. Falla, S. J. Goldfless, A. S. Nasamu, J. C. Niles, Synthetic RNA-protein modules integrated with native translation mechanisms to control gene expression in malaria parasites. *Nat Commun* **7**, 10727 (2016).
56. A. S. Nasamu *et al.*, An integrated platform for genome engineering and gene expression perturbation in *Plasmodium falciparum*. *Sci Rep* **11**, 342 (2021).
57. K. Deitsch, C. Driskill, T. Wellems, Transformation of malaria parasites by the spontaneous uptake and expression of DNA from human erythrocytes. *Nucleic Acids Res* **29**, 850-853 (2001).
58. M. Karpiyevich *et al.*, Nedd8 hydrolysis by UCH proteases in Plasmodium parasites. *PLoS Pathog* **15**, e1008086 (2019).
59. L. D. Cabrita *et al.*, Enhancing the stability and solubility of TEV protease using in silico design. *Protein science* **16**, 2360-2367 (2007).
60. P. Schuck, P. Rossmanith, Determination of the sedimentation coefficient distribution by least-squares boundary modeling. *Biopolymers* **54**, 328-341 (2000).
61. D. Franke *et al.*, ATSAS 2.8: A comprehensive data analysis suite for small-angle scattering from macromolecular solutions. *Journal of Applied Crystallography* **50**, 1212-1225 (2017).
62. C. Barberato, M. H. J. Koch, E. Molecular, H. Outstation, CRYSOL - a program to evaluate X-ray solution scattering of biological macromolecules from atomic coordinates. *Journal of Applied Crystallography* **28**, 768-773 (1995).
63. J. R. Luft, G. T. DeTitta, A method to produce microseed stock for use in the crystallization of biological macromolecules. *Acta Crystallographica Section D: Biological Crystallography* **55**, 988-993 (1999).
64. D. Aragão *et al.*, MX2: a high-flux undulator microfocus beamline serving both the chemical and macromolecular crystallography communities at the Australian Synchrotron. *Journal of Synchrotron Radiation* **25**, 885-891 (2018).
65. W. Kabsch, XDS. *Acta Crystallogr D* **66**, 125-132 (2010).
66. P. R. Evans, An introduction to data reduction: space-group determination, scaling and intensity statistics. *Acta Crystallogr D* **67**, 282-292 (2011).
67. P. R. Evans, G. N. Murshudov, How good are my data and what is the resolution? *Acta Crystallogr D* **69**, 1204-1214 (2013).
68. M. D. Winn *et al.*, Overview of the CCP4 suite and current developments. *Acta Crystallogr D* **67**, 235-242 (2011).
69. A. J. Mccoy *et al.*, Phaser crystallographic software. *Journal of Applied Crystallography* **40**, 658-674 (2007).
70. P. D. Adams *et al.*, PHENIX: a comprehensive Python-based system for macromolecular structure solution. *Acta Crystallogr D* **66**, 213-221 (2010).
71. P. Emsley, B. Lohkamp, W. G. Scott, K. Cowtan, Features and development of Coot. *Acta Crystallogr D* **66**, 486-501 (2010).

72. N. W. Moriarty, R. W. Grosse-Kunstleve, P. D. Adams, Electronic ligand builder and optimization workbench (eLBOW): A tool for ligand coordinate and restraint generation. *Acta Crystallographica Section D: Biological Crystallography* **65**, 1074-1080 (2009).
